# Biodiversity safeguards ecosystem services and functions worldwide

**DOI:** 10.1038/s41559-026-03200-4

**Published:** 2026-10-06

**Authors:** Emma R. Moffett, Joel H. Gayford, Lexi Chen, Olivia F. Morris, Yuming Shi, Lucy Somekh, Nicole Stasik, Andy Purvis, Guy Woodward, William D. Pearse

**Affiliations:** 1 https://ror.org/041kmwe10 grid.7445.2 0000 0001 2113 8111 The Georgina Mace Centre for the Living Planet, Department of Life Sciences, Imperial College London, Berkshire, UK; 2 https://ror.org/0220mzb33 grid.13097.3c 0000 0001 2322 6764 , Department of Geography, King’s College London, London, UK; 3 https://ror.org/04gsp2c11 grid.1011.1 0000 0004 0474 1797 Department of Marine Biology and Aquaculture, James Cook University, Townsville, Queensland Australia; 4 https://ror.org/039zvsn29 grid.35937.3b 0000 0001 2270 9879 Biodiversity Futures Lab, Natural History Museum, London, UK; 5 https://ror.org/05dhe8b71 grid.36212.34 Alan Turing Institute, British Library, London, UK

**Keywords:** Biodiversity, Macroecology, Ecological modelling, Climate-change impacts

## Abstract

Biodiversity loss threatens ecosystem services on which humanity depends, yet the strength, form and context-dependency of biodiversity–ecosystem service and biodiversity–ecosystem function relationships remain poorly quantified. Here we synthesize 423 studies to determine how biodiversity affects ecosystem functioning and service delivery. We show that regulating services, including water quality and pollination, often exhibit linear relationships, suggesting an additive role for diversity. While biodiversity enhances most functions and services, our effect-size analysis shows that oceanic ‘blue carbon’ sequestration is exceptionally sensitive, revealing how critical marine biodiversity is for climate mitigation. Conversely, services such as hazard regulation appear relatively insensitive to biodiversity, instead depending on a few functionally unique foundational species whose persistence nonetheless is interdependent on the wider community. Our new synthesis enables the forecasting of impacts, for example, that biodiversity loss will reduce pest regulation in countries experiencing population growth and intensive development, and it also highlights how ecosystem services might be used to anticipate future problems.

## Abstract (web summary)

An analysis of a global database of 423 studies and >200,000 data points outlines the strength and generality of biodiversity relationships with ecosystem functioning and services.

## Main

Ecological research highlights the close relationship between biodiversity and a wide range of critical ecosystem services and ecosystem functions, from pollination to carbon sequestration^1–4^. Anthropogenic pressures (for example, land-use change and introduction of exotic species) have driven an unprecedented number of species, estimated at 1 million, towards extinction, threatening ecosystem services^4–8^. While global policies aim to halt this decline^9,10^, effective action requires a quantitative and synthetic understanding of how biodiversity influences ecosystem functions and services. Here, we follow the ecosystem service classification framework of the Intergovernmental Science-Policy Platform on Biodiversity and Ecosystem Services^11^(IPBES), which recognizes that ecosystem functions (processes that occur within an ecosystem) often underpin or constitute ecosystem services when they contribute to human well-being. For example, primary production can be an ecosystem service when it supports food provision or climate regulation. Accordingly, we classify response variables as ecosystem services where they align with IPBES nature’s contributions to people categories or ecosystem functions, and as ecosystem functions when they do not always provide direct or indirect benefits to people, to ensure comprehensive coverage of key ecosystem processes. Many functions included here may also be described as ecosystem services in other contexts^12–14^, reflecting overlapping use of the terms in literature. We also chose to explicitly separate carbon sequestration from other services and to assess its across terrestrial and marine realms, given the interest in biodiversity as a climate-regulator^15^.

While the consequence of biodiversity declines on ecosystem functioning is well-established, the functional form and scalability of biodiversity–ecosystem service (BES) and biodiversity–ecosystem function (BEF) relationships remain a fundamental challenge. BEF and BES relationships are often classified simplistically as linear or saturating, with no clear consensus on the scalability of data to real-world patterns^1,16^. Whether these relationships are typically linear, implying all species including rare and/or pollution-sensitive taxa contribute equally, or saturating, suggesting widespread functional redundancy, is poorly resolved at a global scale^16–18^. For a limited set of processes, such as primary production in plant communities, mechanistic explanations of biodiversity effects, such as selection, complementarity or portfolio effects, are relatively well studied^19–21^. However, our understanding of the generality of the qualitative (linear versus saturating) and quantitative relationship between biodiversity and ecosystem services and functions is limited as it is unclear if these mechanisms apply to the full range of ecosystem services.

Foundational BEF research has often focused on ecosystems that are particularly amenable to experimental manipulation, such as small-scale experimental plots in grasslands^18,22^. Although recent studies have expanded the scope of BEF research across a wider range of ecosystems and spatial scales, a comprehensive global synthesis (that is, across ecosystem types and regions) is still lacking^3,23^. Here we present a new global database of 423 studies of biodiversity relationships with ecosystem functioning and services, more than twice as large as the next largest database^24^, representing a major expansion in ecological, methodological and geographical diversity (Fig. 1). This database combines experimental and observational evidence across terrestrial, freshwater, marine and estuarine systems, enabling, for the first time, a consistent global assessment of how biodiversity measurements (species number, functional group and taxonomic group) influence multiple categories of ecosystem service. Using this framework, we evaluate the functional forms of BES/BEF relationships, quantify their sensitivity to biodiversity loss, identify generalized factors shaping these patterns and model future trajectories in pest regulation, which may contribute to food and feed provision under alternative population growth and development scenarios.

**Fig. 1:**
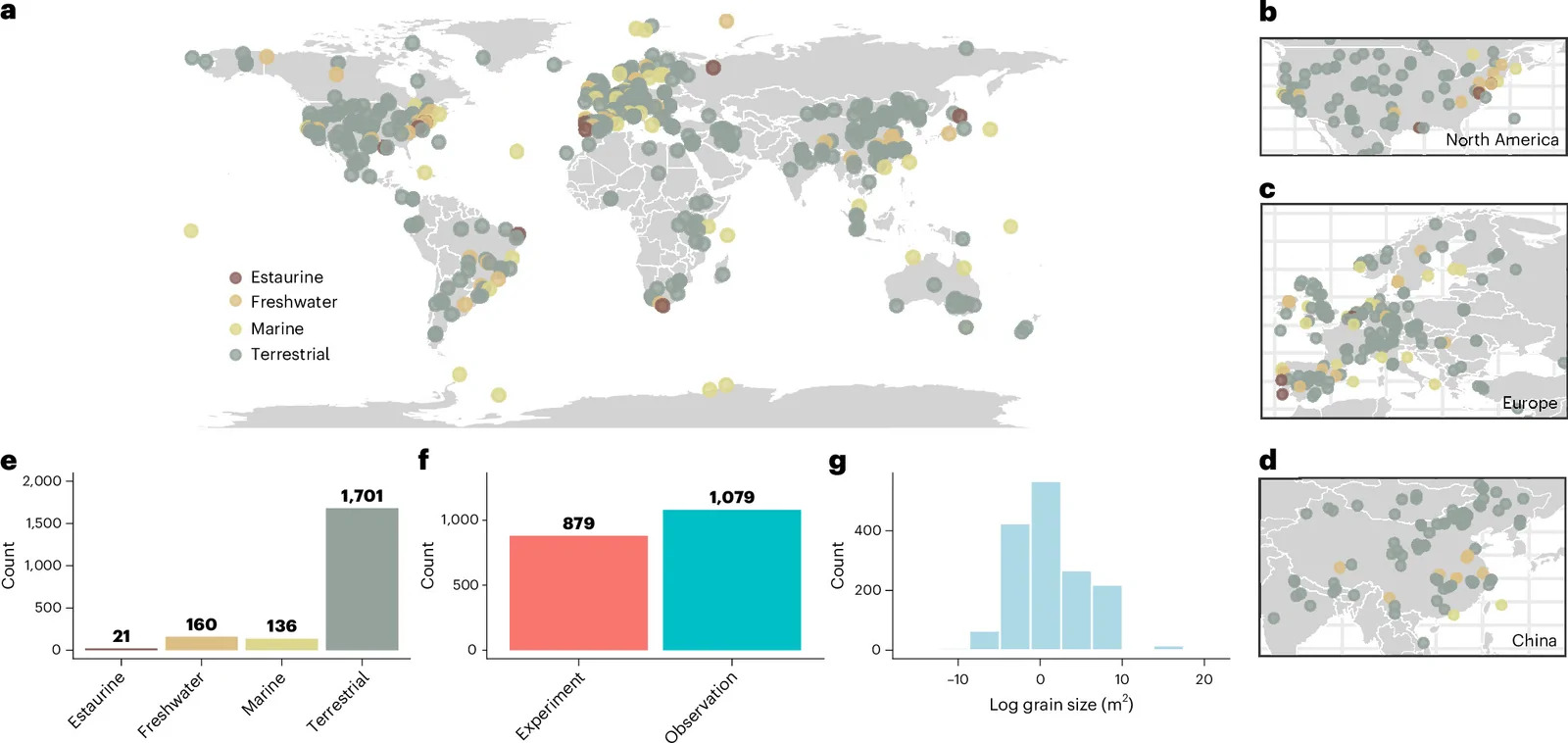
The type and distribution of BES and BEF data collated worldwide. **a**–**d**, Study sites used to analyse global BES and BEF trends (**a**), with studies colour-coded according to ecosystem type and inserts showing North America (**b**), Europe (**c**) and China (**d**). Study sites are predominantly terrestrial and located in developed countries. **e**, There are 1,759 datasets from terrestrial studies compared to 183 in freshwater, 153 in marine and 23 in estuarine systems. **f**, There are 20% more observational (*n* = 1,079) datasets in our database compared with experimental (*n* = 879) datasets. **g**, Grain size ranged from 0.0000007 m^2^ to 260,018,758 m^2^ with a median value of 1 m^2^ across datasets. World map data from Natural Earth (https://www.naturalearthdata.com/). Source data

## Results

### Functional variation in BES and BEF relationships

While positive BES and BEF relationships are commonly described^16,25^, the functional forms of biodiversity effects remain poorly quantified across and within ecosystem services categories^11^. Across 23 categories of BES and BEF relationships, quadratic (*n* = 11) and linear (*n* = 9) relationships were most prevalent, with only two best-described by a logarithmic model, suggesting that most ecosystem services continue to increase rather than saturate at low levels of biodiversity (Fig. 2).

**Fig. 2:**
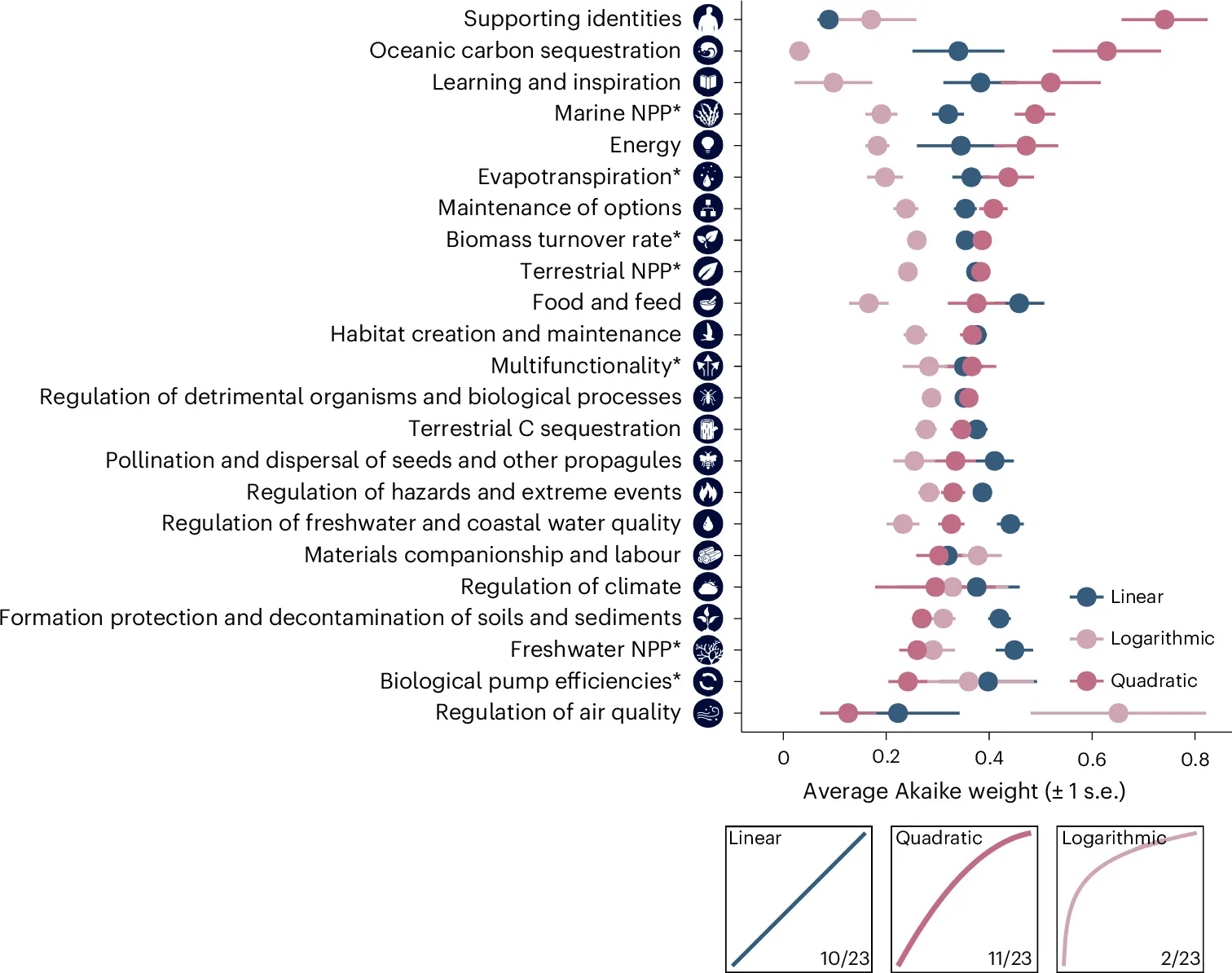
Linear biodiversity relationships with ecosystem services and functions are almost as common as saturating (quadratic or logarithmic) ones. Dataset count values are shown in Fig. 3b. The relative likelihood that an ecosystem service category (rows; measured by Akaike weights with error bars representing ±1 standard error (s.e.)) is fit by linear and saturating (quadratic or logarithmic) models. A high average Akaike weight value indicates a greater likelihood that the model type best fits the data than other model types. Most BES and BEF relationships are best fit by quadratic (11/23) or linear (10/23) models, whereas only two are best fit by a logarithmic model (2/23). We note that these results are not induced by a splitting of variance among two saturating models; we find qualitatively identical results when comparing the best-fitting saturating model with linear (46% supporting linear and 54% supporting saturating). Data are sorted by the highest to the lowest mean value of the quadratic model. Response variables marked with an asterisk (*) are classified as ecosystem functions; all other response variables are ecosystem services^11^. Source data

Although saturating relationships are often reported^3,26–28^, we find that linear relationships are also common, with their prevalence varying among ecosystem services and ecosystem functions categories. For some functions, such as terrestrial net primary productivity (NPP), different models performed equally well, suggesting contextual factors (for example, region and study type) modulate the form of the relationship. Variation in BES and BEF relationships partly reflects methodological choices as biodiversity metric classes differed among ecosystem services (Cramér’s *V* = 0.34), and the form of relationships varied with metric type, with functional and phylogenetic diversity metrics more often producing saturating responses whereas taxonomic diversity evenly distributed across linear and saturating models (Extended Data Fig. 1). Importantly, explanatory power varied substantially among ecosystem service and function categories and among model forms. Most biodiversity–ecosystem relationships showed low-to-moderate *r*^2^ values (for example, *r*^2^ < 0.4; Supplementary Fig. 1), indicating that biodiversity is an important but context-dependent driver of ecosystem functioning and service delivery. However, explanatory power remained weak for some services, such as hazard regulation, implying stronger influence of environmental context or functionally unique species.

Regulating services, including water quality, hazards and pollination, were typically linear, indicating a broad equivalent role for biodiversity (for example, species number, functional group or taxonomic group) in maintaining them. By contrast, oceanic carbon sequestration and learning and inspiration exhibited saturating relationships, indicating higher functional redundancy among species. While uneven data distribution, particularly uneven representation across ecosystem types and spatial grain (Fig. 1), and across ecosystem service and ecosystem function categories may influence these findings, the observed variation in BES and BEF relationships, including within well-studied categories, suggests that some services follow linear relationships while others tend towards saturation. Any changes in biodiversity are expected to affect ecosystem services and function when biodiversity levels fall below the saturation point. The prevalence of linear and saturating relationships suggests that some ecosystem services and functions peak only at the highest observed levels of biodiversity, underscoring the importance of the richest communities. However, linear trends may either reflect limited sampling of high-biodiversity systems or genuinely linear ecological responses in which linear relationships may be intrinsic to some ecosystem service and function types.

No ecosystem service or function can rise indefinitely with biodiversity, but our data suggest saturation limits have rarely been reached within the observed biodiversity gradients in the empirical literature, implying functional redundancy may be overestimated. In other words, individual species contribute more uniquely to ecosystem functioning, and ecological uniqueness plays a key role in sustaining service delivery, consistent with the wide variation in saturation levels across ecosystems observed. However, in some systems, saturation is reached relatively quickly, such as decomposition in freshwater systems, which may saturate at low levels of biodiversity^29^

For saturating relationships, the asymptote, where additional species no longer substantially increase ecosystem service or function, at a median of ~4 (± median absolute deviation of 2) species in freshwater, ~14 (±9) in terrestrial and ~23 (±17) in marine ecosystems (Extended Data Fig. 2). Faster saturation in freshwater systems may reflect their smaller scale and higher functional redundancy, particularly as they are characterized by relatively generalist food webs, whereas the vast scale and high trophic complexity and prevalence of mutualisms and co-evolutionary interactions in marine systems, such as coral reefs, may require higher biodiversity to sustain function^1,30–33^. Marine systems may also display low species multifunctionality, meaning that individual ecosystem services can often be supported by a small subset of species, but maintaining a wide range of ecosystem services requires a high overall diversity^33^. While our data show differences in median saturation values across ecosystem types, there was a wide range of saturation values, indicating that differences in experimental design, scale and habitat may also shape BES and BEF relationships (see the ‘Influence of spatial grain, ecosystem type and study type on BES and BEF trends’ for more details), warranting closer examination in future studies.

### Quantifying biodiversity effects on ecosystem services and ecosystem functions

The variation in functional BES and BEF forms make it challenging to understand how ecosystem service and function categories respond to biodiversity. To overcome this, we use a second approach that allows us to explore relationships across forms. To our knowledge, this is the first analysis to quantify BES and BEF relationships across multiple functional forms using a standardized effect-size framework. We calculated the effect size, as the response of services and functions to increasing biodiversity (Fisher’s *Z*-transformed correlation coefficient), for each dataset (Methods; *n* = 1,959)^34^.

Higher biodiversity enhanced most BES and BEF, from carbon capture to food production (Fig. 3). While the magnitude of this effect varied across ecosystem service and function categories (regulation of air quality, Fisher’s *z* score (Zr) −0.03, very weak; oceanic carbon sequestration, Zr 1.48, very strong), the dependence of critical regulatory services on biodiversity, until the point of saturation, is unequivocal. The imperative to foster and safeguard biodiversity is particularly acute for marine ecosystems. Oceanic carbon sequestration, which is a significant part of the global carbon cycle, exhibits by far the strongest positive response to biodiversity of any service or function category^35^. This strong sensitivity may reflect the strong biological control of marine carbon cycling, including phytoplankton productivity, microbial loop dynamics and the biological carbon pump, all of which are closely linked to community composition and trophic structure. However, this result should be interpreted cautiously, as oceanic carbon sequestration is relatively underrepresented (*n* = 7), with far fewer datasets in our database than for terrestrial carbon sequestration (*n* = 154), highlighting a critical gap in current knowledge (Fig. 3). This exceptional sensitivity implies that blue carbon sinks are both vulnerable to biodiversity loss and dependent on sustaining diverse communities^36,37^. Maximizing the efficacy and long-term viability of blue carbon strategies will require moving beyond habitat preservation towards explicitly incorporating biodiversity as a key component^38,39^.

**Fig. 3:**
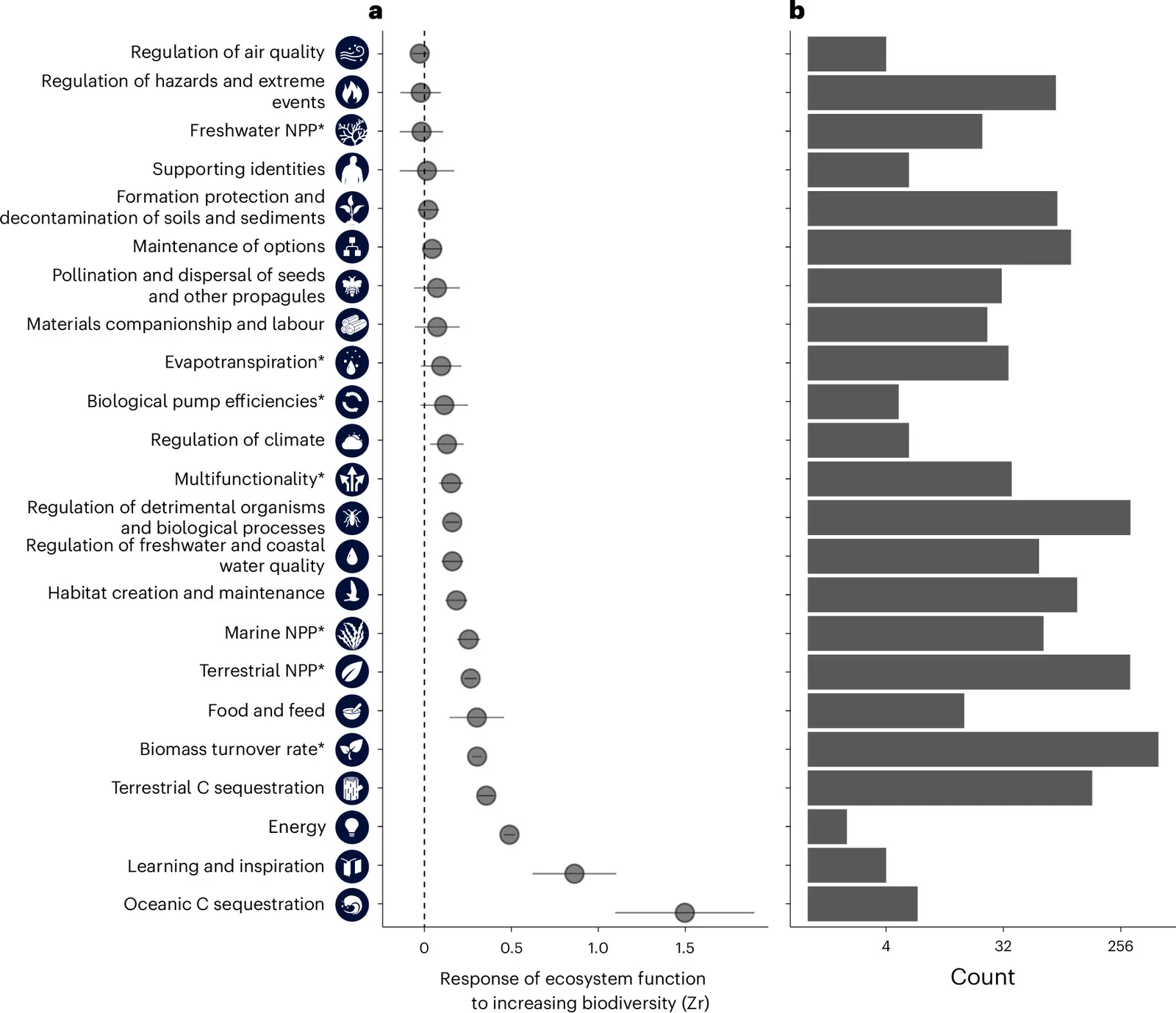
Biodiversity loss has uneven consequences for ecosystem functioning and service provision. **a**,**b**, Comparison of biodiversity responses among ecosystem service and function categories, as effect size (Zr) (**a**) and the number of datasets used to calculate effect size (**b**). Effect size data are average ±1 standard error (s.e.), calculated on datasets within each ecosystem service category, where a high effect size value indicates a stronger effect of biodiversity on ecosystem service compared with a lower value. See Extended Data Fig. 4 for effect sizes split by study type (experiment versus observation). Higher biodiversity enhanced most ES, from carbon capture to food production, in particular oceanic and terrestrial carbon sequestration were strongly dependent on biodiversity. Conversely, some services, such as hazard regulation, appeared insensitive to biodiversity. Notably, these effects vary by habitat type, spatial grain and study type. Response variables marked with an asterisk (*) are classified as ecosystem functions; all other response variables are ecosystem services^11^. Source data

Conversely, some services, such as hazard regulation, exhibited weak overall sensitivity to biodiversity in the effect-size analysis, despite often showing linear relationships in the functional form analysis (*Zr* = 0, *r*^2^ = 0.17; Figs. 2 and 3 and Supplementary Fig. 1). This may reflect functional uniqueness, where service provision is dominated by a few foundational species or ecosystem engineers that define the ecosystem itself. For example, sand dunes are stabilized by one or two key shrub species that create a natural barrier against flooding and erosion^40^. Here, the service is provided by the foundational species, and the system’s direct performance in this role is not necessarily enhanced by the addition of other species. However, maintaining broader biodiversity may still be important for sustaining the conditions that allow these foundational species to persist and function effectively^38,39,41–43^. In systems governed by functionally unique species, maintaining ecosystem services and functions may rely more on the persistence of these taxa than on biodiversity per se. This approach aligns with the concept of ecosystem-specific ‘planetary boundaries,’ which are thresholds of control variables that, when crossed, can lead to the collapse of ecosystem functions^44^. Identifying a priori which systems are shaped by uniqueness versus redundancy is a critical challenge. Our data suggest that developing a typology of species and system traits is a promising avenue for future research to guide conservation priorities. Beyond further data synthesis, progress will depend on a new generation of targeted field experiments explicitly designed to disentangle the roles of uniqueness and redundancy in sustaining ecosystem functioning, thereby providing direct empirical tests of these theoretical predictions.

### Influence of spatial grain, ecosystem type and study type on BES and BEF trends

Effect-size analyses offer robust insights into BES and BEF relationships, but they do not fully explain variation depending on ecosystems’ context. Contextual factors, such as spatial grain (that is, the spatial resolution at which biodiversity and ecosystem services are measured, from fine-scale plots to broader landscape units), ecosystem type and study type, may influence both the strength and form of biodiversity relationships with ecosystem services and functions. This is because ecological processes, including species interactions and environmental heterogeneity, operate differently across spatial scales, potentially amplifying or obscuring biodiversity effects. Understanding such patterns is essential to interpreting the generality of BES and BEF findings and improving forecasts under global change. Using a linear mixed-effect model, we tested for BES and BEF relationships with spatial grain, ecosystem type and study type as factors (Methods and Supplementary Table 1).

The influence of spatial grain on BES and BEF was highly context dependent. Across all ecosystem services and functions, biodiversity effects strengthened with increasing grain (*β* = 210.26, *P* < 0.001; Supplementary Table 1), indicating that its contributions become more apparent at broader spatial scales. This pattern may partly reflect a beta-diversity effect, whereby spatial turnover in species composition enhances functional complementarity across sites, amplifying aggregate ecosystem service delivery at coarser grains, as predicted by spatial insurance and complementarity theories. However, responses varied substantially among ecosystem services and functions. For example, biodiversity contributions to regulation of hazards and extreme events, NPP and biomass turnover all declined significantly with increasing spatial grain (Supplementary Table 1). This scale-dependence is consistent with forest plot studies where positive BES and BEF relationships at fine grains (for example, 20 m^2^) can become negative at coarser grains (for example, 50 m^2^)^45^. Averaging over larger areas probably masks the fine-scale heterogeneity and species interactions that fuel ecosystem service and function provision^27,39^. Consequently, failing to account for measurement scale can fundamentally alter conclusions about ecosystem stability and responses to environmental change.

Second, ecosystem type significantly influenced BES and BEF relationships and highlighted data gaps in literature. Although our literature review was not designed to quantify ecosystem type biases per se, the differences we found in sampling, particularly the sparse coverage of freshwater and marine systems, are unlikely to reflect sampling artefacts alone but rather systematic biases in research focus and accessibility, with terrestrial systems historically receiving disproportionate attention (Extended Data Fig. 3 and Supplementary Table 1). Overall, biodiversity effects were positive in freshwater systems (*β* = 0.34, *P* = 0.01), negative in marine systems (*β* = –0.25, *P* = 0.016) and not significant in terrestrial systems (*P* > 0.05). However, these averages mask strong variation across ecosystem service categories. For example, biodiversity significantly enhanced biomass turnover rate in marine systems, reduced it in freshwater systems and had no effect in terrestrial systems. By contrast, for maintenance of options, biodiversity effects were consistently positive in both freshwater and marine environments. Such differences may reflect underlying ecological processes, such as higher functional redundancy, or may arise from sampling and design biases. These patterns should be interpreted in the context of uneven sampling across the global BES and BEF literature, with strong representation of terrestrial systems and fine spatial grains (for example, quadrats and individual plots), and limited coverage of freshwater and marine ecosystems and broader-scale studies (for example, landscapes and regions) (Fig. 1 and Supplementary Table 4). While overall biodiversity effects are robust, some ecosystem differences and functional forms may shift as additional data become available from undersampled systems and scales.

Finally, observational studies revealed modest but statistically stronger biodiversity effects than experiments (*β* = 0.03, *P* = 0.005), demonstrating that study type significantly shapes BES and BEF trends. This overall effect size is small relative to other predictors in our models and should be interpreted as highlighting service-specific contrasts rather than a universal shift per se. Biodiversity effects were pronounced in observational studies for multifunctionality (*β* = 0.48), pollination and dispersal of seeds and other propagules (*β* = 0.37) and NPP (*β* = 0.19). Effects were weaker and negative for biomass turnover rate (*β* = –0.03) and regulation of hazards and extreme events (*β* = –0.13), with no detectable differences for several other services, including evapotranspiration and food and feed (Supplementary Table 1 and Extended Data Fig. 4). These findings indicate that experiments are critical for revealing mechanisms such as complementarity, overyielding and sampling effects but typically operate at small scales and simplified conditions potentially underestimating cumulative or context-dependent impacts^46–49^. Observational studies capture broader spatial and temporal scales, larger biodiversity gradients (including rare and functionally unique species) and interactions among multiple biotic and abiotic factors.

Future progress will rely on combining global syntheses with coordinated, cross-system experiments that explicitly test how biodiversity scales through space, time and function. Priorities include creating hybrid experimental and observational networks that embed manipulations within long-term monitoring frameworks (for example, plots scale manipulations on reef patches and survey data collected at both local and regional scales); hierarchical sampling designs to quantify α-, β- and γ-diversity contributions to service delivery; and standardized experimentation across ecosystem types to elucidate mechanisms, generalities and context-dependant differences.

### Predicting BES and BEF relationships

In the effect size analysis above, we demonstrate that multiple ecosystem services and functions, including carbon sequestration, biomass turnover rates and food and feed provision, exhibit significant sensitivity to biodiversity loss. Using these data, we predict future shifts in biodiversity relationships with a key ecosystem service. Existing literature is geographically biased towards developed countries (Fig. 1a), underrepresenting regions critical for global food production, such as South America and Africa^50^. To address this gap, we developed a novel heuristic modelling framework that links global BES and BEF relationships with projected changes in land use and associated anthropogenic pressures. By integrating socioeconomic scenario-based biodiversity projections from the Biodiversity Intactness Index (BII; derived under the Intergovernmental Panel on Climate Change (IPCC) Shared Socioeconomic Pathways (SSPs))^51,52^, and our database, we generate spatially explicit projections of biodiversity-mediated changes in ecosystem service delivery. This framework enables identification of regions where biodiversity loss may most strongly affect ecosystem functioning.

We apply our modelling framework to a case study on agricultural pest regulation, a globally critical issue for food security. This focus aligns with the predominance of terrestrial data in our database and the growing attention on agricultural sustainability^53,54^. In particular, we examined the ratio of natural enemies to pests, a widely used proxy for top-down biological control across heterogeneous ecological systems^53–55^. We forecast changes in this ratio as an integrated measure of biological pest regulation rather than modelling changes in natural enemies or pest populations separately. Top-down biological control of agricultural pests by natural enemies supports crop production through the regulation of pest populations and is recognized as a key regulating Nature’s Contribution to People in global assessments by IPBES^11^. Accordingly, changes in predator–pest dynamics represent an important mechanism through which biodiversity influences agricultural production under global environmental change. To link our BES model with large-scale projections of biodiversity, we scaled BES data to BII^56^ data to estimate future changes in the ratio of natural enemies to pests and associated pest control services. The BII projections capture biodiversity responses to anthropogenic pressures, primarily land-use change, under different socioeconomic scenarios. Accordingly, projected regional differences primarily reflect variation in land-use intensity, biodiversity intactness and socioeconomic vulnerability embedded within SSP scenarios rather than direct climate effects on pest or predator dynamics. We find that regions with rapid population growth are projected to experience greater pressure on agricultural systems and stronger declines in biodiversity-mediated pest regulation. We focus on two SSPs^51^: SSP2, a ‘middle-of-the-road’ trajectory reflecting continuation of current socioeconomic trends, and SSP5, a fossil fuel-driven trajectory characterized by intensive resource exploitation.

Our projections suggest that biological pest regulation services relevant to food and feed production are reduced under fossil fuel development compared with the middle-of-the-road development trajectory, though the impacts are heterogeneous (Fig. 4a,b and Supplementary Fig. 2) and particularly pronounced in regions experiencing rapid population growth and with low Human Development Index (HDI) values (Fig. 4c,d and Supplementary Table 2). HDI is a composite measure of national development based on life expectancy, education and income. Countries with low HDI typically experience higher reliance on local ecosystem services and more rapid population growth compared with high HDI countries. As such, HDI provides a useful proxy for socioeconomic vulnerability and dependence on biodiversity-mediated ecosystem services^57^.

**Fig. 4:**
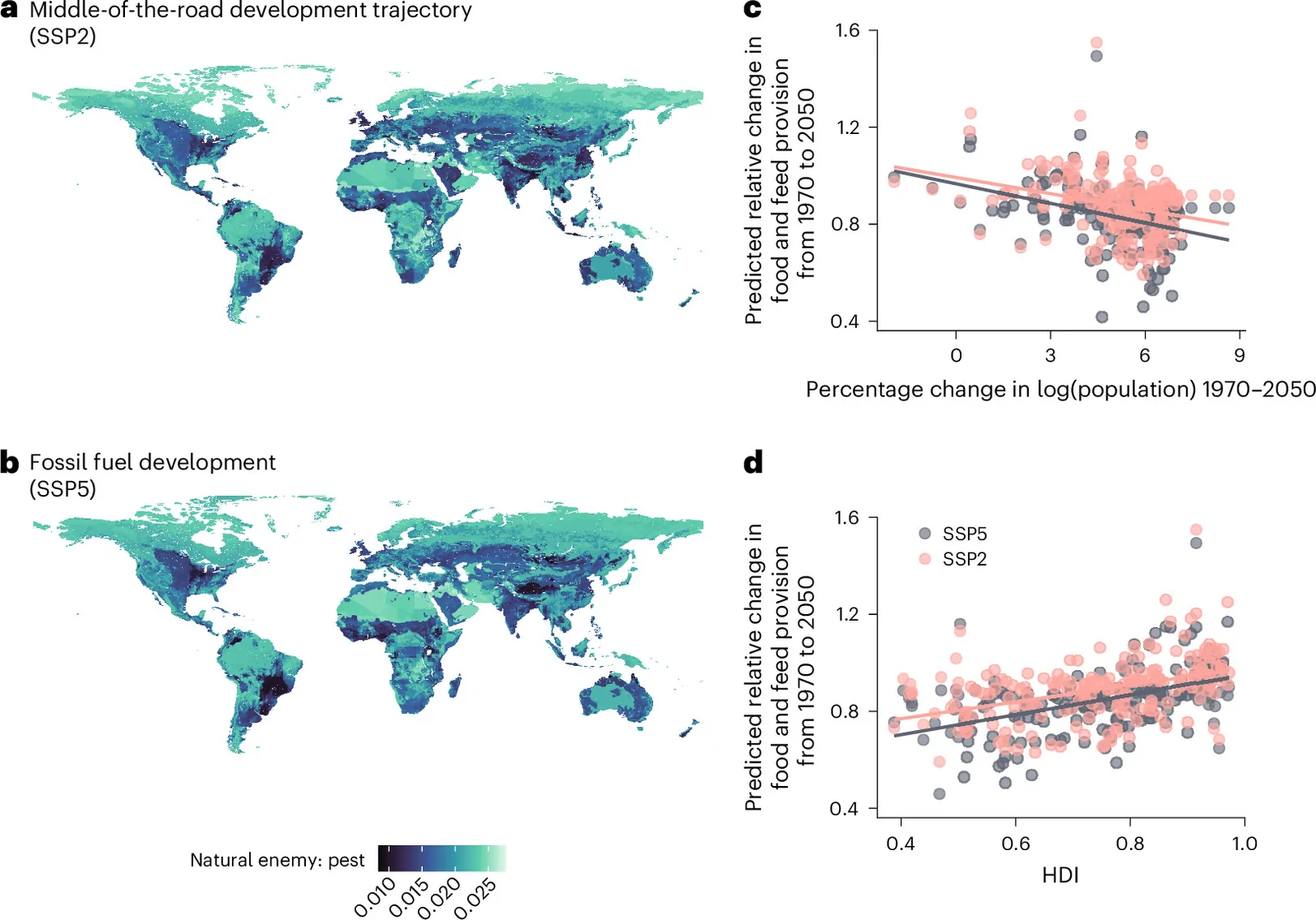
Forecasting ecosystem services helps in planning to meet future human demands. **a**,**b**, Here, we combine our BES data with two IPCC shared sociopolitical climate change scenarios (SSPs)^45^ and their resulting biodiversity intactness values^46^ under middle-of-the-road (**a**) (SSP2) and fossil-fuel development (**b**) (SSP5) scenarios (see Supplementary Fig. 2 for all scenarios). While the differences between these two scenarios for biodiversity are complex, our model forecasts that under each scenario, there will be subtly different degrees of protection provided from pests by natural enemies (**a** and **b**). **c**,**d**, Results from two-sided SAR regression models accounting for spatial autocorrelation between regions (Methods). A decrease in natural protection from pests would be expected to make agricultural production more difficult, and these changes will disproportionately affect countries experiencing greater population growth and with low HDI values (**c** and **d**). No adjustment for multiple comparisons was applied. Predicted relative changes in food and feed at the country level between 1970 and 2050 are plotted against the percentage change in population between 1970 and 2050 (**c**) and the human development index, HDI (**d**), for both SSP2 and SSP5. We found that pest protection is reduced under fossil fuel-driven development (SSP5; *β* = –0.0488 ± 0.0105, *z* = –4.65, *P* = 3.34 × 10^−6^), and we observe a disproportionately greater decline in faster-growing countries (*β* = –0.0088 ± 0.0021, *z* = –4.28, *P* = 1.85 × 10^−5^; Supplementary Table 2). Pest protection is also reduced in countries with low HDI values (*β* = 0.176 ± 0.038, *z* = 4.59, *P* = 4.41 × 10^−6^) which is further exacerbated under fossil fuel development (*β* = –0.049 ± 0.011, *z* = –4.60, *P* = 4.27 × 10^−6^; Supplementary Table 3). This case study highlights the importance of considering forecasting not only climate or biodiversity but also ecosystem services when considering future decision-making. World map data from Natural Earth (https://www.naturalearthdata.com/). Source data

Our model predicts reductions in the natural enemy to pest ratio under SSP5 relative to SSP2 in North America, South America and Asia, whereas Australasia and Africa show comparatively minor differences between scenarios. These results suggest that biodiversity loss may weaken an important ecological pathway underpinning agricultural production, particularly in regions facing high demand for food systems. By contrast, countries with higher HDI values are predicted to experience comparatively greater levels of biological pest regulation potential, reflecting greater capacity to maintain biological pest regulation. An important limitation is that projected BII changes do not account for which species are lost under future scenarios. If species vulnerability is correlated with their functional contributions, future ecosystem service changes may differ from our projections. Future integration of species-level vulnerability and functional traits will therefore be important for assessing how non-random biodiversity change influences BES relationships. Importantly, our framework enables these regional and scenario-based differences to be quantified and compared on a common scale, providing a scientifically grounded basis for assessing biodiversity-driven changes in ecosystem services. We release model outputs and forecasts across all 23 ecosystem services and functions and five SSPs in the supplementary materials to support policy and planning.

### Conclusion

Our analysis, leveraging the largest global synthesis of its kind, demonstrates that biodiversity underpins a wide range of ecosystem functions and ecosystem services, with biomass turnover, carbon sequestration and food and feed provision showing particularly strong sensitivity to high biodiversity. Exceptional sensitivity of oceanic carbon sequestration underscores the urgency of marine conservation. By combining this synthesis with a heuristic modelling framework, we predict how biodiversity loss may alter key ecosystem functions and services under future socioeconomic and land-use scenarios, identifying regions and services most at risk. Distinguishing between functional redundancy and functionally unique species, we show that conservation priorities must balance maintaining overall diversity with safeguarding irreplaceable foundational taxa. Contextual factors, including study type, ecosystem type and spatial grain, modulate biodiversity effects, highlighting the importance of context-dependent patterns. Integrating global syntheses with targeted cross-system experiments, standardized trait and service metrics and hierarchical sampling will be essential to resolve mechanisms and improve predictions of ecosystem service provision under global change.

## Methods

This study presents a comprehensive global analysis of the relationship between biodiversity and ecosystem services and biodiversity ecosystem functions, aiming to elucidate the impacts of biodiversity loss on critical ecological processes worldwide through the synthesis of a substantial body of existing BES and BEF research. We first investigated functional variation in BES and BEF relationships to assess the prevalence of linear versus saturating forms, the results of which underscore the consistent importance of high biodiversity levels. Subsequently, we quantified the magnitude of biodiversity’s influence on various functions, with particular emphasis on vital services such as carbon sequestration. Our analysis then explored factors influencing BES and BEF trends, including spatial grain, ecosystem type and study type, to understand their effects on BES and BEF relationships across diverse ecosystems and research methodologies. Finally, we predicted BES and BEF relationships under projected future scenarios, using IPCC climate projections and biodiversity forecasts to model potential consequences for an ecosystem service critical to human well-being: food provision^58^. All data analysis and statistical modelling were performed using the R programming language (R-4.4.2^59^), and the complete database is made publicly available. We express gratitude to the numerous researchers whose dedicated efforts and invaluable contributions were fundamental to this global synthesis; a comprehensive list of all contributors is provided in Supplementary Table 4.

### Stratified literature survey

We downloaded BES and BEF literature from the Web of Science (WoS) using the following search string: TS = (‘ecosystem service*’ OR ‘ecosystem function*’ OR (‘ecosystem stability’ AND ‘biodiversity’) OR ‘species richness’ OR ‘species diversity’). Given the diversity of metrics and terminology used in this field, relevant studies may not always be captured by strictly nested search strings. All records were therefore screened to ensure that only studies explicitly linking biodiversity to ecosystem services or functions were retained. This search resulted in 312,291 publications. To reduce the number of papers while ensuring a representative spread of data, we used a stratified survey method.

After first filtering the literature search results by ecosystem type and ecoregion. Within each stratum, records were ranked using the WoS relevance algorithm (based on keyword matching rather than citation counts), and the top 100 studies were selected for screening, resulting in 3,875 papers. Selecting a fixed number of highly relevant studies per ecosystem and ecoregion category allowed consistent screening and detailed data extraction within practical constraints. In addition, we carried out a specific search for Biodiversity OR species richness OR species diversity and each of the top ten most reported ES, as defined by the IPBES^60^, based on a WoS keyword search (energy, regulation of climate, food and feed, regulation of air quality, NPP, maintenance of options, regulation of freshwater and coastal water quality, biomass turnover rate and evapotranspiration). We added the top 100 most relevant papers for each of these ten ecosystem services categories to our literature database, meaning our stratified survey resulted in 4,875 papers (Supplementary Fig. 3).

When adding studies to our database, study relevance was not determined on the basis of study outcomes or statistical significance but strictly on the basis of predefined inclusion criteria. Studies were included if they (1) explicitly quantified biodiversity (for example species richness, diversity indices, functional or phylogenetic diversity) and (2) reported empirical relationships with at least one ecosystem function or service metric. Studies were excluded if they were purely theoretical, did not include quantitative biodiversity measures or did not report a direct BEF/BES relationship. Where multiple results were available within a study (for example, across ecosystem types, regions or services), all eligible effect sizes that met the inclusion criteria were extracted (Supplementary Fig. 3).

As our literature review method was not exhaustive, it was not designed to quantitatively measure biases in data collection across ecosystem type or ES. However, we would not expect the paper-selection processes to select certain systems over others, as data across regions and ecosystem type was explicitly considered. Our literature sorting was intended to be blind with respect to geographic biases, but we acknowledge that, given the geographic biases shown in Fig. 1, these may have crept into our analysis. We suggest, however, that the distribution of data most likely represents the underlying biases in funding and research into BES and BEF relationships. For example, it is quite clear from Fig. 1 that there is a bias towards Europe, and particularly Germany. We think it is unlikely that there is a reason that Germany should be highlighted by our search terms, instead its prominence likley reflects the presence of a number of key experiments (for example, Jena, BIODEPTH^22,61^).

While most of the ecosystem services categories reported are commonly used in the biodiversity literature, some are less well known such as ‘maintenance of options’, which refers to the ‘capacity of ecosystems, habitats, species or genotypes to keep options open to support a good quality of life’, or ‘biological pump efficiencies’, which concerns the transport of carbon from surface water into deeper water and sediments where the residence time is much longer^60^. The abstract and keywords of each paper in our stratified survey were screened for BES and BEF data. If a paper was relevant, we extracted raw data where possible; if raw data were unavailable, we extracted average and standard deviation values for each condition. We used Web Plot Digitiser, in-text tables, supplementary information or online data repositories to extract BES and BEF data. Papers whose data were unavailable were discarded (Supplementary Fig. 3).

From each of the papers included in our database, we collected data on environmental context (ecosystem type, location, experimental treatment, land use, GPS, spatial grain or the size of the unit of measurement) and any experimental manipulation (that is, temperature and biodiversity) and measurement type (experimental and observational). Land-use categories were defined by IPBES^54^. In total, our database includes data from 545 papers.

### Data analysis

All analyses were carried out using R (v. 4.4.2). Frequently, a given study would measure multiple functions or services, measure across different experiments within a broader study or within sites within a wider region. To separate and distinguish among these groups so that we could assess those, while also being able to statistically control for differences among studies, we assigned studies with multiple measurements of biodiversity, ecosystem service or function, location or taxon dataset numbers. We use these dataset numbers for analysis. Within each dataset, we filtered out BES and BEF data with fewer than six data points for either ecosystem service or function or biodiversity measurement. After filtering, our database includes 222,829 data points from 1,959 unique datasets extracted from 423 unique papers. The study selection and screening process is summarized in detail in the PRISMA flow diagram (Supplementary Fig. 3).

Data were standardized to control for different measurement units across studies using the scale function in R, with the standardized data used to calculate Akaike information criterion (AIC) scores and effect size values (see below). Rescaling our biodiversity and ecosystem data allowed us to control for differences in measurement methods and to enable a greater conceptual understanding. First, studies use diverse metrics (for example, species richness, evenness and functional and phylogenetic diversity), as well as differing sampling effort, spatial scale and regional species pools. This heterogeneity impedes direct comparison of raw biodiversity values, introducing both statistical noise and interpretive ambiguity in cross-system syntheses. Thus, rescaling isolates the within-study relationship between biodiversity and ecosystem service and functions, enabling comparability across studies despite differences in biodiversity measurements and sampling design, which was critical to identifying general BES and BEF patterns across ecosystems and experimental contexts. This approach also lets us summarize and unpick how systems respond to differences in biodiversity seen within that system, be it observational or experimental, and so understand how ecosystems might be expected to respond.

#### BES and BEF form

To determine the form (shape) of BES and BEF relationships, we applied multiple statistical models to evaluate if relationships were linear or saturating (quadratic or logarithmic). Model selection was based on Akaike weights, identifying the best-fitting relationship for each dataset. We calculated a single coefficient in the case of linear and logarithmic relationships and two coefficients in the case of polynomial relationships. We calculated AIC scores for BES and BEF data fit by linear, quadratic and logarithmic models for each dataset. Using these AIC scores, we calculated Akaike weights for each dataset to determine the relative likelihood that a model is correct compared with the other models considered. We calculated the average Akaike weight across datasets for each ecosystem service and function category for comparison.

#### Saturation types

We assessed saturation points of BES and BEF relationships across ecosystem types. Saturation points were defined as the biodiversity value at which the slope of the relationship reached zero for quadratic models with negative, significant curvature or the value at which the slope declined to 10% of its initial value for logarithmic models; linear models were treated as non-saturating. Analyses were done on non-standardized data and restricted to taxonomic, count-based species richness data.

#### Variation in biodiversity metrics and relationship forms

To assess how methodological choices vary across ecosystem service and ecosystem function categories, we analysed both the distribution of biodiversity metric classes and the form of fitted BEF and BES relationships. Biodiversity metrics were grouped into four classes (taxonomic, functional, phylogenetic and other). Our literature search was designed to capture broad BEF and BES relationships rather than systematically sample biodiversity metric types; as such, these analyses should be interpreted as descriptive patterns within the compiled literature rather than unbiased estimates of metric usage across the field.

First, we tested whether the distribution of biodiversity metric classes differed among ecosystem service and ecosystem function categories using a chi-squared test of independence, based on the contingency table shown in Extended Data Fig. 1. Because several cells had low expected frequencies, significance was assessed using Monte Carlo simulation (9,999 permutations). Effect size was quantified using Cramér’s *V*, and standardized residuals were used to identify over- and underrepresented combinations.

We then examined whether best-fitting relationship form differed among biodiversity metric classes. For each observation, the best-fitting model (linear, logarithmic or quadratic) was selected on the basis of the highest Akaike weight. A contingency table of biodiversity metric class versus model type was analysed using a chi-squared test, and standardized residuals were used to identify deviations from expected frequencies.

### Effect size

To determine differences in biodiversity effects among ecosystem services and functions, we calculated effect size as the response of services and functions to increasing biodiversity for each dataset. To calculate effect size, we used simple correlation coefficients (*r*) between BES relationships, which were normalized using Fisher’s *z*-algorithm^34^ (Zr) as

1 $$\mathrm{Zr}=0.5\quad\mathrm{ln}\,\times \,(1+r)/(1-r).$$

Zr normalizes correlation coefficients for statistical comparison and allows us to compare the average strength of BES and BEF relationships among categories. We use the metafor package (v. 4.2-0) to carry out Cochran’s *Q* test and construct a funnel plot to test for residual heterogeneity.

### Ecosystem type, study type and spatial grain

We used a reduced linear mixed-effects model to determine the effects of ecosystem service and function category, ecosystem type, study type and spatial grain on BES and BEF relationships. We explicitly included interactions between biodiversity and each of these factors to test whether the strength or direction of BES and BEF relationships depended on ecosystem function or service type, biome, study design or spatial scale. In additiont, three-way interactions between biodiversity, ecosystem service type, and each factor were included to capture more complex, function-specific dependencies. We used the dataset as a random effect to control for differences across studies and this was forced to be in every model during model selection. AIC-based model selection was then used to retain the most informative effects while minimizing overfitting. We did not run model averaging, as no other model was well supported by our data (ΔAICc <4). Our final model was: ecosystem service value ≈ biodiversity value + ecosystem type + ecosystem service category + study type + spatial grain + (1|dataset) + biodiversity value:ecosystem type + biodiversity value:ecosystem service category + biodiversity value:study type + biodiversity value:spatial grain + biodiversity value:ecosystem type:ecosystem service category + biodiversity value:ecosystem service category:study type + biodiversity value:ecosystem service category:spatial grain. Models were run using lme4 and lmerTest packages, and model selection was performed using MuMIn in R^62–64^.

### Forecast future changes to ecosystem service

To model-predicted future changes to biodiversity, we used projected BII values from the PREDICTS project^56,65^. BII is the average abundance across a large and functionally diverse set of species relative to their reference populations but does not consider climate change impact^52^. Specifically, we obtained rasterized (spatial resolution 0.25 degrees) predicted BII values (and country-level averaged BII values) for each of the five SSP scenarios described by the IPCC^66,67^. These pathways include sustainable development (SSP1), middle of the road (SSP2), regional rivalry (SSP3), inequality (SSP4) and fossil fuel development (SSP5) and were taken for the following years: 1970, 2020, 2030, 2040 and 2050. We chose these pathways as they represent the most realistic alternative socioeconomic development trajectories under future socioeconomic development^66^.

There is a fundamental disconnect between how biodiversity is measured and how potential future changes to biodiversity are forecast. The latter typically comprises unitless, bounded indices (such as BII), whereas the former comprises a variety of measurements, including primary production, species abundance and percentage cove^7,68^. These metrics vary substantially in terms of their units of measurement, spatial scale, and the range of plausible data values. To address this issue, matching the range of values of BII data (bounded from 0–1.18) and BES data, we used four different scaling procedures, so biodiversity values correspond to BII values of 0–1.18, 0.3–1.18, 0–0.6 and 0.3–0.6, respectively. These ranges were chosen to capture the full spectrum of possible values, exclude extreme low outliers, focus on low-to-moderate biodiversity or highlight the mid-range where most sites cluster. Using multiple overlapping scales ensures that observed trends in BES relationships are robust and not an artefact of the chosen scaling procedure, allowing clear visualization of qualitative patterns. This was achieved by applying a scaling function as follows:

2 $$\mathrm{scaled}\left(x\right)=x/{x}_{\max }\times \left(u{\rm{\mbox{--}}}l\right)+1,$$

where *x* is an unscaled biodiversity value, *u* is the upper bound of the scaled data (1, 0.6 or 0.6), and l is the lower bound of the scaled data (0.3, 0 or 0.3). While this does not create exact quantitative equivalence between metrics, it ensures that trends in BES relationships can be visualized unambiguously and compared across different types of biodiversity data, allowing qualitative patterns to emerge despite the diversity of original measurements.

All non-terrestrial biodiversity and ecosystem services data were excluded, as PREDICTS contains BII values for terrestrial ecosystems only. To control for baseline differences in ES, BII data were subset by ecoregions to allow ecosystem services predictions to be made separately for each ecoregion^69^. Within our database, each BES data point was also assigned an ecoregion. Predictions for future ecosystem services values were obtained by inputting scaled BII values into BES forecast models using a posterior sampling method. Thus, projected changes represent changes in the measured ecosystem service response variable used in each BES relationship, rather than independent predictions of the underlying biological components contributing to that service. Our projections assume that changes in BII are independent of the functional importance of the species affected. Consequently, projected changes in ecosystem services reflect aggregate changes in biodiversity rather than species-specific compositional turnover or differential contributions of individual species to ecosystem functioning. As the observed BES relationships are underlain by uncertainty owing to variation in experimental approaches, spatiotemporal scales and sample sizes, we used a Monte Carlo uncertainty approach to propagate this uncertainty forward. Specifically, one hundred predictions of future ecosystem services were drawn from two normal distributions (one centred around the mean BES coefficient and the other centred around the mean intercept, both with standard error equivalent to the mean standard error of BES coefficients and intercepts respectively) for each unique combination of ES, ecoregion, year and SSP. In the case of quadratic relationships, three normal distributions were used for posterior sampling owing to the existence of a quadratic BES coefficient in addition to the linear BES coefficient term.

The predictions obtained by this procedure are, at this stage, relative and unitless. This makes inferring the scale of the predicted change in ecosystem services challenging. Consequently, we scaled predicted ecosystem services values to represent absolute values with known units as follows:

3 $$\mathrm{Scaled}\left({p}_{x}\right)={p}_{x}-p{f}_{\min }/p{f}_{\max }\times \left({f}_{\max }-{f}_{\min }\right)+{f}_{\min },$$

where *p* is the predicted ecosystem service at a BII value *x*, and *f*_min_ and *f*_max_ are the raw, absolute minimum and maximum ecosystem service values. As ecosystem service values do not have the same units, *f*_min_ and *f*_max_ were obtained from the ecosystem service type that was most prevalent for each given ecosystem service.

To visualize ecosystem service forecasts, mean predicted ecosystem service values were generated for each unique combination of ecosystem service, ecoregion, year and SSP, and mean forecasts were plotted using the R package sf.

To visualize spatial uncertainty in predicted ecosystem services, Monte Carlo simulations for each combination of ecosystem service type, ecoregion, year and climate scenario were compiled into raster stacks. From these stacks, we calculated 95% credible interval (a Bayesian measure of uncertainty around estimated values) at each grid cell as the 2.5th and 97.5th percentiles of the posterior predictive distribution. These intervals were mapped alongside mean predicted ecosystem service values to produce spatially explicit projections with associated uncertainty. This approach allows the comparison of both expected values and uncertainty across ecoregions and climate scenarios, providing a robust and interpretable representation of projected BEF and BES dynamics.

#### Country-level predictions, population change and HDI

We examined the potential for correlation between future population change or HDI against forecasted BES relationships. HDI is a composite indicator based on life expectancy, education and income, with high values reflecting greater socioeconomic development and typically greater capacity to buffer environmental change, whereas low values indicate lower development and higher reliance on local ecosystem services. We used the approach outlined in the section above (‘Forecast future changes to ecosystem service’ section) to forecast country-level relative changes in ‘food and feed’ provisioning in the years 1970 and 2050 under SSP2 and SSP5 pathways. For these forecasts, country-level average BII values were used. The percentage change in population forecasted to occur between 1970 and 2050 in each country was obtained from the United Nations Department of Economic and Social Affairs Population Division^70^. HDI data, which is a summary of three dimensions of human health (a long and healthy life, being knowledgeable and having a decent standard of living) are from 2023, the most recent year available^57^. Note that any territory for which BII data were not accessible was excluded. Projected regional differences in ecosystem service outcomes emerge from the interaction between scenario-driven changes in biodiversity (via BII under SSPs), socioeconomic context (captured by HDI and population trajectories) and uncertainty in the underlying BES relationships, which is propagated through all projections.

We assessed the association between projected population growth, HDI and the relative change in food and feed provisioning between 1970 and 2050. We used a spatial autoregressive (SAR) model to account for spatial autocorrelation between regions in both models (percentage population growth, Moran’s *I* = 0.457, *P* < 0.0001; HDI, Moran’s *I* = 0.449, *P* < 0.0001). We use the R package spatialreg to construct the SAR model^71^.

#### Assumptions and limitations of predictive modelling

This predictive framework does not explicitly represent key mediating factors known to influence pest regulation outcomes, including landscape configuration, crop type, agricultural management practices or climate-driven changes in crop productivity. The model therefore represents biodiversity-only effects on a biotic regulatory pathway rather than a full agricultural yield or food system model.

### Reporting summary

Further information on research design is available in the Nature Portfolio Reporting Summary linked to this article.

## Supplementary information

Supplementary Information Supplementary Figs. 1–3 and Tables 1–5.

Reporting Summary

Peer Review File

Supplementary Fig. 1 Supplementary Fig. 1. Comparison of linear, log and quadratic model fits describing BES relationships across ecosystem function categories. Count (*n*) values for each category are shown in Fig. 2b. Points show mean *r*^2^ values, and error bars indicate ±1 s.e. across studies. Response variables that are classified by the IPBES as ecosystem functions are marked with an asterisk (*)^11^, while ecosystem services are not.

## Source data

Source Data Fig. 1 GPS, spatial grain experimental and observation data from Fig. 1, unprocessed.

Source Data Fig. 2 AIC weight data, one row per dataset/label in our database.

Source Data Fig. 3 Effect size data including standard error and confidence intervals, one row per dataset/label in our database.

Source Data Fig. 4 Country level predictions of food and feed alongside HDI and population data.

Source Data Extended Data Fig.1 Data on the distribution of observations across biodiversity metric classes versus ecosystem services and functions and biodiversity metric classes versus reported relationship type.

Source Data Extended Data Fig. 2 Saturation point data for marine, freshwater and terrestrial ecosystems.

Source Data Extended Data Fig. 3 Effect size data split by ecosystem type.

Source Data Extended Data Fig. 4 Effect size data averages across ecosystem function/service categories and study type.

## Figures and Tables

**Extended Data Fig. 1:**
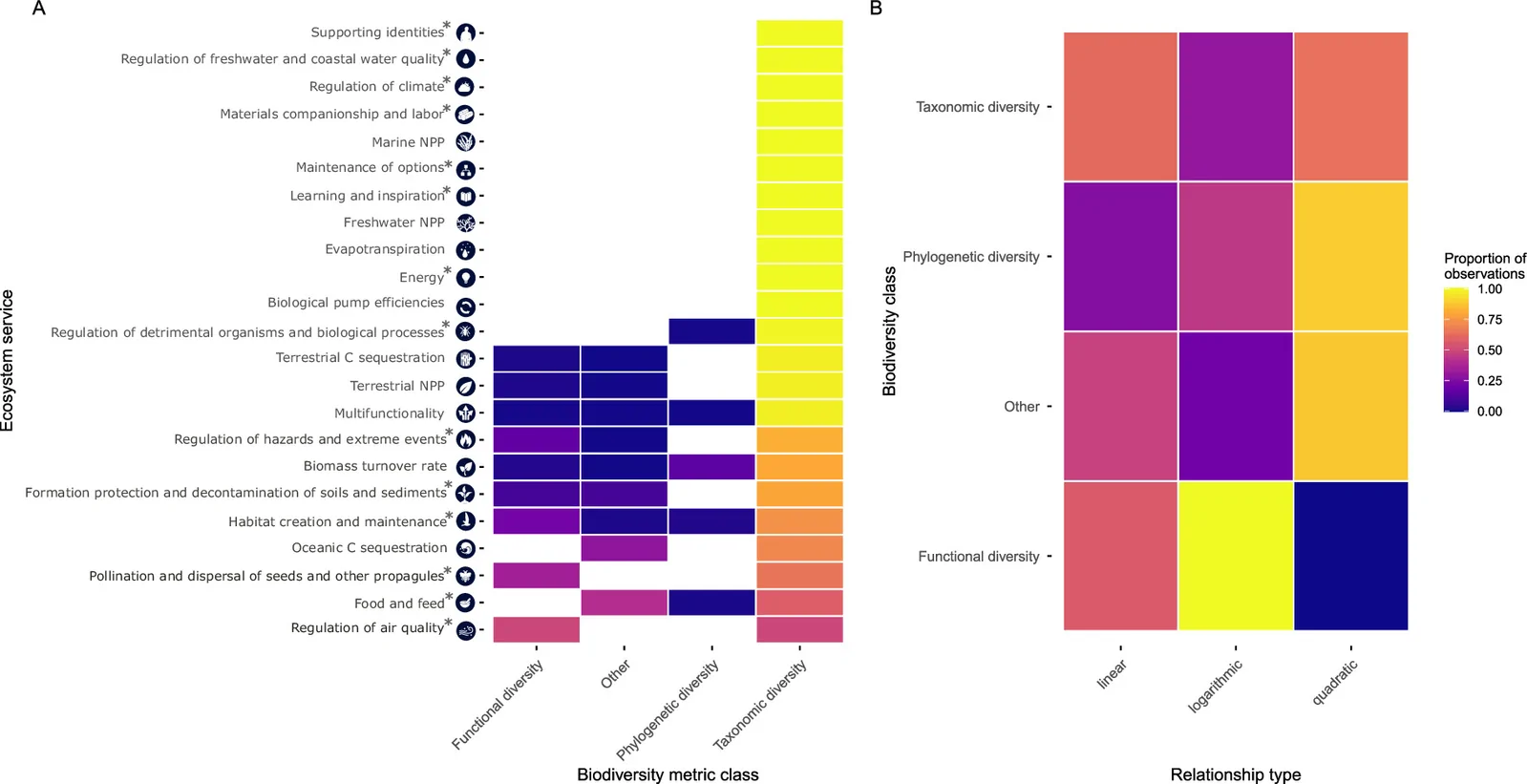
Patterns of biodiversity metrics and reported relationships across ecosystem services and functions. Heatmaps show the distribution of observations across (**a**) biodiversity metric classes versus ecosystem services and functions, and (**b**) biodiversity metric classes versus reported relationship types. Colour intensity indicates the proportion of observations within each category, with yellow and orange representing higher proportions. The distribution of biodiversity metrics differed significantly among ecosystem service categories (χ^2^ = 78.385, Monte Carlo p < 0.001; Cramér’s V = 0.34), with taxonomic diversity metrics common in our database. Biodiversity-ecosystem service and biodiversity ecosystem-function forms also varied among biodiversity metrics (χ^2^ = 23.17, df = 6, p < 0.001), with functional and phylogenetic diversity metrics showing an over-representation of saturating relationships, whereas taxonomic relationships were equally represented across model types. Response variables marked with an asterisk (*) are classified as ecosystem functions (EF), all other response variables are ecosystem services (ES). Source data

**Extended Data Fig. 2:**
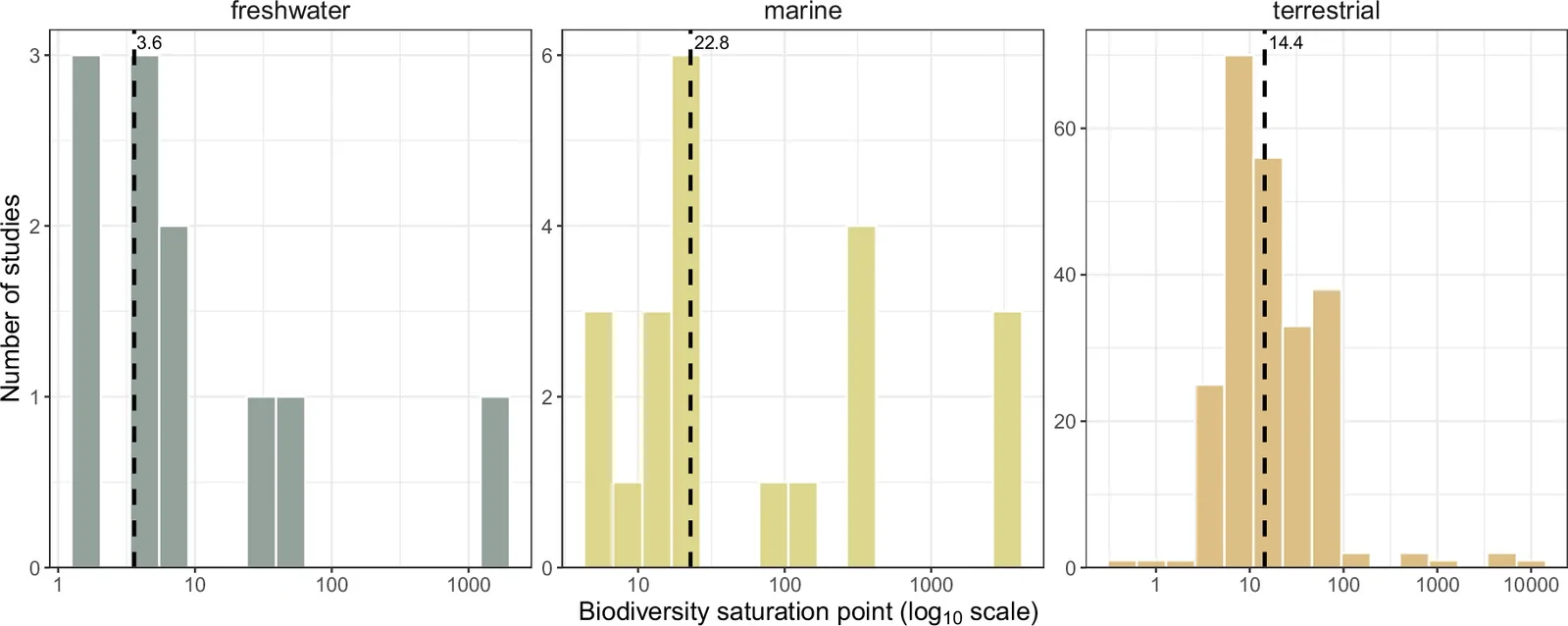
Biodiversity and ecosystem-service and biodiversity ecosystem function saturation points across ecosystem types. Biodiversity data are untransformed species richness (count data) only. Saturation points occur at lower median species richness in freshwater (3.6 ± 1.99) and terrestrial (14.4 ± 9) systems than in marine ecosystems (22.8 ± 16.5), where higher median values are observed. Uncertainty is expressed as median absolute deviation (MAD). Data are from datasets best predicted by quadratic and logarithmic models, as indicated by Akaike Weight values. Saturation for quadratic models was calculated when the slope reached zero, and for logarithmic models, when the slope was 10% of the initial value. The horizontal dashed line shows the median value for each ecosystem type. Large saturation values may be due to the large spatial scale the data were collected at or due to the diverse fauna at lower levels of the food web (*for example*, meiofauna in marine ecosystems). Source data

**Extended Data Fig. 3:**
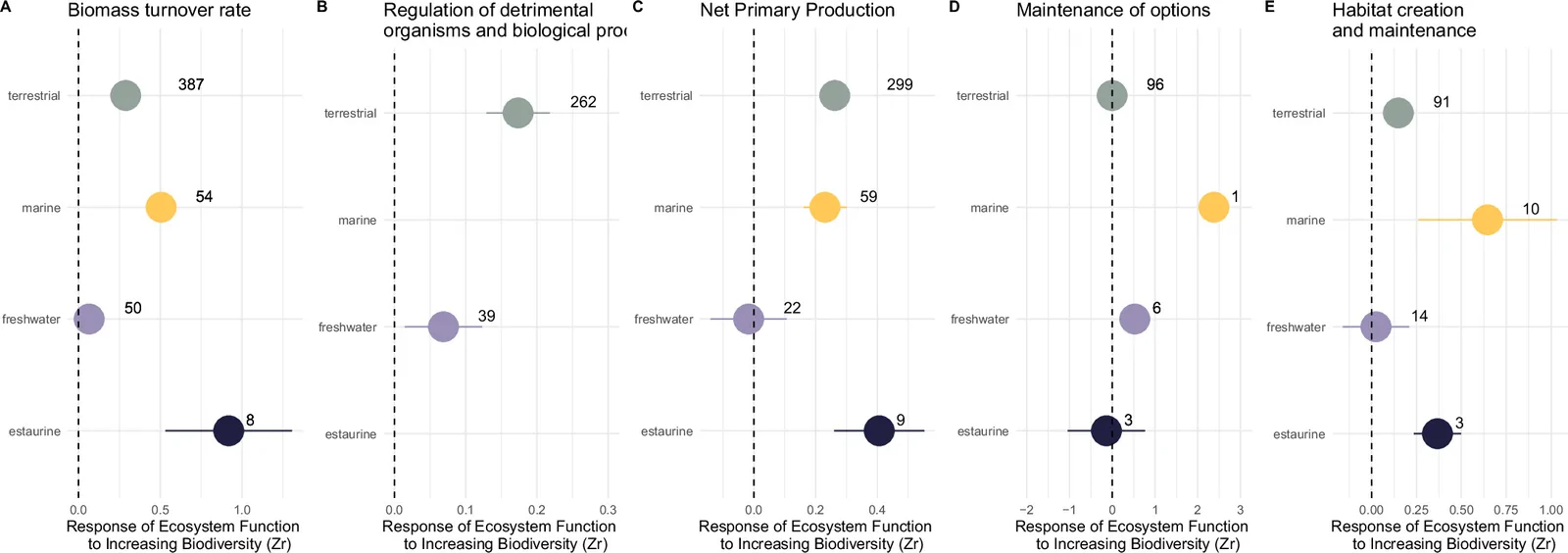
Biodiversity and ecosystem service effect sizes vary across ecosystem types. Data are shown for our database’s five most common ecosystem service categories (panels **a**–**e**). Effect size scores (measured with Fisher’s Z-score; Zr) are averaged (±1 SE) across datasets within each ecosystem service category and ecosystem type, where a high effect size value indicates a stronger effect of biodiversity on ecosystem service compared to a lower value. Dataset n values are shown beside the average effect size value. Overall, our database has high dataset n values for terrestrial studies and low n values for estuarine studies, and this was consistent across all ecosystem service categories. Datasets from marine and freshwater ecosystems were not as common as those from terrestrial studies, and for some ecosystem services (regulation of detrimental organisms and biological processes, maintenance of options), data from these ecosystem types were scarce or missing. Source data

**Extended Data Fig. 4:**
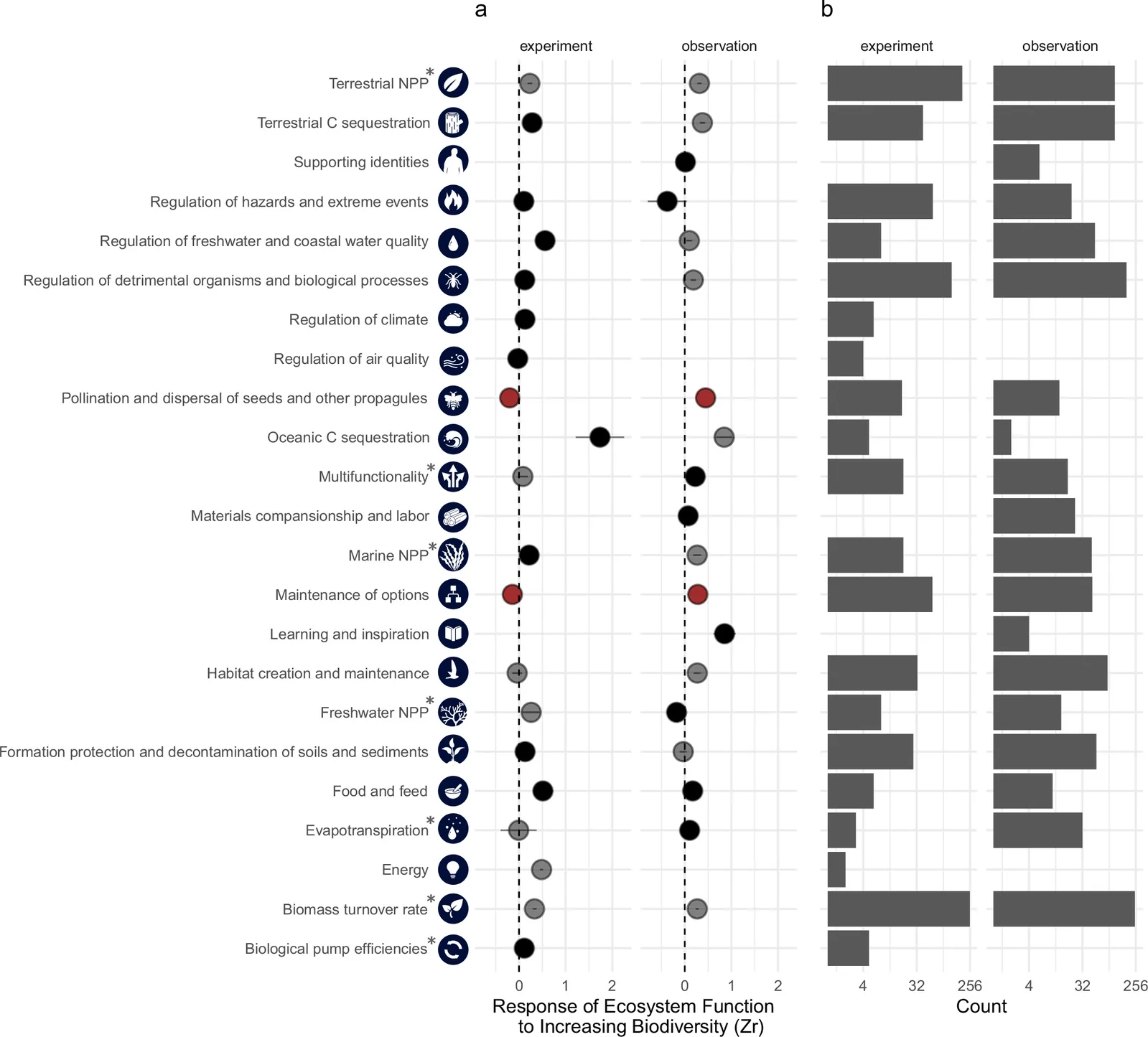
Comparison of biodiversity responses across ecosystem service categories, as effect size (Fisher’s Z-score; Zr; Panel a) and the number of studies used to calculate effect size (Panel **b**). High effect size values indicate a stronger effect of biodiversity on ecosystem service compared to values. Data are split by study type. The effect sizes which are different (positive vs negative) between study types, are highlighted Response variables marked with an asterisk (*) are classified as ecosystem functions (EF), all other response variables are ecosystem services (ES)^11^. Source data

## Data Availability

The datasets generated during and analysed during the current study are available via Zenodo at https://doi.org/10.5281/zenodo.21456540 (ref. ^72^). Source data are provided with this paper.

## Code availability

The code generated to produce models in this study is available via Zenodo at https://doi.org/10.5281/zenodo.21456540 (ref. ^72^).
