# Supplementary material for: Biodiversity safeguards ecosystem services and functions worldwide
Source: Nat Ecol Evol. 2026 Oct 6;10(11):2037–48. doi: 10.1038/s41559-026-03200-4 (PMC13736519; doi:10.1038/s41559-026-03200-4)
Supplement: Supplementary file 1 [file 41559_2026_3200_MOESM1_ESM.pdf]

nature ecology & evolution

Supplementary information https://doi.org/10.1038/s41559-026-03200-4

Biodiversity safeguards ecosystem services and functions worldwide

In the format provided by the authors and unedited

Supplementary figures

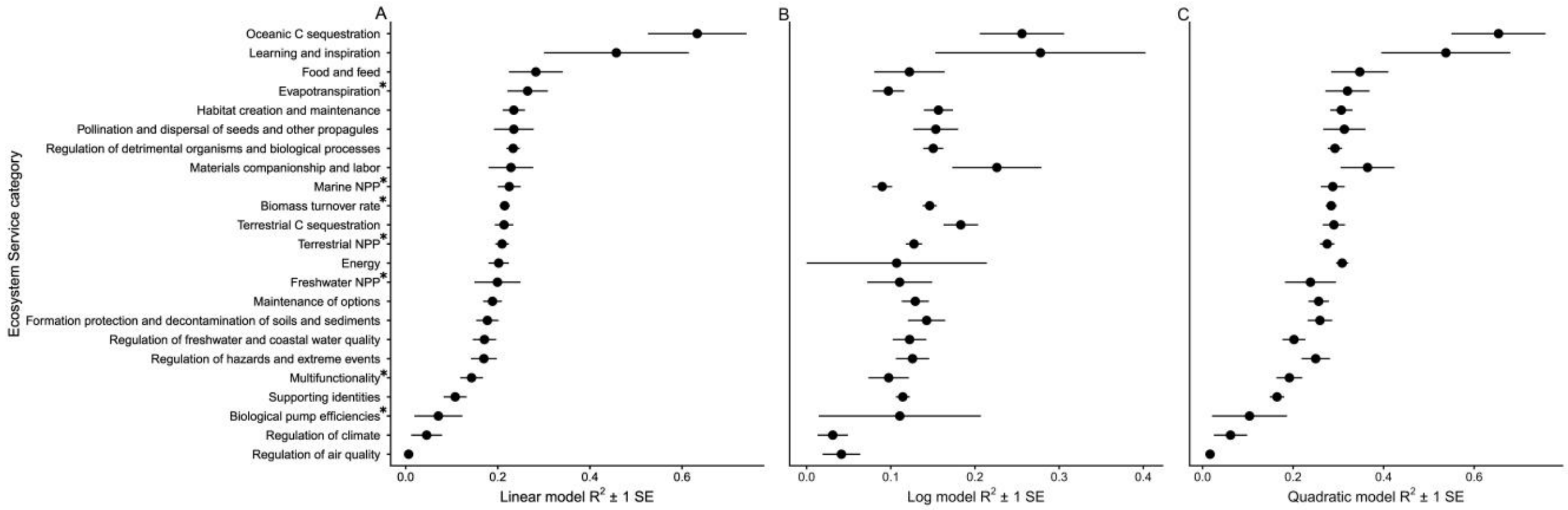

Supplementary Figure 1. Comparison of linear, log, and quadratic model fits describing biodiversity- ecosystem service relationships across ecosystem function categories. Count (n) values for each category are shown in Fig 2B. Points show mean R^2 values, and error bars indicate ±1 SE across studies. Response variables that are classified by the IPBES as ecosystem functions (EF) are marked with an asterisk (*)[11], while ecosystem services (ES) are not.

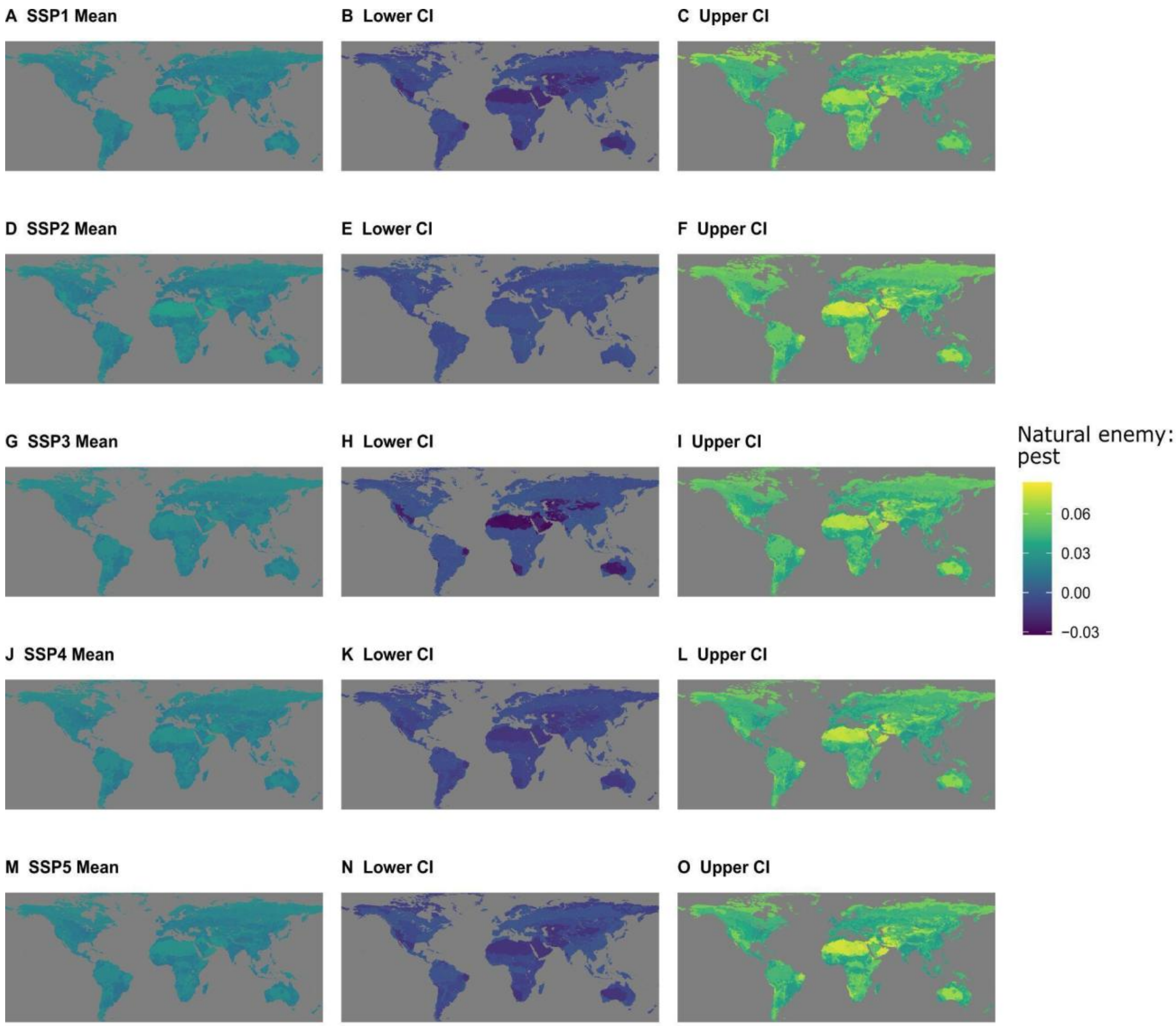

Supplementary Figure 2. Global projections of natural enemy–pest ratios under future socio-economic scenarios. Spatial patterns of the natural enemy to pest ratio are shown for five Shared Socioeconomic Pathways (SSP1-SSP5) in 2050. Rows correspond to scenarios, and columns show the mean projection and associated 95% credibility intervals (lower and upper bounds). Colours indicate the magnitude of the ratio, with a common scale applied across panels to enable comparison among scenarios and uncertainty estimates. Low natural enemy-pest ratios indicate reduced biological pest control capacity and higher potential pest pressure. Projections show spatially heterogeneous biocontrol potential, with scenario-dependent shifts in magnitude and regionally variable uncertainty, generally indicating lower ratios under higher-pressure SSPs.

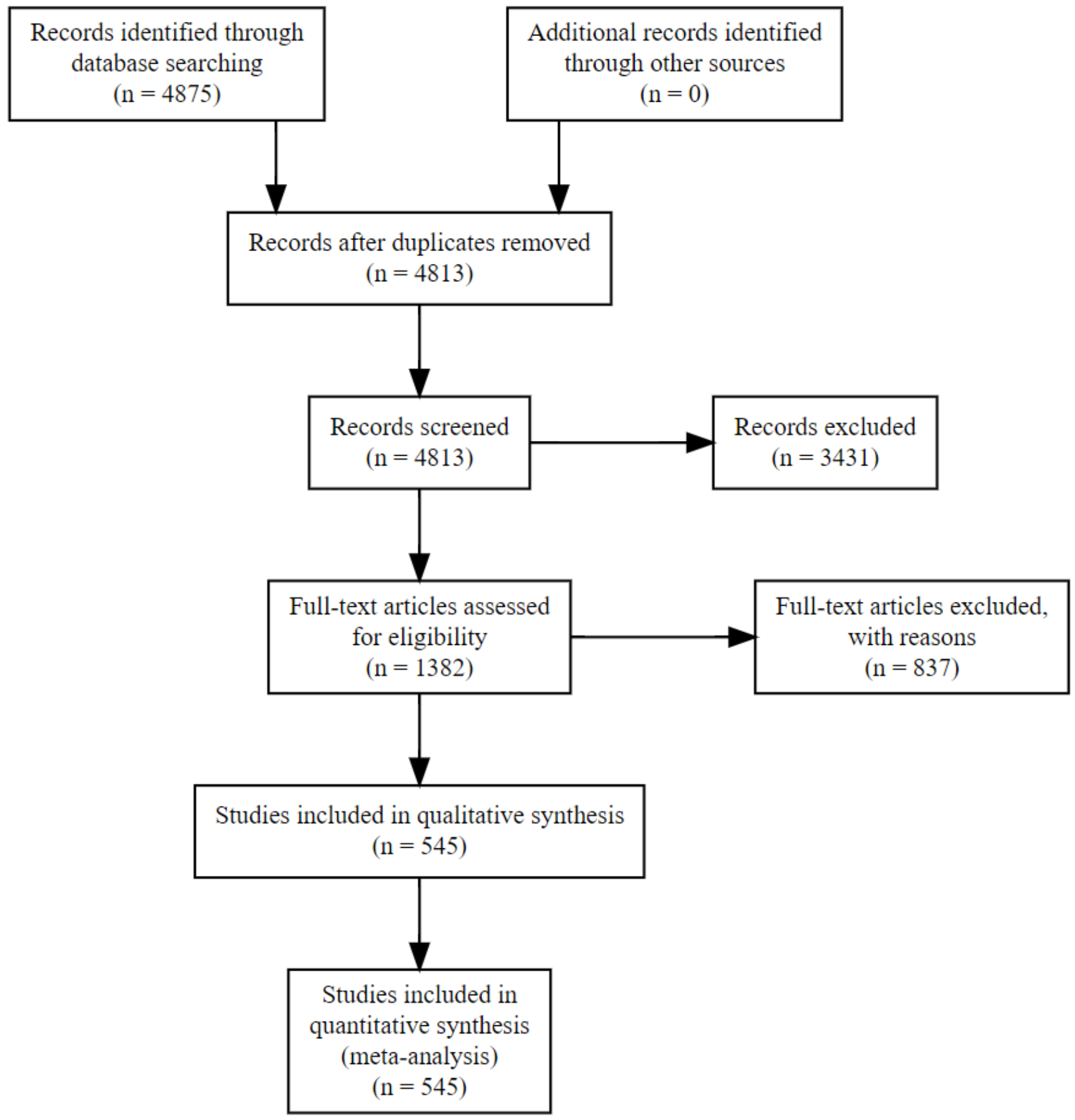

Supplementary Figure 3. Summary of the study selection process for our stratified meta-analysis. Papers were excluded if they were not relevant to our project, were based on simulated data, were review articles, or could not be accessed. At the full-text stage, studies were further excluded if no biodiversity-ecosystem dataset was available or if the ecosystem service or biodiversity metrics were unclear.

Supplementary tables

Supplementary Table 1. Linear Mixed-Effects Model (*lmer*) summary describing the relationship between biodiversity and ecosystem service with interactions with ecosystem service category, ecosystem type, study type (experiment or observational), and spatial grain with a random effect of the dataset.

	Estimate (β)	Std. Error	df	t value	Pr(>|t|)
(Intercept)	0.48	0.81	2729	0.59	0.554
Biodiversity value	11.86	0.50	165802	23.56	0.000
Biome general freshwater	0.39	0.25	2299	1.57	0.116
Biome general marine	1.02	0.25	2323	4.08	0.000
Biome general terrestrial	0.54	0.23	2422	2.32	0.020
Ecosystem service metric Biomass turnover rate	0.50	0.78	2756	0.64	0.523
Ecosystem service metric Energy	0.57	0.94	2644	0.60	0.546
Ecosystem service metric Evapotranspiration	0.48	0.79	2713	0.61	0.539
Ecosystem service metric Food and feed	–0.22	0.82	2724	–0.27	0.789
Ecosystem service metric Formation protection and decontamination of soils and sediments	0.34	0.78	2755	0.43	0.664
Ecosystem service metric Habitat creation and maintenance	0.45	0.78	2751	0.58	0.562
Ecosystem service metric Learning and inspiration	–0.27	0.88	2867	–0.31	0.755
Ecosystem service metric Maintenance of options	0.94	0.78	2747	1.21	0.227

Ecosystem service metric Materials companionship and labour	0.06	0.80	2860	0.07	0.942
Ecosystem service metric Multifunctionality	0.80	0.79	2726	1.02	0.309
Ecosystem service metric NPP	0.32	0.78	2755	0.41	0.681
Ecosystem service metric Oceanic C sequestration	–1.53	0.90	3166	–1.69	0.090
Ecosystem service metric Pollination and dispersal of seeds and other propagules	0.20	0.80	2739	0.25	0.806
Ecosystem service metric Regulation of air quality	1.11	0.85	2529	1.31	0.189
Ecosystem service metric Regulation of climate	1.38	0.83	2625	1.67	0.094
Ecosystem service metric Regulation of detrimental organisms and biological processes	0.54	0.78	2755	0.70	0.485
Ecosystem service metric Regulation of freshwater and coastal water quality	–0.13	0.78	2746	–0.17	0.868
Ecosystem service metric Regulation of hazards and extreme events	–0.02	0.78	2936	–0.02	0.984
Ecosystem service metric Terrestrial C sequestration	0.55	0.78	2756	0.71	0.478
Experiment or Observation observation	-0.07	0.04	1913	-1.76	0.079
Spatial grain	–0.06	0.01	1787	–4.14	0.000
Biodiversity value: Biome general freshwater	0.34	0.13	53780	2.59	0.010
Biodiversity value: Biome general marine	–0.25	0.10	48610	–2.42	0.016
Biodiversity value: Biome general terrestrial	–0.02	0.10	46262	–0.23	0.819
Biodiversity value: Ecosystem service metric Biomass turnover rate	–17.63	2.54	41745	–6.94	0.000
Biodiversity value: Ecosystem service metric Energy	0.28	0.41	140984	0.69	0.490

Biodiversity value: Ecosystem service metric Evapotranspiration	–12.80	1.69	90650	-7.58	0.000
Biodiversity value: Ecosystem service metric Food and feed	248.33	809.04	35983	0.31	0.759
Biodiversity value: Ecosystem service metric Formation protection and decontamination of soils and sediments	14.42	24.12	28303	0.60	0.550
Biodiversity value: Ecosystem service metric Habitat creation and maintenance	–11.72	0.52	160912	–22.73	0.000
Biodiversity value: Ecosystem service metric Learning and inspiration	0.44	0.30	169108	1.46	0.145
Biodiversity value: Ecosystem service metric Maintenance of options	–11.88	0.49	168065	–24.08	0.000
Biodiversity value: Ecosystem service metric Materials companionship and labour	–0.13	0.28	168311	–0.45	0.654
Biodiversity value: Ecosystem service metric Multifunctionality	–338.03	43.08	52892	–7.85	0.000
Biodiversity value: Ecosystem service metric NPP	–11.74	0.49	168070	–23.80	0.000
Biodiversity value: Ecosystem service metric Oceanic C sequestration	0.96	0.35	169124	2.75	0.006
Biodiversity value: Ecosystem service metric Pollination and dispersal of seeds and other propagules	10.96	22.28	19308	0.49	0.623
Biodiversity value: Ecosystem service metric Regulation of air quality	–0.19	0.27	168984	–0.70	0.487
Biodiversity value: Ecosystem service metric Regulation of climate	162.79	1960.73	145768	0.08	0.934t
Biodiversity value: Ecosystem service metric Regulation of detrimental organisms and biological processes	–11.81	0.49	168082	–23.96	0.000
Biodiversity value: Ecosystem service metric Regulation of freshwater and coastal water quality	–0.22	0.26	167739	–0.85	0.395
Biodiversity value: Ecosystem service metric Regulation of hazards and extreme events	–26585.66	6109.11	15	–4.35	0.001

Biodiversity value: Ecosystem service metric Terrestrial C sequestration	-0.14	0.27	168952	–0.52	0.601
Biodiversity value: Experiment or Observation observation	0.03	0.01	164839	2.81	0.005
Biodiversity value: Spatial grain	210.26	7.46	167511	28.17	0.000
Biodiversity value: Biome general freshwater: Ecosystem service metric Biomass turnover rate	–0.64	0.16	51687	–3.89	0.000
Biodiversity value: Biome general marine: Ecosystem service metric Biomass turnover rate	0.36	0.14	48699	2.52	0.012
Biodiversity value: Biome general terrestrial: Ecosystem service metric Biomass turnover rate	–0.12	0.14	47344	–0.83	0.404
Biodiversity value: Biome general marine: Ecosystem service metric Energy	0.25	0.33	54020	0.74	0.459
Biodiversity value: Biome general freshwater: Ecosystem service metric Habitat creation and maintenance	–0.13	0.22	34116	–0.59	0.557
Biodiversity value: Biome general marine: Ecosystem service metric Habitat creation and maintenance	0.58	0.23	73773	2.53	0.011
Biodiversity value: Biome general terrestrial: Ecosystem service metric Habitat creation and maintenance	–0.11	0.15	38823	–0.72	0.472
Biodiversity value: Biome general freshwater: Ecosystem service metric Maintenance of options	0.40	0.11	80453	3.81	0.000
Biodiversity value: Biome general marine: Ecosystem service metric Maintenance of options	1.24	0.06	143477	20.55	0.000
Biodiversity value: Biome general freshwater: Ecosystem service metric NPP	–0.60	0.09	69883	–6.42	0.000
Biodiversity value: Biome general freshwater: Ecosystem service metric Regulation of detrimental organisms and biological processes	–0.34	0.09	68828	–3.71	0.000

Biodiversity value: Ecosystem service metric Biomass turnover rate: Experiment or Observation observation	–0.03	0.02	135885	–1.99	0.046
Biodiversity value: Ecosystem service metric Evapotranspiration: Experiment or Observation observation	0.05	0.15	121722	0.35	0.728
Biodiversity value: Ecosystem service metric Food and feed: Experiment or Observation observation	–0.24	0.17	26638	–1.40	0.161
Biodiversity value: Ecosystem service metric Formation protection and decontamination of soils and sediments: Experiment or Observation observation	–0.04	0.04	42188	–0.95	0.343
Biodiversity value: Ecosystem service metric Habitat creation and maintenance: Experiment or Observation observation	0.10	0.04	65882	2.58	0.010
Biodiversity value: Ecosystem service metric Maintenance of options: Experiment or Observation observation	0.13	0.02	138281	5.82	0.000
Biodiversity value: Ecosystem service metric Multifunctionality: Experiment or Observation observation	0.48	0.08	69308	5.89	0.000
Biodiversity value: Ecosystem service metric NPP: Experiment or Observation observation	0.19	0.01	153216	13.06	0.000
Biodiversity value: Ecosystem service metric Pollination and dispersal of seeds and other propagules: Experiment or Observation observation	0.37	0.09	54450	4.14	0.000
Biodiversity value: Ecosystem service metric Regulation of detrimental organisms and biological processes: Experiment or Observation observation	0.12	0.02	125804	6.55	0.000
Biodiversity value: Ecosystem service metric Regulation of hazards and extreme events: Experiment or Observation observation	–0.13	0.06	79552	–2.14	0.032
Biodiversity value: Ecosystem service metric Biomass turnover rate: Spatial grain	–319.57	45.37	41030	–7.04	0.000
Biodiversity value: Ecosystem service metric Evapotranspiration: Spatial grain	–234.20	29.94	88339	–7.82	0.000

Biodiversity value: Ecosystem service metric Food and feed: Spatial grain	4465.40	14577.67	35982	0.31	0.759
Biodiversity value: Ecosystem service metric Formation protection and decontamination of soils and sediments: Spatial grain	262.17	434.49	28301	0.60	0.546
Biodiversity value: Ecosystem service metric Habitat creation and maintenance: Spatial grain	–210.25	7.46	167511	–28.17	0.000
Biodiversity value: Ecosystem service metric Maintenance of options: Spatial grain	–206.98	7.46	167503	–27.73	0.000
Biodiversity value: Ecosystem service metric Multifunctionality: Spatial grain	–6093.07	776.30	52885	–7.85	0.000
Biodiversity value: Ecosystem service metric NPP: Spatial grain	–210.10	7.46	167511	–28.15	0.000
Biodiversity value: Ecosystem service metric Pollination and dispersal of seeds and other propagules: Spatial grain	202.15	401.35	19286	0.50	0.614
Biodiversity value: Ecosystem service metric Regulation of climate: Spatial grain	2934.71	35323.72	145769	0.08	0.934
Biodiversity value: Ecosystem service metric Regulation of detrimental organisms and biological processes: Spatial grain	–210.27	7.46	167511	–28.17	0.000
Biodiversity value: Ecosystem service metric Regulation of hazards and extreme events: Spatial grain	–478947.01	110057.63	15	–4.35	0.001

Supplementary Table 2. Spatial autoregressive model (SAR) of relative change in food and feed provision from 1970 to 2050, population change from 1970 to 2050, and SSP scenario. Estimates are reported as coefficients (± SE), with SSP2 as the reference category. Population growth had a significant negative effect, and SSP5 predicted significantly lower values than SSP2. The spatial lag parameter was strong (rho = 0.596, $p < 0.001$), confirming spatial dependence.

	Estimate	SE	z	p-value
Intercept	0.413	0.048	8.55	7.75×10^{-18}
Population change	–0.0088	0.0021	–4.28	1.85×10^{-5}
SSP5 (vs SSP2)	–0.0488	0.0105	–4.65	3.34×10^{-6}

Spatial lag parameter: $\rho = 0.596$ (SE = 0.050, $p < 2.22 \times 10^{-16}$)

Model fit: AIC = –540.7, log-likelihood = 275.3, $\sigma = 0.088$.

Residual autocorrelation not detected (LM test p = 0.99).

Supplementary Table 3. Spatial autoregressive model (SAR) of relative change in food and feed provision from 1970 to 2050, Human Development Index (HDI) value, and SSP scenario. Estimates are reported as coefficients (± SE), with SSP2 as the reference category. HDI value had a significant positive effect on food and feed provision, and SSP5 predicted significantly lower values than SSP2.

	Estimate	SE	z	p-value
Intercept	0.252	0.041	6.31	8.96×10^{-10}
HDI value	0.176	0.038	4.59	4.41×10^{-6}
SSP5 (vs SSP2)	–0.049	0.011	–4.60	4.27×10^{-6}

Spatial lag parameter: $\rho = 0.588$ (SE = 0.050, 2.22×10^{-16})

Model fit: AIC = –532.83, log-likelihood = 271.4, $\sigma = 0.088$.

Residual autocorrelation not detected (LM test $p = 0.51$).

Supplementary Table 4. Study-level distribution of biodiversity-ecosystem service datasets across ecosystem types, study types, and spatial grain categories. Counts represent unique studies (identified by label). Study type distinguishes experimental and observational designs. Spatial grain categories reflect the spatial resolution of measurement (fine ≤10 m^2, medium 10–1000 m^2, coarse >1000 m^2). These data show the uneven sampling across ecosystem type, study type, and spatial grain in our database. NA's are present where spatial grain information was not provided in the study.

Ecosystem type	Study type	Spatial grain category	n
estuarine	observation	fine	11
estuarine	observation	*NA*	9
freshwater	experiment	fine	13
freshwater	experiment	medium	1
freshwater	experiment	*NA*	54
freshwater	observation	coarse	3
freshwater	observation	fine	62
freshwater	observation	*NA*	50
marine	experiment	fine	28
marine	experiment	medium	2
marine	experiment	*NA*	13
marine	observation	coarse	4
marine	observation	fine	25
marine	observation	*NA*	80
terrestrial	experiment	coarse	6
terrestrial	experiment	fine	608
terrestrial	experiment	medium	124
terrestrial	experiment	*NA*	26
terrestrial	observation	coarse	141
terrestrial	observation	fine	343
terrestrial	observation	medium	278
terrestrial	observation	*NA*	72

Supplementary Table 5. List of all journal articles used in this stratified meta-analysis. List is sorted by first author surname.

First Author Surname	Publication Year	DOI/ Title
Adair	2018	10.1111/ele.13145
Akre	2016	10.1002/ecs2.1595
Ali	2018	10.1016/j.foreco.2018.05.015
Ali	2018	10.1016/j.foreco.2018.06.042
Ali	2020	10.1016/j.foreco.2020.117968
Andrade	2016	10.1093/femsec/fiw102
Andrey	2014	10.1002/ece3.1118
Aragón	2011	10.1007/s10980-011-9625-z
Arasa-Gisbert	2018	10.1016/j.foreco.2018.02.023
Arenas	2009	10.3354/meps07950
Arnone	2013	10.1007/s00442-012-2585-4
Arnone	2011	10.1111/j.1365-2745.2011.01813.x
Aros-Mualin	2021	10.3389/fpls.2020.615723
Awasthi	2014	10.1038/ismej.2014.91
Azcárate	2013	10.1016/j.actao.2013.03.011
Badano	2009	10.1016/j.ppees.2009.03.002
Baert	2016	10.1002/ecy.1601
Báez	2008	10.1371/journal.pone.0002332
Bailey	2004	10.1111/j.0906-7590.2004.03631.x
Bakker	2016	10.1093/jpe/rtw111
Baquerizo	2017	10.1111/1365-2435.12924
Bar-Massada	2020	10.1071/WF20015
Barnes	2021	10.1016/j.marenvres.2021.105500
Beirão	2020	10.1111/btp.12860
Bell	2005	10.1038/nature03891
Belote	2014	10.1890/ES13-00266.1
Benavides	2018	10.1016/j.ppees.2018.12.002
Berauer	2019	10.1080/15230430.2019.1618116
Bestion	2021	10.1073/pnas.2019591118
Bestion	2020	10.1111/ele.13444
Bhandari	1905	Diversity and productivity of semi-arid grassland of inner mongolia: Influence of plant functional type and precipitation
Bianchelli	2020	10.1038/s41598-020-76817-5
Boeck	2006	10.1016/j.envexpbot.2006.07.001
Boeck	2007	10.1111/j.1469-8137.2007.02122.x
Borthagaray	2020	10.1111/fwb.13603
Bowker	2021	10.1111/oik.08473
Boyero	2021	10.1038/s41467-021-23930-2
Bracken	2008	10.1073/PNAS.0704103105

Brezzi	2017	10.1093/jpe/rtw038
Cadotte	2013	10.1073/pnas.1301685110
Cadotte	2012	10.2307/23229920
Cai	2016	10.1007/s11284-016-1361-z
Cannicci	2021	10.1073/pnas.2016913118
Cardinale	2007	10.1890/03-0727
Cardinale	2009	10.1890/08-1038.1
Cavanaugh	2014	10.1111/geb.12143
Cermeño	2013	10.3354/meps10443
Cerrano	2009	10.1007/s10531-009-9712-5
Cesarz	2020	10.1101/2020.01.30.927277
Chalcraft	2008	10.1007/s10531-008-9457-6
Chaudhury	2022	10.1016/j.tfp.2022.100296
Chaves	2020	10.1111/jvs.12952
Chen	2021	10.3390/agronomy11112092
Chen	2022	10.1016/j.agrformet.2022.109101
Chen	2018	10.1016/j.soilbio.2018.12.008
Chen	2022	10.1111/gcb.16086
Chi	2019	10.1002/ece3.5857
Clough	2006	10.1016/j.agee.2006.05.028
Connolly	2011	10.1890/10-2270.1
Conradi	2020	10.1111/ele.13481
Constán-Nava	2014	10.1007/s10530-014-0780-4
Cooper	2011	10.1111/j.1468-0459.2011.00426.x
Cowles	2015	10.1111/GCB.13111
Craven	2020	10.1111/geb.13165
Creed	2009	10.1111/j.1600-0706.2008.17212.x
da Silva	2016	10.1111/avsc.12230
de Araújo	2013	10.1007/s10531-013-0474-8
De Boeck	2006	10.1007/s11104-006-9112-5
Delgado-Baquerizo	2016	10.1038/ncomms10541
Deng	2014	10.1016/S2095-3119(13)60590-0
Ding	2021	10.1111/1365-2745.13788
Dolezal	2020	10.1111/oik.07379
Donfack	2021	10.1016/j.foreco.2021.119480
Dorado	2014	10.1111/oik.00983
Drake	2003	10.1098/rspb.2003.2423
Du	2019	10.1111/jvs.12806
Duarte	2020	10.1007/s10452-020-09789-3
Dukes	2003	10.1007/s004420000549
Dyola	1905	10.1002/ecs2.4107
Ebeling	2017	10.1111/oik.04210

Ebeling	2014	10.1371/journal.pone.0106529
Ellis	2009	10.1111/j.1600-0706.2009.17695.x
Fargione	2007	10.1098/rspb.2006.0351
Feng	2020	10.1111/geb.13110
Fergus	2017	10.1111/jvs.12563
Feßel	2015	10.1111/jvs.12326
Flombaum	2008	10.1073/pnas.0704801105
Flombaum	2011	doi.org/10.1890/11-0722.1
Fock	2008	10.1111/j.1466-8238.2008.00435.x
Fornara	2008	10.1111/j.1365-2745.2007.01345.x
Fornoff	2019	10.1098/rspb.2018.2399
Forster	2007	10.3354/meps311191
Frank	2006	10.2307/3545501
Fricke	2022	10.1371/journal.pone.0264881
Galandis	1905	Long-term invasibility patterns in Mediterranean grasslands of varying initial diversities
Gao	2016	10.1111/nph.14287
Garibotti	2018	10.1111/1365-2435.13044
Gebert	2019	10.1111/1365-2656.13074
Genung	2014	10.7717/peerj.288
Ghedini	2021	10.1111/1365-2435.13955
Gladstone-Gallagher	2018	10.1016/j.jembe.2018.01.002
Gontijo	2014	10.1111/een.12152
Gonzalez	2003	10.1046/j.1365-2656.2002.00625.x
Gordijn	2018	10.1016/j.ppees.2018.07.005
Goulnik	2020	10.1016/j.agee.2020.107033
Griffin	2008	10.1111/j.1600-0706.2008.16960.x
Gross	2009	10.1016/j.baae.2009.09.001
Gross	2004	10.1034/j.1600-0706.2000.890301.x
Gu	2022	10.1002/ecy.3842
Guo	2021	doi.org/10.21203/rs.3.rs-244027/v1
Guo	2022	10.1016/j.scitotenv.2022.159367
Gustafsson	2010	10.1111/j.1600-0706.2010.19008.x
Haberl	2004	10.1016/j.agee.2003.07.004
Haberl	2005	10.1016/j.agee.2005.03.009
Haddad	2009	10.1111/j.1461-0248.2009.01356.x
Haedo	2017	10.1016/j.ufug.2017.04.017
Häkkilä	2017	10.1371/journal.pone.0184792
Halme	2008	10.1016/j.ecolind.2008.04.005
Hatanaka	2010	10.1111/j.1466-8238.2010.00591.x
Hatfield	2007	10.1016/j.biocon.2007.06.019
Hautier	2017	10.1038/s41559-017-0395-0

Hawkins	2003	10.1086/345479
Hector	2007	10.1038/nature05947
Hector	2002	10.1126/science.286.5442.1123
Hector	2011	10.1371/journal.pone.0017434
Hellicar	2022	10.1016/j.jnc.2022.126207
Hermoso	2010	10.1890/09-2011.1
Hertzog	2016	10.1371/journal.pone.0148768
Heydari	2020	10.1007/s10661-020-08378-z
Hodapp	2013	10.1007/s10531-013-0609-y
Holmquist	2014	10.1111/rec.12111
Hooper	2004	10.1046/j.1461-0248.2003.00555.x
Hooper	2007	10.1890/04-0922
Hooper	2006	10.2307/2657146
Hopper	1905	The Effects of Plant Composition and Diversity on Ecosystem Processes
Hossain	2022	10.1016/j.scitotenv.2022.154322
Hu	1905	Positive plant diversity-soil stability relationships are mediated through roots in the Songnen Grassland: Chronosequence evidence
Hu	2022	10.1007/s11104-022-05731-8
Hu	2022	10.1016/j.ecolind.2022.108762
Huang	2020	10.1111/oik.07032
Ingty	2021	10.1371/journal.pone.0245221
Isbell	2009	10.1111/j.1461-0248.2009.01299.x
Iturrate-Garcia	2016	10.1002/ece3.2548
Jennings	2005	10.1007/s00442-005-0011-x
Jiang	2007	10.1111/j.0030-1299.2007.15371.x
Jin	2020	10.1016/j.foreco.2020.118746
Joly	2017	10.1111/nph.14452
Joner	2021	10.1111/jvs.13060
Jonsson	2019	10.1038/s41477-018-0346-z
Joshi	2021	10.1016/j.tfp.2021.100147
Jourdan	2020	doi.org/10.1007/s10342-020-01329-w
Jucker	2015	10.1111/1365-2435.12428
Jucker	2014	10.1111/1365-2745.12276
Kahmen	2005	10.1111/j.1365-2435.2005.01001.x
Kapkoti	2016	10.1007/s40009-016-0500-y
Karanika	2007	10.1007/s11104-007-9320-7
Kaspari	2004	10.1007/s00442-004-1607-2
Kaspari	2002	10.1086/303313
Kennedy	2022	10.1029/2022GB007481
Kessler	2014	10.1111/1365-2745.12299
Kirwan	2007	10.1111/j.1365-2745.2007.01225.x

Klanderud	2008	10.1657/1523-0430(07-075)[KLANDERUD]2.0.CO;2
Knapp	2015	10.1371/journal.pone.0123052
Knops	2003	10.1007/s004420000537
Kolding	2015	10.1093/icesjms/fsv225
Kozlov	2022	10.3390/insects13010094
Krämer	2010	10.1002/eco.103
Kreyling	2017	10.1111/ELE.12848
Kudo	2011	10.1002/ece3.9
Kudo	2010	doi.org/10.1657/1938-4246-42.4.438
Kumschick	2009	10.1111/j.1365-2311.2009.01127.x
Kutiel	2022	10.1007/BF00040757
Lama	2020	10.1007/s00442-020-04717-6
Lamothe	2019	10.1111/fwb.13395
Lanari	2021	10.1016/j.marenvres.2021.105519
Lange	2015	10.1038/ncomms7707
Lange	2014	10.1371/journal.pone.0096182
Lanta	2012	10.1007/s00114-012-0922-4
Larentis	2021	10.1007/s10750-021-04756-x
Laws	2018	10.1111/1365-2656.12897
Leimer	2013	10.1002/eco.1464
Leimer	2018	10.1002/eco.1945
Leung	2015	10.1016/j.gecco.2015.08.005
Li	2016	10.1002/ldr.2607
Li	2021	10.3389/fpls.2021.704138
Li	2022	10.1007/s11356-022-23938-1
Li	2020	10.1016/j.foreco.2020.118479
Li	2018	10.1016/j.scitotenv.2018.11.024
Li	2020	10.1016/j.scitotenv.2020.137252
Li	2021	10.1093/jpe/rtab021
Li	2022	10.1111/1365-2745.13900
Li	2020	10.1111/jvs.12970
Liang	2020	10.1186/s40657-020-00212-x
Lin	2012	10.1371/journal.pone.0048244
Liu	2022	10.1002/ece3.8838
Liu	2019	10.1002/ldr.3349
Liu	2022	10.1002/ldr.4259
Liu	2021	10.1016/j.agrformet.2021.108526
Liu	2016	10.1371/journal.pone.0154026
Loreau	2002	10.1038/35083573
Lu	2022	10.1111/1365-2745.13982
Luo	2018	10.1111/1365-2435.13109
Ma	2021	10.1007/s00027-021-00833-4
Ma	2020	10.1007/s10021-020-00543-2

Ma	2021	10.3389/fevo.2021.650001
Ma	2018	10.1016/j.soilbio.2018.03.015
Maceda-Veiga	2010	10.1111/j.1365-2427.2010.02407.x
Maestre	2012	10.1126/science.1215442
Magnago	2015	10.1111/gcb.12937
Maravalhas	2019	10.1111/1365-2656.13111
Márialigeti	2009	10.1007/s10531-009-9586-6
Maron	2021	10.1002/ecm.1497
Masumoto	2021	10.1093/femsec/fiab129
Matevski	2020	10.1016/j.foreco.2020.118775
McBride	2014	10.1093/aobpla/plu057/159343
McGrady-Steed	2002	10.1038/36561
McNaughton	2002	10.1086/283181
McNicol	2017	10.1007/s10021-017-0180-6
McWilliam	2018	10.1016/j.cub.2018.09.025
Means	2016	10.1016/j.scitotenv.2016.11.134
Mensah	2018	10.1007/s00442-018-4144-0
Mensah	2016	10.3390/f7040079
Mensah	2020	10.1111/1365-2435.13585
Miranda	2014	10.1007/s10531-014-0813-4
Mitchell	2007	10.2307/3071990
Mölder	2008	10.1016/j.foreco.2008.04.012
Momota	2017	10.7717/peerj.2952
Montesinos-Navarro	2014	10.1093/aob/mcu228
Montoya	2003	10.1046/j.1461-0248.2003.00469.x
Mori	2017	10.1111/oik.04345
Mouillot	2014	10.1073/pnas.1317625111
Muraina	2021	10.1111/1365-2745.13587
Na	2016	10.1071/RJ15094
Naeem	1995	10.1098/rstb.1995.0025
Naeem	2007	10.1890/0012-9658(1998)079[2603:CSRAAB]2.0.CO;2
Naeem	2006	10.2307/3546198
Nakamoto	2018	10.1016/j.ecss.2018.03.020
Nascimento	2010	10.1007/s00442-010-1865-0
Nash	2021	10.1111/1365-2656.13505
Ng	2022	10.1111/ddi.13526
Novoselov	2022	10.1134/S1067413622030080
Núñez-Olivera	2004	10.1007/BF00045504
Oehri	2017	10.1073/pnas.1703928114
Oindo	2002	10.1046/j.1365-2028.2001.00322.x
Ojha	2017	10.1371/journal.pone.0187106

Omidipour	2020	10.1016/J.ECOLIND.2020.107212
Oram	2020	10.1111/1365-2664.13667
Orford	2016	10.1111/1365-2664.12608
Otwoma	2017	10.1007/s10750-017-3363-4
Ouyang	2019	10.1111/1365-2745.13194
Ouyang	2020	10.1111/geb.13235
Paijmans	2020	10.1038/s41598-020-61560-8
Palmu	2014	10.1016/j.baae.2014.03.001
Parolo	2011	10.1111/j.1365-2494.2011.00810.x
Pasari	2013	10.1073/pnas.1220333110
Pastore	2021	10.1073/pnas.2016965118
Pawlowska	2011	10.1007/s00300-010-0940-7
Pedro	2017	10.1111/jvs.12505
Peng	2017	10.1093/jpe/rtw016
Pennekamp	2018	10.1038/s41586-018-0627-8
Pérez-Sánchez	2021	10.1111/een.13069
Perner	2005	10.1111/j.0906-7590.2005.04119.x
Perring	2015	10.1016/j.foreco.2015.02.012
Pfisterer	2014	10.1007/s00442-002-1169-0
Pfisterer	2005	10.1078/1439-1791-00215
Phillips	2008	10.1016/j.rse.2008.04.012
Phillips	2021	10.1038/s41597-021-00912-z
Pigot	2016	10.1371/journal.pbio.1002407
Pinto	2012	10.1016/j.ecolind.2012.06.022
Pinto	2018	10.1016/j.foreco.2018.03.029
Pires	1905	Plant species richness increases the spatial stability of litter mass in Brazilian Pantanal
Polley	2003	10.1046/j.1461-0248.2003.00422.x
Porazinska	2021	10.1016/j.soilbio.2021.108380
Post	2013	10.1098/rspb.2012.2722
Pozzobom	2020	10.1007/s00027-020-00730-2
Prommer	2019	10.1111/gcb.14777
Proulx	2010	10.1371/journal.pone.0013382
Qi	2022	10.3389/fpls.2021.772503
Qiao	2022	10.1111/geb.13488
Radinger	2019	10.1111/ddi.12895
Raes	2018	10.1073/pnas.1719335115
Remmer	2019	10.1080/02705060.2019.1614104
Ren	1905	The influences of small dam on macroinvertebrates diversity and temporal stability in stream ecosystem
Rey	2016	10.1111/nph.13916
Rixen	2005	https://doi.org/10.1007/s00442-005-0196-z
Roca	2019	10.1002/rse2.129

Rodil	2021	10.1007/s10531-021-02234-3
Roger	2016	10.1002/ecy.1518
Ronk	2016	10.1007/s10531-016-1278-4
Roscher	2008	10.1016/j.actao.2008.10.003
Roscher	2016	10.1016/j.ppees.2016.03.003
Rosenfield	1905	Forest biomass variation in Southernmost Brazil: the impact of Araucaria trees
Rovai	2021	10.1111/geb.13268
Ruiz-Benito	2013	10.1111/geb.12126
Rybicki	2010	10.4319/lo.2007.52.3.1195
Rychtecká	2014	10.1007/s00114-014-1198-7
Sagar	2017	10.1093/jpe/rtx052
Salgado	2021	10.1002/rra.3839
Sandin	2008	10.1111/j.1466-8238.2008.00418.x
Sang	2021	10.1186/s13717-021-00337-x
Santos	2014	10.1016/j.ecolind.2014.08.033
Sapijanskas	2014	10.1890/13-1366.1.hal-02964493
Sarneel	2014	10.1007/s10021-014-9805-1
Sasaki	2021	10.1016/j.jaridenv.2021.104690
Sasaki	2019	10.1111/1365-2745.13151
Schaefer	2012	10.1899/11-107.1
Schmera	2012	10.1007/s10750-012-1107-z
Schmidt	2021	10.1101/2021.01.04.425301
Schmidt	2021	10.1111/evo.14407
Schmidt	2022	10.1111/jbi.14480
Schnabel	2019	10.1111/gcb.14792
Schuldt	2022	10.1111/1365-2664.14300
Seabloom	2013	10.1111/gcb.12370
Segura	2021	10.3390/f12121730
Selvi	2016	10.1016/j.foreco.2016.02.013
Setiawan	2016	10.1007/s10531-015-1044-z
Setshedi	2020	10.3390/agriculture10090395
Shumi	2020	10.1007/s10980-020-01170-x
Shunatova	2018	10.1007/s00300-018-2263-z
Siddique	2021	10.1016/j.foreco.2020.118848
Slocum	2008	10.1111/j.1365-2427.2008.02002.x
Smith	2003	10.1046/j.1461-0248.2003.00454.x
Sobek	2009	10.1016/j.foreco.2009.04.026
Sobek	2009	10.1111/j.1472-4642.2009.00570.x
Song	2019	10.1016/j.foreco.2019.04.015
Song	2012	10.1111/j.1365-2486.2012.02738.x
Souza	2020	10.1590/1678-4766e2020027
Speed	2019	10.1111/ecog.04347

Spehn	2003	10.1046/j.1365-2435.2000.00437.x
Spehn	2007	10.1890/03-4101
Staab	2014	10.1111/een.12143
Stachowicz	2002	10.1126/science.286.5444.1577
Steinauer	1905	10.1890/14-0088..1
Steudel	2016	10.1111/nph.14054
Stojek	2022	10.1016/j.foreco.2022.120451
Strecker	2016	10.1111/oik.03181
Sugie	2016	10.1002/lno.10473
Sun	2018	10.1007/s11104-018-3877-1
Symonds	2008	10.1086/528960
Symstad	2006	10.1641/0006-3568(2003)053[0089:LTALSP]2.0.CO;2
Symstad	2006	10.2307/3547058
Taft	2017	10.1007/s11829-017-9539-z
Takyu	2005	10.1007/s11284-005-0044-y
Taniguchi	2022	10.1016/j.ecolind.2022.108686
Tao	2020	10.3161/15052249PJE2020.68.1.006
Tian	2017	10.3390/ijerph14010095
Tian	2017	10.1007/s11356-017-8758-2
Tilman	2003	10.1038/367363a0
Tilman	2003	10.1038/379718a0
Tilman	2006	10.1038/nature04742
Tilman	2002	10.1126/science.277.5330.1300
Toledo-Lima	2020	10.1007/s43388-020-00016-z
Travers	2011	10.3354/meps09501
Tsuyuzaki	2017	10.1007/s00300-017-2236-7
Tylianakis	2007	10.1890/0012-9658(2006)87[3047:DEFASO]2.0.CO;2
Uyttenbroeck	2017	10.1016/j.agee.2017.08.014
Vadrucci	2003	10.1002/aqc.590
Valencia	2020	10.1073/pnas.1920405117
Valliere	2020	10.1002/ecs2.3032
Van Con	2013	10.1016/j.foreco.2013.08.034
van der Heijden	2002	10.1038/23932
van der Plas	2016	10.1073/pnas.1517903113
Van der Putten	2002	10.1007/s004420050028
van Rooijen	2015	10.1007/s10021-015-9905-6
Van Voris	2006	10.2307/1939044
Vargas-Larreta	2021	10.1186/s40663-021-00282-3
Vasconcelos	2019	10.1007/s10531-019-01708-9
Velghe	2012	10.1111/fwb.12017
Verma	2020	10.1016/j.ecolind.2020.106987

Vetaas	2019	10.1111/jbi.13564
Vetter	2020	10.1111/gcb.15025
Vibrans	2022	10.1016/jforeco.2021.119902
Vilmi	2017	10.1111/ddi.12594
Virtanen	2012	10.1111/j.1600-0587.2012.07903.x
Vogy	2010	10.1111/j.1365-2427.2010.02397.x
von Felten	2012	10.1890/11-1439.1
Wacker	2007	10.1016/j.baae.2007.08.003
Wagg	2021	10.7554/eLife.62813
Wang	2013	10.1007/s00267-013-0186-6
Wang	2016	10.3390/rs8030214
Wang	2016	10.1007/s11104-016-2897-y
Wang	2022	10.1016/j.ecolind.2022.108858
Wang	2021	10.1016/j.envint.2021.106494
Wang	2018	10.1016/j.rse.2018.04.010
Wang	2022	10.1016/j.rse.2022.113169
Wang	2019	10.1038/s41467-019-11191-z
Wang	2007	10.1111/j.1461-0248.2007.01031.x
Wardle	2004	10.1034/j.1600-0706.2000.890102.x
Wehn	2018	10.5194/we-18-55-2018
Wein	2016	10.1371/journal.pone.0168751
Weisser	2017	10.1016/j.baae.2017.06.002
Wesner	2011	10.1111/j.1600-0706.2011.19413.x
Weyhenmeyer	2012	10.1111/j.1365-2427.2012.02779.x
Whitford	2002	10.1023/A:1005987219356
Wilsey	2007	10.1890/04-0245
Wilson	2009	10.1111/j.1365-2486.2009.01896.x
Witman	2008	10.1890/07-1201.1
Wittebolle	2009	10.1038/nature07840
Wondimu	2021	https://doi.org/10.1186/s13717-021-00322-4
Wu	2018	10.1016/j.jenvman.2018.10.097
Wu	2014	10.1111/ecog.00940
Xu	2019	10.1016/j.gecco.2019.e00832
Yang	2019	10.1007/s40333-019-0068-8
Yang	2012	10.1111/j.1461-0248.2012.01778.x
Ye	2019	10.1111/1365-2745.13192
Yuan	2018	10.1016/j.scitotenv.2018.02.125
Zakkak	2015	10.1016/j.jenvman.2015.09.005
Ze Zhang	2022	10.1002/ece3.9385
Zhang	2012	10.1016/j.atmosenv.2012.08.034
Zhang	2022	10.1016/j.scitotenv.2022.156947
Zhang	2017	10.1111/1365-2745.12903
Zhang	2015	10.1111/gcb.13140

Zhang	2021	10.1111/geb.13408
Zhao	2018	10.1016/j.aspen.2018.01.017
Zhao	2018	10.1093/aesa/say038
Zheng	2019	10.1111/1365-2745.13142
Zhou	2022	10.1007/s10021-022-00790-5
Zhou	2022	10.3390/su141912152
Zhou	2022	10.1016/j.soilbio.2022.108774
Zhou	2006	10.1111/j.1365-2435.2006.01175.x
Zirbel	2019	10.1002/ecy.2634
Ziter	2013	10.1890/ES13-00135.1
Zuo	2019	10.1016/j.gecco.2019.e00864
