# Supplementary material for: Biodiversity safeguards ecosystem services and functions worldwide
Source: Nat Ecol Evol. 2026 Oct 6;10(11):2037–48. doi: 10.1038/s41559-026-03200-4 (PMC13736519; doi:10.1038/s41559-026-03200-4)
Supplement: Supplementary file 3 [file 41559_2026_3200_MOESM3_ESM.pdf]

nature portfolio

Peer Review File

Biodiversity safeguards ecosystem services and functions worldwide

Corresponding Author: Dr Emma Moffett

Version 0:

Decision Letter:

27th February 2026

*Please ensure you delete the link to your author homepage in this e-mail if you wish to forward it to your co-authors.

Dear Dr Moffett,

Your manuscript entitled "From oceans to forests, biodiversity powers climate-regulating services" has now been seen by 3 reviewers, whose comments are attached. The reviewers have raised a number of concerns which will need to be addressed before we can offer publication in Nature Ecology & Evolution. We will therefore need to see your responses to the criticisms raised and to some editorial concerns, along with a revised manuscript, before we can reach a final decision regarding publication.

In particular, while all three reviewers were largely positive about the synthesis, they also feel that more clarity is needed on the data presentation, and statistical analysis. Reviewer #1 feels that a clearer distinction needs to be made between ecosystem services and functions, and both Reviewers #1 and #2 feel that the forecasting in relation to food security is oversimplified; Reviewer #2 suggests reframing this in terms of pest control services instead.

At this stage we also suggest revising your title to be more specific and declarative; this point is also raised by Reviewer #2.

We therefore invite you to revise your manuscript taking into account all reviewer and editor comments. Please highlight all changes in the manuscript text file [OPTIONAL: in Microsoft Word format].

We are committed to providing a fair and constructive peer-review process. Do not hesitate to contact us if there are specific requests from the reviewers that you believe are technically impossible or unlikely to yield a meaningful outcome.

When revising your manuscript:

* Include a “Response to reviewers” document detailing, point-by-point, how you addressed each reviewer comment. If no action was taken to address a point, you must provide a compelling argument. This response will be sent back to the reviewers along with the revised manuscript.

* If you have not done so already please begin to revise your manuscript so that it conforms to our Article format instructions at http://www.nature.com/natecolevol/info/final-submission. Refer also to any guidelines provided in this letter.

* Include a revised version of any required reporting checklist. It will be available to referees (and, potentially, statisticians) to aid in their evaluation if the manuscript goes back for peer review. A revised checklist is essential for re-review of the paper.

* Extended Data Figures - please ensure that any supplementary figures and tables that are crucial to the manuscript’s conclusions are converted into Extended Data figures and tables to increase visibility of these data. Extended Data figures and tables are online-only (present in the online PDF and full-text HTML versions of the paper), peer-reviewed display items that provide essential background to the article but are not included in the main article due to space constraints. A maximum of ten Extended Data display items (figures and tables) is permitted.

Please use the link below to submit your revised manuscript and related files:

Link Redacted

Note: This URL links to your confidential home page and associated information about manuscripts you may have submitted, or that you are reviewing for us. If you wish to forward this email to co-authors, please delete the link to your homepage.

We hope to receive your revised manuscript within four to eight weeks. If you cannot send it within this time, please let us know. We will be happy to consider your revision so long as nothing similar has been accepted for publication at Nature Ecology & Evolution or published elsewhere.

Nature Ecology & Evolution is committed to improving transparency in authorship. As part of our efforts in this direction, we are now requesting that all authors identified as 'corresponding author' on published papers create and link their Open Researcher and Contributor Identifier (ORCID) with their account on the Manuscript Tracking System (MTS), prior to acceptance. ORCID helps the scientific community achieve unambiguous attribution of all scholarly contributions. You can create and link your ORCID from the home page of the MTS by clicking on 'Modify my Springer Nature account'. For more information please visit please visit www.springernature.com/orcid.

Please do not hesitate to contact me if you have any questions or would like to discuss these revisions further.

We look forward to seeing the revised manuscript and thank you for the opportunity to review your work.

[redacted]

Reviewer expertise:

Reviewer #1: Ecosystem services, synthesis

Reviewer #2: Ecosystem services

Reviewer #3: Ecosystem services

Reviewers' comments:

Reviewer #1 (Remarks to the Author):

Thank you for the opportunity to review the manuscript "From oceans to forests, biodiversity powers climate-regulating services" for Nature Ecology & Evolution. I enjoyed reading this paper, which describes a large global meta-analysis of the relationships between biodiversity and ecosystem services, which is a critical and long-standing scientific knowledge gap. The authors find different types of relationships between BD and ES (e.g., linear, saturating) and commonly positive BES relationships, although the strength of these relationships vary substantially but are strongest for marine climate regulation. The authors then attempt to demonstrate the usefulness of their approach by using their understanding of one BES relationship to forecast how potential future changes in BD might impact future food and feed provision.

I appreciate the significant time and effort that the authors have put into this analysis and I do think that there are some important findings that come out of it that should be published. However, at present I also have concerns about how the authors define ES, how they present their results and the strength of those results, and in particular how they have used this information to forecast future levels of food and feed. I detail my major comments below.

Major Issues

(1) The authors make the point early on in the manuscript of the distinction between biodiversity-ecosystem functon(BEF) relationships versus biodiversity-ecosystem service (BES) relationships, and that while the former type are well-established, BES relationships are less understood. I agree with this and that the distinction between ecosystem functions (the flow of energy and materials through the arrangement of biotic and abiotic components of an ecosystem) and ecosystem services (which specifically include consideration of benefits to people or a contribution human livelihood). However, after this point in the text, I feel that these two terms become confused, especially with respect to the response variables in the meta-analysis, which makes it difficult to evaluate and interpret the results. For example, the ES included in the results (e.g., Figure 2) include ES as defined by IPBES (e.g., regulation of air quality, regulation of climate, maintenance of options) but also variables that I would classify as EF and that, to the best of my knowledge, are not defined as ES by IPBES (e.g., evapotranspiration, biomass turnover rate, multifunctionality, biological pump efficiencies). I think this distinction between EF and ES is important, as currently the lack of distinction makes it much more difficult to interpret the results and evaluate how this analysis and paper advances our knowledge specifically of BES relationships. In addition, the expectation of how an EF might respond to a change in BD, versus how an ES that often incorporates human benefits, values, and demand could differ substantially.

(2) The authors provide a large number of results for different ES and EF with respect to the most supported form of the relationship (linear, quadratic, logarithmic) and the effect size of biodiversity responses. These are all useful to understand the form and strength of these relationships across a large number of ES/EF. However, one key result that I found missing in the main text or appendices was some information on the fit of the various models (e.g. R2 value or % deviance explained)

to better understand how well these models explain the overall variation in the ES/EF. While understanding the BES relationship is important, understanding what relative effect this has on ES provision is also important for identifying key actions or strategies for increasing ES provision. In other words, I believe this would help understand the statistical significance of the results versus the real-world significance.

(3)I have some significant questions about the methods and approach to forecast future changes to food security and abundance. First, this approach models the ratio of natural enemies to pests based on future predictions of the Biodiversity Intactness Index. My questions about this approach include:

The relationship between pest regulation and food/feed production is complex and context-dependent in ways that I don't feel the current approach as performed or described captures. For example, there are complicated relationships between landscape configuration and pest regulation (Karp et al. 2018. PNAS) and the spatial patterns of pest regulation and presence of pests at landscape scales, as well as complex relationships between pests and damage to crops. Just because the change in the ratio of natural enemies to pests is similar in two different locations, does not necessarily mean that the impact on crop production will be the same, as it will depend on the crops grown, the spatial patterns of crop and natural features, and the ability of farmers to address and mitigate these changes. Whether these types of dynamics have been included in the current modelling approach is not clear.

In addition to the complexity of relationships between natural enemies, pests, and crop production, future changes in climate will also have important effects on rainfall, temperatures, and humidity, which themselves will also impact crop health and production. The current lack of incorporation of these types of changes in the modelling also makes it difficult to interpret what impacts to food/feed future BD changes could have.

The ratio of natural enemies to pests is an interesting and potentially useful variable to model, but it's appropriateness also depends on whether this is a common variable that was measured for pest regulation in the papers that contributed to the meta-analysis. If different or varied measure of pest regulation were present in those papers, then the appropriateness of using the resulting pest regulation BES relationship to model future changes in pest regulation would be in question.

Equating changes in pests and crop production to food security is somewhat tenuous at best and I think more exact wording here is necessary. Food security is a complex term with six dimensions (availability, accessibility, stability, sustainability, utilization, and agency) and in most cases, decreased food security is not a function of a lack of food abundance. Instead, it is often caused by income inequality or political instability. More careful wording here is important.

(4) There is understandably a very large number of BES papers present in the current literature and it would not be logistically possible to include all of them in a meta-analysis. However, the current methods describe choosing "the most relevant results" within ecosystem type and ecoregion, and within ES categories, requires some additional description to ensure that a representative and unbiased sample of papers was used in the analysis. In particular, it would be useful to know what specific criteria were used to determine the relevance of a paper, and whether that potentially introduced any bias with respect to the geographic representativeness or statistical significance of the papers that contributed to the meta-analysis.

Reviewer #2 (Remarks to the Author):

The paper "From oceans to forests, biodiversity powers climate-regulating services" is a valuable contribution to global ecology through the largest and most comprehensive synthesis of biodiversity-ecosystem service relationships to date. The authors aim to resolve longstanding debates about whether biodiversity contributions to ES are typically linear or saturating, demonstrating that both are common and context-dependent. The paper also has substantial policy relevance and represents a significant advance in quantifying and predicting biodiversity-ecosystem service relationships at a global scale with direct implications for conservation policy and climate mitigation strategies. I believe this thorough meta-analysis is likely to be well-cited. I have the following critiques and queries:

Introduction/Framing

The title ("From oceans to forests, biodiversity powers climate-regulating services") implies the focus of the work was on climate-regulating services and did not consider others, but the meta-analysis covers all services. Perhaps the title could be more general (something like "From oceans to forests, biodiversity underpins ecosystem services"), with the abstract used to emphasise the important findings surrounding climate-regulating services?

I also wonder if ocean C should be the focus when "Oceanic carbon sequestration was especially underrepresented (n = 7), with far fewer datasets in our database than for terrestrial carbon sequestration (n = 154)" Perhaps focus on carbon in general (as terrestrial carbon also had a high effect size). This could then speak to all carbon sequestration initiatives and help challenge the paradigm of fast-growing monocultures for C credits.

Literature sampling

The methods state: "To reduce the number of papers while ensuring a representative spread of data, we used a stratified survey method. After first filtering the literature search results by ecosystem type and ecoregion, we took the 100 most relevant results from each ecosystem type and ecoregion, resulting in 3875 papers." How were the "most relevant" papers

selected? What was the criteria?

In relation to this the authors state (Line 227) “Existing literature is geographically biased toward developed countries (Fig. 1A), underrepresenting regions critical for global food production, like South America and Africa44. To address this gap, we developed a novel heuristic modelling framework that, for the first time, links global BES relationships with projected climate and land-use change”. This had me wonder why this bias was not reduced by specifically sampling from different regions (as the authors did for ecosystem type)?

The role of biodiversity measures

I understand that you compiled studies that used different measures of biodiversity. As the studies use diverse metrics (e.g., species richness, evenness, functional and phylogenetic diversity), among other differences, so these data were rescaled. This is definitely a sensible approach, for the main analysis. However, I wonder if these different measures of biodiversity affected the findings, particularly the nature of the relationship (linear etc) between biodiversity and services – phylogenetic diversity is very different to species richness. Were these different measures relatively evenly represented across each service type? Were the different relationships (linear, saturating) over-represented by a particular biodiversity measure?

Ecosystem services conflated with food production

A significant concern is that the food and feed provisioning forecast conflates ecosystem service provision with actual agricultural output. The paper models the decline in natural pest control services under biodiversity loss but presents this as predicting reduced food production, which is problematic because modern industrial agriculture has substantially decoupled food output from natural ES through chemical pesticides, crop intensification, and technological innovation. Whilst the paper correctly identifies that natural enemies provide biological pest control, this figure likely applies primarily to low-input or organic systems rather than global agriculture as a whole. Critically, countries with high human development index values and intensive agricultural development often produce more food despite lower biodiversity, because they can afford pesticide and fertiliser inputs. The forecast therefore predicts ES loss rather than food production loss, and the link to food insecurity depends on socio-economic factors (poverty, market access, farmer access to inputs) that the model does not capture. A more defensible interpretation would frame the finding as a loss of natural pest control services that creates dependency on chemical inputs, with implications for environmental sustainability and long-term agricultural resilience, rather than a direct threat to food production. The paper should clarify this distinction, acknowledge how agricultural intensification can compensate for ES loss, and contextualise the forecast to specific agricultural systems where natural pest control remains critical.

Climate scenarios

The methods describe 5 IPCC climate change scenarios and a monte carlo sampling method to propagate uncertainty but only 2 scenarios are shown in the results (main text or supplementary) and I cannot find any spatial depiction/description of this uncertainty. Additionally, what GCMs are used in the climate scenarios selected? Are they ensemble averages?

Minor comments
Line 48 – BES acronym not previously defined
Figures seem to have spelling error “compansionship”
Lines 108 -109 “No ES can rise indefinitely with biodiversity, but our data suggest saturation limits have rarely been reached, implying functional redundancy may be overestimated”. This has rarely been reached in the experiments? Surely it has been reached in reality? This should be clarified.
234-246 a whole paragraph focused on methods within the results/discussion
Extended data figure 3. Terrestrial, marine, freshwater and estuarine should be aligned across graphs (even if there’s a gap in the case where a category is missing) for easier interpretation

Reviewer #3 (Remarks to the Author):

This manuscript is a substantial synthesis of the biodiversity literature in terrestrial, aquatic and marine ecosystems and draws conclusions about how much biodiversity is necessary to maintain certain types of ecosystem services. And the authors forecast what biodiversity loss is expected with 2 different climate change scenarios and what that will mean for food security in the future.
This manuscript is fairly well-written and will make a good contribution to the biodiversity literature with some revisions for clarity. I have the following suggestions to improve the manuscript:
1) The discussion of the different analyses of effect sizes vs which services were more or less sensitive to biodiversity were slightly confusing (Results in Figure 2 vs Figure 3). The authors point out that regulating services including hazards were typically a linear relationship, (lines 95-96), but then the authors say that hazard regulation was insensitive to biodiversity (lines 145-146). It would be helpful to discuss the nuances of these findings and how different analyses led to somewhat different conclusions about hazards and biodiversity. What do these two different findings mean and how are they distinct and useful in and of themselves but also when put together?
2) There are some terms that need to be explained briefly/defined in the text because they need to be understood by the reader if the reader does not go read the full methods: spatial grain and Human Development Index. Even after reading the

methods section, these terms were not immediately obvious and clear. Also, how/why they relate to biodiversity is not necessarily clear. For the HDI, what does a high vs a low HDI mean for people in that country? And how might this relate to biodiversity? Why did the authors select HDI to use in this analysis?
3) If the point of Extended Data Figure 2 is the better understand if experimental vs observational studies show the same relationship with biodiversity increases, it would be nice to color code the ones where the relationship is different depending on the study type, so it is more obvious for the reader which ones are different vs which ones show the same type of relationship no matter which kind of study was used.
4) The authors point out that sampling and design biases could be contributing to their findings (for example lines 199-200). It might be helpful to include a bit more discussion of what the authors think they can state unequivocally and also what might be due to a lack of studies in particular ecosystems or at particular spatial scales, etc. A bit more discussion of how to interpret these findings would be helpful. I think these findings are very interesting and useful, and also would like to ensure that the results are taken within the context of what is still less studied and may change as we have more study results in the future.
5) The case study looking at future climate change and ecosystem services is an interesting idea. However, it would be useful to have a little more discussion of how the modeling effort works and also the results. Why are there the predicted differences in food security between different regions? What is driving those differences? Is it primarily climate change impact differences or human demographic differences or both? How do the human demographics interact with the climate change predictions? This is where having a better explanation of HDI above will hopefully also help with clarifying the results of the case study more as well. The case study right now feels as if it is not well enough explained to add as much value to the paper as I had hoped.

Additional comments:
1) Do we have a sense for why the ocean carbon sequestration is so sensitive to biodiversity? (lines 138-145) And could this sensitivity be driven at all by the fact that there were only a few studies the authors found that dealt with ocean carbon sequestration?
2) Lines 602: I would add in what a high HDI means… vs low HDI.

********************END********************

Version 1:

Decision Letter:

14th June 2026

Dear Dr. Moffett,

Thank you for submitting your revised manuscript "Biodiversity safeguards ecosystem services and functions worldwide" (NATECOLEVOL-26010231A). It has now been seen again by the original reviewers and their comments are below. The reviewers find that the paper has improved in revision, and therefore we'll be happy in principle to publish it in Nature Ecology & Evolution, pending minor revisions to satisfy the reviewers' final requests and to comply with our editorial and formatting guidelines.

If the current version of your manuscript is in a PDF format, please email us a copy of the file in an editable format (Microsoft Word or LaTex)-- we can not proceed with PDFs at this stage.

If you have not done so already, please ensure that you also email us a completed copy of the Reporting summary :

Reporting summary: https://www.nature.com/documents/nr-reporting-summary.pdf

We are now performing detailed checks on your paper and will send you a checklist detailing our editorial and formatting requirements in a couple of weeks. Please do not upload the final materials and make any revisions until you receive this additional information from us.

Thank you again for your interest in Nature Ecology & Evolution. Please do not hesitate to contact me if you have any questions.

[redacted]

Reviewer #1 (Remarks to the Author):

Thank you for the opportunity to review the revised manuscript "Biodiversity safeguards ecosystem services and functions

worldwide." Overall, I think that the authors have done a good job addressing the reviewer comments, and in particular my comments, on the initial submission. While I do have some minor comments that I detail below, I congratulate the authors on their substantial and important work and look forward to seeing this paper in print.

1. L48-49: I'm not sure how forecasting future BD change impacts on ES highlights how ecosystem services might be used to address future problems ahead of time. However, I can see how this highlights the importance of conserving biodiversity for ecosystem services and the benefits they provide.

2. L123: I would probably not use "underpins" here. Pest regulation can be an important contribution to food and feed provision, but depending on the circumstances might be relatively unimportant to that ES.

3. L143: I would include a definition of what you consider "low-to-moderate r2 values" as this interpretation will likely differ across readers. You could include a range of values here.

4. L155: Awkward wording with "...relationships and within relationships in well-studied categories..."

5. L172: I'm a little unsure what this sentence is trying to say as a main point. Perhaps an example here would help clarify?

6. L266: There seems to be a bit of contradiction between this sentence that states "biodiversity effects strengthened with increasing grain" and later on in the paragraph (L273-274) state that biodiversity contributions to a number of ES decline with increasing spatial grain.

7. L301-302: I would probably define here what you mean by fine spatial grain versus broader-scale studies. Readers might interpret these categories differently based on the type of research they are familiar with.

8. L311: Weaker and negative?

9. L332: Awkward wording here with "to get closer to elucidate"

10. L395: This sentence makes me wonder if your model is predicting just natural enemy numbers or the ratio between predators and pests? At L351 you state that you focus on the ratio of natural enemies to pests, but L395 specifically mentions natural enemy declines. I'm not sure these details are described in the methods section either. It would be beneficial to make clearer how changes in Biodiversity Intactness were translated into the predator:pest ratio, especially since a change in this ratio could happen due to changes in either predators or pests (or both).

11. L401: Does greater levels of pest regulation in countries with higher HDI values represent just a maintenance of current levels, or actual increases in this ES and "potential gains for agricultural systems"?

12. L522: Should this say "protection provided from pests by natural enemies"? I don't recall pathogens being mentioned in the main text of the manuscript.

13. L672-673: In the main text I think spatial grain is described as the spatial grain, but here "spatial extent" is also mentioned. Usually a study will have both a spatial extent (to complete spatial extent of the measurements/plots across the whole study) and spatial grain (at what scale individual measurements are taken). It should be very clear what was actually measured, and how this was done (i.e., how was spatial grain determined for each paper).

14. One last thought that I will leave to the authors to think about is around the robustness of your forecasted future changes to ecosystem services and the use of Biodiversity Intactness Index. A number of the BES and BEF relationships that were found are not linear, but are saturating or quadratic, which might reflect differential contributions of specific species to EF/ES based on functional traits. I am guessing (but might be wrong) that the predicted BII values don't include prediction about what specific species will be lost for each SSP. Thus, is it possible that the current modelling approach might under- or overestimate changes in EF/ES if species loss is not a random process across species in each scenario? If yes, then I think this could be something important to mention briefly in the paper as a limitation of the current modelling process. However, I might be misunderstanding the model construction and details, and in that case this comment can be ignored.

Reviewer #2 (Remarks to the Author):

I would like to commend the authors on their thorough revisions to this paper. I would also like to reiterate the importance of this paper in providing a comprehensive synthesis of biodiversity-ecosystem service relationships, with direct implications for conservation policy. I believe the authors have now addressed all of my main concerns, as detailed below:

Introduction/framing: The new title is much improved and more representative of the broad scope of the paper.

Literature sampling: Additional clarity on the literature review search methods which has helped clarify how relevant papers were selected.

The role of biodiversity measures:
In response to my point about if the different measures of biodiversity were influencing the results, the authors conducted a

new (thorough) analysis to answer this question. While they found that functional and phylogenetic diversity were disproportionately associated with saturating relationships (relative to taxonomic diversity), their dataset was dominated by taxonomic diversity measures, and as such they believe (and I agree) that strong claims cannot be made about metric differences. As such, they have included this analysis in the supplementary materials (with a mention in the main text) which is appropriate. This new analysis and findings do not change their main conclusions but add depth to the paper.

Ecosystem services are conflated with food production:
I was also concerned that their predictions of future change in pest control services were conflating the change in the ES with a change in agricultural output (another reviewer made a similar comment). They have now changed the how this analysis is described (including in the abstract), which I believe now makes it clear they are predicting a change in the ecosystem service. Predicting a change in actual agricultural output is well beyond the scope of this manuscript, so I believe their clarification of the future predictions is sufficient, and the change in the (biodiversity mediated) pest control ecosystem service is important and novel.

Climate scenarios:
The authors have now also now included the results for the 5 SSP scenarios and propagation of uncertainty which were described in the methods (along with further clarification of the methods). Including all 5 scenarios and the uncertainty surrounding them is an important addition to this modelling effort.

Reviewer #3 (Remarks to the Author):

I am satisfied with the reviewers' responses to my comments.

INTERNAL USE ONLY AIP modules required:["Life Science"]

Open Access This Peer Review File is licensed under a Creative Commons Attribution 4.0 International License, which permits use, sharing, adaptation, distribution and reproduction in any medium or format, as long as you give appropriate credit to the original author(s) and the source, provide a link to the Creative Commons license, and indicate if changes were made.
In cases where reviewers are anonymous, credit should be given to 'Anonymous Referee' and the source.
The images or other third party material in this Peer Review File are included in the article’s Creative Commons license, unless indicated otherwise in a credit line to the material. If material is not included in the article’s Creative Commons license and your intended use is not permitted by statutory regulation or exceeds the permitted use, you will need to obtain permission directly from the copyright holder.
To view a copy of this license, visit https://creativecommons.org/licenses/by/4.0/

Response to reviewers

We thank the editorial team and the three reviewers for their careful evaluation of our manuscript. We have integrated all the suggestions, which have improved the manuscript throughout. We provide a point-by-point response to each of the reviewers' comments below, *in blue italics*.

Reviewer #1 (Remarks to the Author):

Thank you for the opportunity to review the manuscript "From oceans to forests, biodiversity powers climate-regulating services" for Nature Ecology & Evolution. I enjoyed reading this paper, which describes a large global meta-analysis of the relationships between biodiversity and ecosystem services, which is a critical and long-standing scientific knowledge gap. The authors find different types of relationships between BD and ES (e.g., linear, saturating) and commonly positive BES relationships, although the strength of these relationships vary substantially but are strongest for marine climate regulation. The authors then attempt to demonstrate the usefulness of their approach by using their understanding of one BES relationship to forecast how potential future changes in BD might impact future food and feed provision.

I appreciate the significant time and effort that the authors have put into this analysis and I do think that there are some important findings that come out of it that should be published. However, at present, I also have concerns about how the authors define ES, how they present their results and the strength of those results, and in particular, how they have used this information to forecast future levels of food and feed. I detail my major comments below.

Major Issues

(1) The authors make the point early on in the manuscript of the distinction between biodiversity-ecosystem function (BEF) relationships versus biodiversity-ecosystem service (BES) relationships, and that while the former type are well-established, BES relationships are less understood. I agree with this and that the distinction between ecosystem functions (the flow of energy and materials through the arrangement of biotic and abiotic components of an ecosystem) and ecosystem services (which specifically include consideration of benefits to people or a contribution human livelihood). However, after this point in the text, I feel that these two terms become confused, especially with respect to the response variables in the meta-analysis, which makes it difficult to evaluate and interpret the results. For example, the ES included in the results (e.g., Figure 2) include ES as defined by IPBES (e.g., regulation of air quality, regulation of climate, maintenance of options) but also variables that I would classify as EF and that, to the best of my knowledge, are not defined as ES by IPBES (e.g., evapotranspiration, biomass turnover rate, multifunctionality, biological pump efficiencies). I think this distinction between EF and ES is important, as currently the lack of distinction makes it much more difficult to interpret the results and evaluate how this analysis and paper advances our knowledge, specifically of BES relationships. In addition, the expectation of how an EF

might respond to a change in BD, versus how an ES that often incorporates human benefits, values, and demand could differ substantially.

We thank the reviewer for this thoughtful and important comment. We should have more clearly marked out how our approach maps onto the IPBES framework, and also the distinction between functions and services, and we hope the reviewer will agree that we have now done that. We have clarified the conceptual framework, explicitly distinguishing ecosystem functions from ecosystem services, and ensuring that terminology is used consistently throughout. We have changed the title of the MS to reflect this broader coverage and have also given more exposition in the introduction to make clear that we include aspects of the IPBES ecosystem service framework and the differences (and occasional overlap) between services and functions.

See the new text in line 63-75 in the revised MS, below:

> *"Here we follow the ecosystem service classification framework of the IPBES[11](Intergovernmental Science-Policy Platform on Biodiversity and Ecosystem Services), which recognises that ecosystem functions (processes that occur within an ecosystem) often underpin or constitute ecosystem services when they contribute to human well-being. For example, primary production can be an ecosystem service when it supports food provision or climate regulation. Accordingly, we classify response variables as ecosystem services where they align with IPBES nature's contributions to people categories or ecosystem functions, and as ecosystem functions when they do not always provide direct or indirect benefits to people, to ensure comprehensive coverage of key ecosystem processes. Many functions included here may also be described as ecosystem services in other contexts[12–14], reflecting overlapping use of the terms in the literature. We also chose to explicitly separate carbon sequestration from other services, and to assess its across terrestrial and marine realms, given the interest in biodiversity as a climate-regulator[15]."*

We have now revised all the Figures in the main text and supplement to explicitly label when something is a service or a function. We do not copy out all the Figures here in the response to reviews, but Figure 2 serves as a good example:

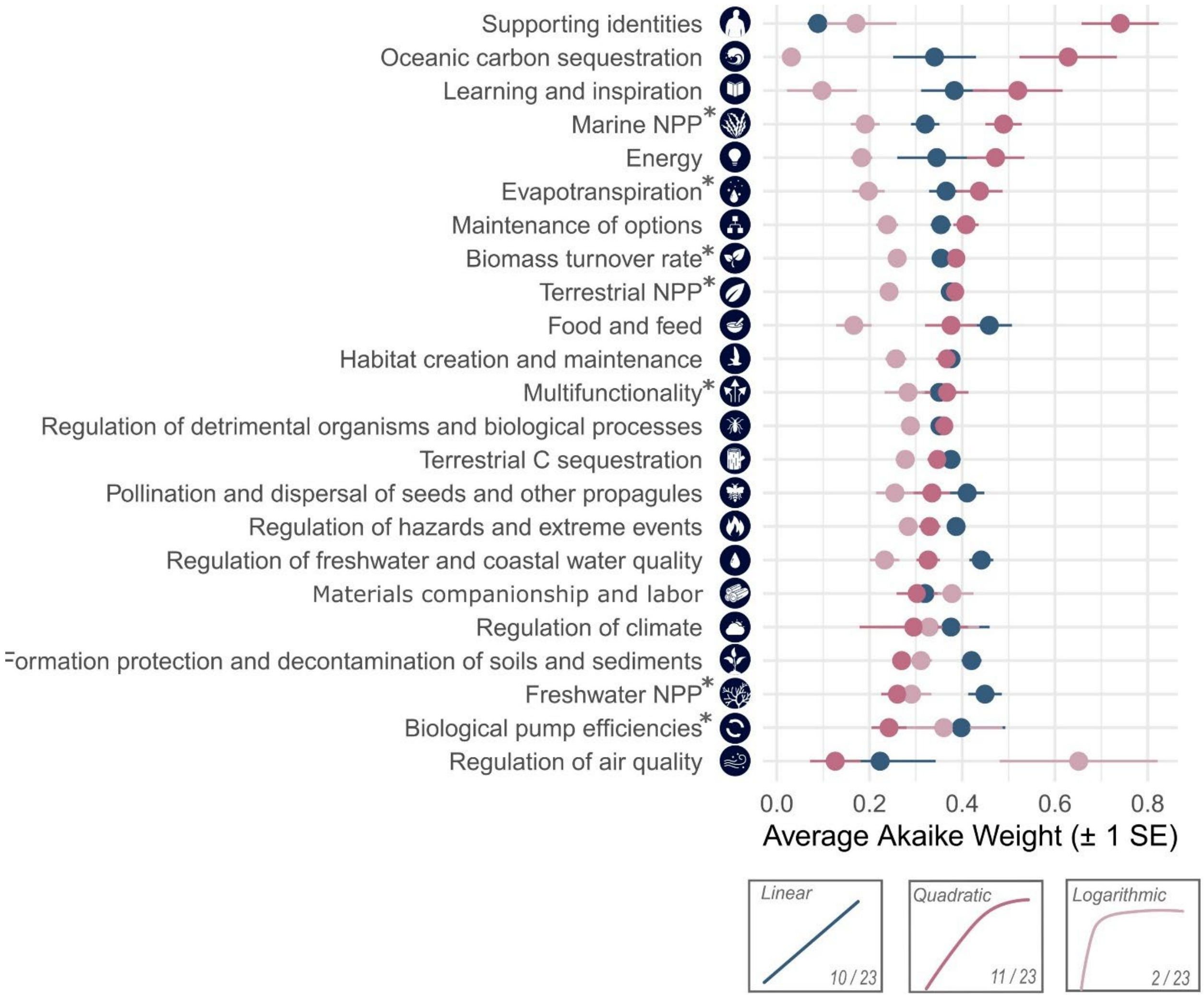

Figure 2: Linear biodiversity relationships with ecosystem services and functions are almost as common as saturating (quadratic or logarithmic) ones. *The relative likelihood that an ecosystem service category (rows; measured by Akaike Weights with error bars representing ± 1 SE) is fit by linear and saturating (quadratic or logarithmic) models. A high average Akaike weight value indicates a greater likelihood that the model type best fits the data than other model types. Most biodiversity-ecosystem relationships show quadratic (11/23) or linear (10/23) models, whereas only two are best fit by a logarithmic model (2/23). We note that these results are not induced by a splitting of variance among two saturating models; we find qualitatively identical results when comparing the best-fitting saturating model with linear (46% supporting linear, 54% supporting saturating). Data are sorted by the highest to the lowest mean value of the quadratic model. Response variables marked with an asterisk (*) are classified as ecosystem functions (EF), all other response variables are ecosystem services (ES).*

We have also revised the terminology throughout the manuscript, for example, referring to "ecosystem services and functions" rather than "BES relationships" when appropriate, using the definitions in the figures. For example:

> *"While the consequence of biodiversity declines on ecosystem functioning is well-established, the functional form and scalability of biodiversity-ecosystem service (BES) and biodiversity-ecosystem function (BEF) relationships remain a fundamental challenge" Lines 76-79.*
>
> *"Any changes in biodiversity are expected to affect ecosystem services and function when biodiversity levels fall below the saturation point." Lines 131-132.*
>
> *"In systems governed by functionally unique species, maintaining ecosystem services and functions may rely more on the persistence of these taxa than on biodiversity per se." Lines 197-199.*

We do not quote them all here for brevity, as we have made 23 such changes throughout the manuscript and supplementary materials.

(2) The authors provide a large number of results for different ES and EF with respect to the most supported form of the relationship (linear, quadratic, logarithmic) and the effect size of biodiversity responses. These are all useful to understand the form and strength of these relationships across a large number of ES/EF. However, one key result that I found missing in the main text or appendices was some information on the fit of the various models (e.g. R2 value or % deviance explained) to better understand how well these models explain the overall variation in the ES/EF. While understanding the BES relationship is important, understanding what relative effect this has on ES provision is also important for identifying key actions or strategies for increasing ES provision. In other words, I believe this would help understand the statistical significance of the results versus the real-world significance.

Thank you for this; we have followed your suggestion and now added R^2 plots to the supplementary as requested in Supplementary Figure 2. We reproduce this below, and have added explanatory text on lines 122-130 within the main text for the differences among the services' relationships below:

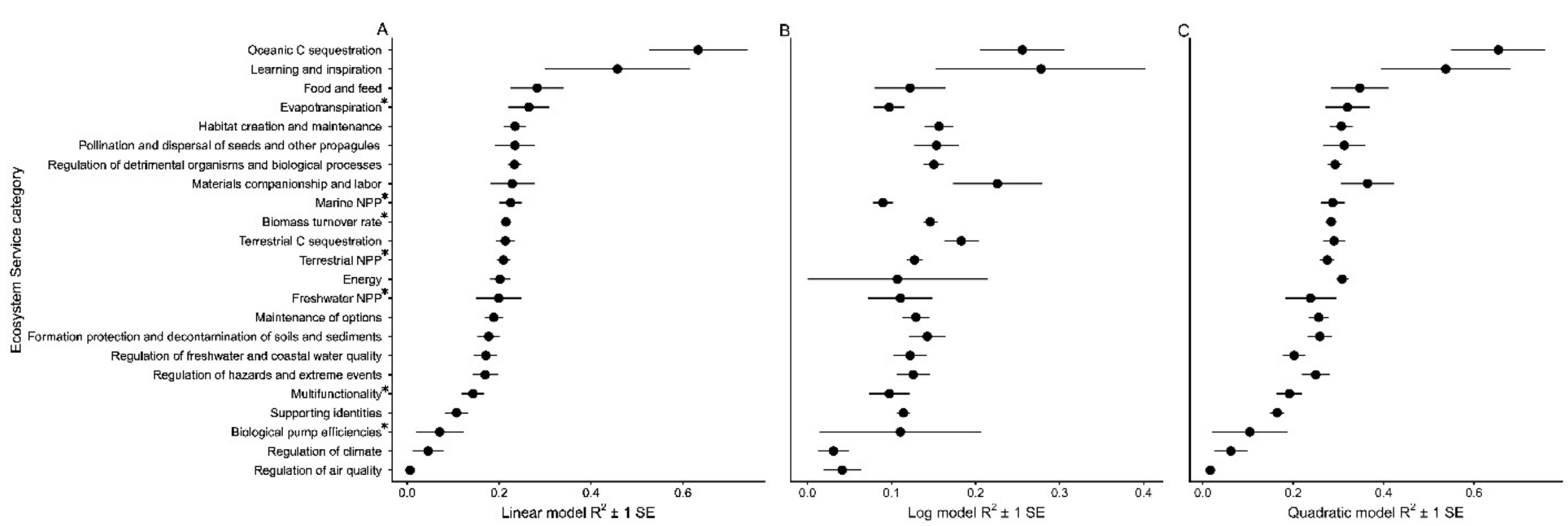

Figure S1. Comparison of linear, log, and quadratic model fits describing biodiversity-ecosystem service relationships across ecosystem function categories. Points show mean R^2 values, and error bars indicate ±1 SE across studies. Response variables marked with an asterisk (*) are classified as ecosystem functions (EF), all other response variables are ecosystem services (ES).

> *"Importantly, explanatory power varied substantially among ecosystem service and function categories and among model forms (Fig. S1). Most biodiversity-ecosystem relationships showed low-to-moderate r^2 values, indicating that biodiversity is an important but context-dependent driver of ecosystem functioning and service delivery. However, explanatory power remained weak for some services, such as hazard regulation, implying stronger influence of environmental context or functionally unique species". Lines 122-130.*

We also add r^2 values in our example of hazard regulation.

> *"Oceanic carbon sequestration, which is a significant part of the global carbon cycle, exhibits by far the strongest positive response to of any service or function category[34]. This strong sensitivity may reflect the strong biological control of marine carbon cycling, including phytoplankton productivity, microbial loop dynamics, and the biological carbon pump, all of which are closely linked to community composition and trophic structure. However, this result should be interpreted cautiously, as oceanic carbon sequestration is relatively under-represented (n = 7), with far fewer datasets in our database than for terrestrial carbon sequestration (n = 154), highlighting a critical gap in current knowledge (**Fig. 3**). This exceptional sensitivity implies that blue carbon sinks are both vulnerable to biodiversity loss and dependent on sustaining diverse communities[35,36]. Maximising the efficacy and long-term viability of blue carbon strategies will require moving beyond habitat preservation toward explicitly incorporating biodiversity as a key component[37,38]. Conversely, some services, like hazard regulation, exhibited weak overall sensitivity to biodiversity in the effect-size analysis, despite often showing linear relationships in the functional form analysis (Zr = 0, r^2 = 0.17; **Fig. 2, 3, Fig. S1**)." Lines 182-196.*

(3)I have some significant questions about the methods and approach to forecast future changes to food security and abundance. First, this approach models the ratio of natural enemies to pests based on future predictions of the Biodiversity Intactness Index. My questions about this approach include:

The relationship between pest regulation and food/feed production is complex and context-dependent in ways that I don't feel the current approach as performed or described captures. For example, there are complicated relationships between landscape configuration and pest regulation (Karp et al. 2018. PNAS) and the spatial patterns of pest regulation and presence of pests at landscape scales, as well as complex relationships between pests and damage to crops. Just because the change in the ratio of natural enemies to pests is similar in two different

locations, does not necessarily mean that the impact on crop production will be the same, as it will depend on the crops grown, the spatial patterns of crop and natural features, and the ability of farmers to address and mitigate these changes. Whether these types of dynamics have been included in the current modelling approach is not clear.

In addition to the complexity of relationships between natural enemies, pests, and crop production, future changes in climate will also have important effects on rainfall, temperatures, and humidity, which themselves will also impact crop health and production. The current lack of incorporation of these types of changes in the modelling also makes it difficult to interpret what impacts to food/feed future BD changes could have.

The ratio of natural enemies to pests is an interesting and potentially useful variable to model, but it's appropriateness also depends on whether this is a common variable that was measured for pest regulation in the papers that contributed to the meta-analysis. If different or varied measure of pest regulation were present in those papers, then the appropriateness of using the resulting pest regulation BES relationship to model future changes in pest regulation would be in question.

Equating changes in pests and crop production to food security is somewhat tenuous at best and I think more exact wording here is necessary. Food security is a complex term with six dimensions (availability, accessibility, stability, sustainability, utilization, and agency) and in most cases, decreased food security is not a function of a lack of food abundance. Instead, it is often caused by income inequality or political instability. More careful wording here is important.

Thanks for these good points; we agree that pest regulation and food system outcomes are influenced by multiple interacting ecological, climatic, and socio-economic factors that are not fully represented in our modelling approach. In response, we have revised the manuscript to clarify the scope, assumptions, and interpretation of our projections.

First, we have revised the framing of this analysis throughout the manuscript to ensure that it is not presented as a direct model of food security or crop yield. Instead, we now explicitly describe our approach as projecting biodiversity-mediated changes in biological pest regulation potential, using the ratio of natural enemies to pests as a mechanistic indicator of top-down control in agricultural systems. We clarify that this represents a key ecological regulating pathway underpinning agricultural production potential, rather than a direct estimate of food security outcomes. See lines 47-51, 308-329, 349-350.

> *"Our synthesis enables the forecasting of impacts and highlights how ecosystem services might be used to address future problems ahead of time. For instance, we forecast that biodiversity loss will reduce pest regulation in countries experiencing population growth and intensive development". Line 47-51.*

> *"We apply our modelling framework to a case study on agricultural pest regulation, a globally critical issue for food security. This focus aligns with the predominance of terrestrial data in our database and the growing attention on agricultural*

sustainability[52,53]. In particular, we examined the ratio of natural enemies to pests, a widely used proxy for top-down biological control across heterogeneous ecological systems[52–54]. Top-down biological control of agricultural pests by natural enemies supports crop production through the regulation of pest populations and is recognised as a key regulating Nature's Contribution to People (NCP) in global assessments by IPBES[11]. Accordingly, changes in predator-pest dynamics represent an important mechanism through which biodiversity influences agricultural production under global environmental change. To link our BES model with large-scale projections of biodiversity, we scaled BES data to Biodiversity Intactness Index (BII)[55] data to estimate future changes in the ratio of natural enemies to pests and associated pest control services. The BII projections capture biodiversity responses to anthropogenic pressures, primarily land-use change, under different socio-economic scenarios. Accordingly, projected regional differences primarily reflect variation in land-use intensity, biodiversity intactness, and socioeconomic vulnerability embedded within SSP scenarios, rather than direct climate effects on pest or predator dynamics. We find that regions with rapid population growth are projected to experience greater pressure on agricultural systems and stronger declines in biodiversity-mediated pest regulation" Lines 309-329.

"These results suggest that biodiversity loss may weaken an important ecological pathway underpinning agricultural production". Line 349-350.

Second, we explicitly acknowledge in our methods that our framework does not incorporate important mediating factors such as landscape configuration, crop type, management practices, or climate-driven changes in crop productivity. We have added text stating that our projections should therefore be interpreted as biodiversity-only effects on a biotic regulatory pathway, rather than a full agricultural system or yield model. See lines 773-778.

<u>"Assumptions and limitations of predictive modelling</u>
This predictive framework does not explicitly represent key mediating factors known to influence pest regulation outcomes, including landscape configuration, crop type, agricultural management practices, or climate-driven changes in crop productivity. The model therefore represents biodiversity-only effects on a biotic regulatory pathway rather than a full agricultural yield or food system model." Lines 773-778.

We believe these revisions improve conceptual clarity and ensure that the predictive analysis is appropriately framed as a biodiversity-driven projection of ecological regulation pathways rather than a socio-economic forecast of food security outcomes.

(4) There is understandably a very large number of BES papers present in the current literature and it would not be logistically possible to include all of them in a meta-analysis. However, the current methods describe choosing "the most relevant results" within ecosystem type and ecoregion, and within ES categories, requires some additional description to ensure that a representative and unbiased sample of papers was used in the analysis. In particular, it would

be useful to know what specific criteria were used to determine the relevance of a paper, and whether that potentially introduced any bias with respect to the geographic representativeness or statistical significance of the papers that contributed to the meta-analysis

Thank you for this comment. We have now added more detail into the methods around the addition of the most-relevant studies into our database on lines 555-558, 569-577, 581-588. and paste it below for review.

"After first filtering the literature search results by ecosystem type and ecoregion. Within each stratum, records were ranked using the WoS relevance algorithm (based on keyword matching rather than citation counts), and the top 100 studies were selected for screening." Lines 555-558.

> *"When adding studies to our database, study relevance was not determined based on study outcomes or statistical significance but strictly based on predefined inclusion criteria. Studies were included if they (i) explicitly quantified biodiversity (e.g. species richness, diversity indices, functional or phylogenetic diversity) and (ii) reported empirical relationships with at least one ecosystem function or service metric. Studies were excluded if they were purely theoretical, did not include quantitative biodiversity measures, or did not report a direct biodiversity–ecosystem function/service relationship. Where multiple results were available within a study (e.g. across ecosystem types, regions, or services), all eligible effect sizes that met the inclusion criteria were extracted (**Fig. S3**)". Lines 569-577.*

> *"Our literature sorting was intended to be blind with respect to geographic biases, but we acknowledge that, given the geographic biases shown in **Fig.1**, these may have crept into our analysis. We suggest, however, that the distribution of data most likely represents the underlying biases in funding and research into BES and BEF relationships. For example, it is quite clear from **Fig. 1** that there is a bias towards Europe, and particularly Germany. We think it is unlikely that there is a reason that Germany should be highlighted in by our search terms, but a number of key experiments (e.g., Jena, BIODEPTH[59,60]) ". Lines 581-588.*

We also include a funnel plot in Fig. S3, we paste it below for convenience.

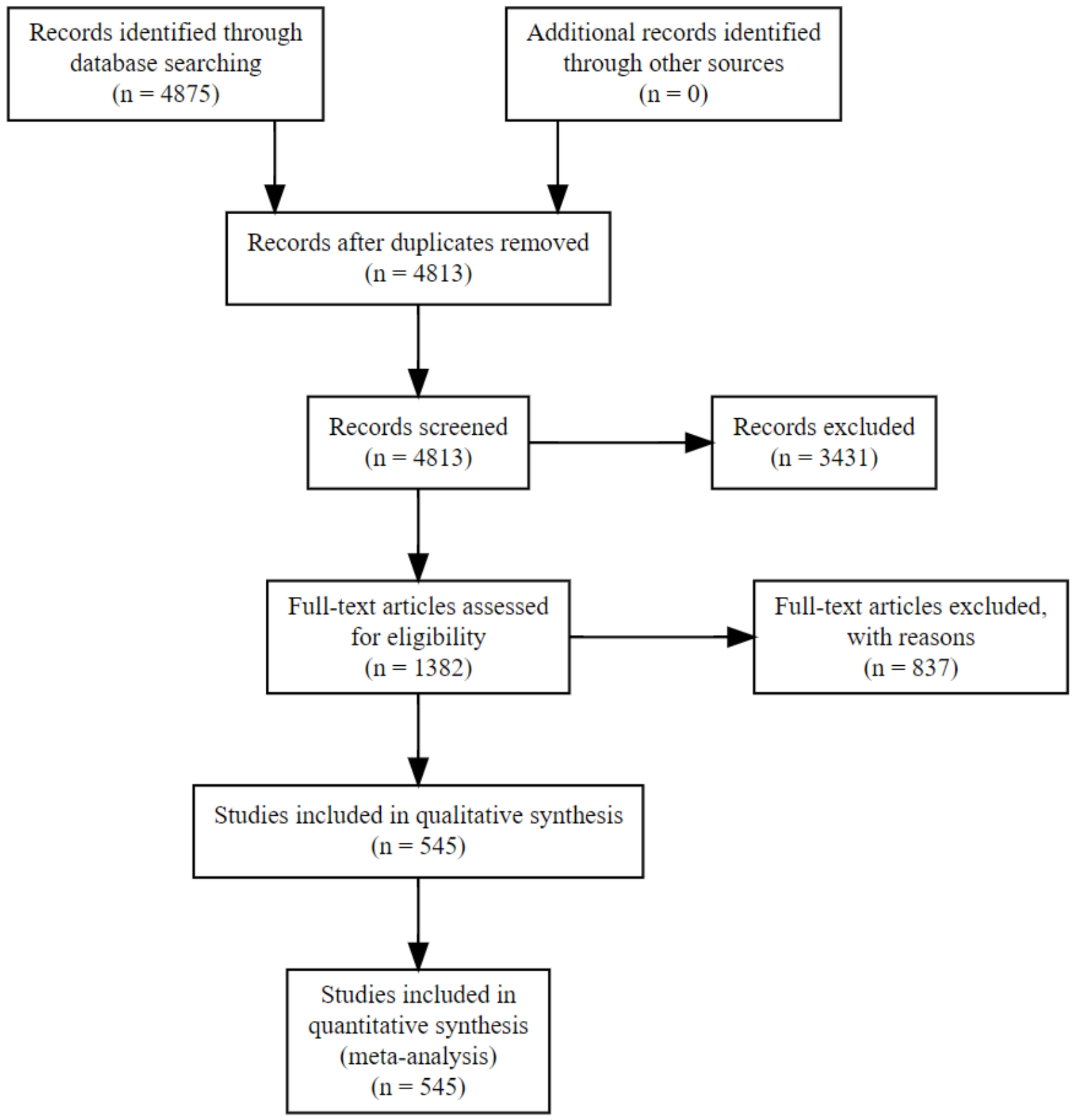

Figure S3. Summary of the study selection process for our stratified meta-analysis. *Papers were excluded if they were not relevant to our project, were based on simulated data, were review articles, or could not be accessed. At the full-text stage, studies were further excluded if no biodiversity-ecosystem dataset was available or if the ecosystem service or biodiversity metrics were unclear.*

Reviewer #2 (Remarks to the Author):

The paper "From oceans to forests, biodiversity powers climate-regulating services" is a valuable contribution to global ecology through the largest and most comprehensive synthesis of biodiversity-ecosystem service relationships to date. The authors aim to resolve longstanding debates about whether biodiversity contributions to ES are typically linear or saturating, demonstrating that both are common and context-dependent. The paper also has substantial policy relevance and represents a significant advance in quantifying and predicting biodiversity-ecosystem service relationships at a global scale with direct implications for conservation policy and climate mitigation strategies. I believe this thorough meta-analysis is likely to be well-cited. I have the following critiques and queries:

Introduction/Framing

The title (“From oceans to forests, biodiversity powers climate-regulating services”) implies the focus of the work was on climate-regulating services and did not consider others, but the meta-analysis covers all services. Perhaps the title could be more general (something like “From oceans to forests, biodiversity underpins ecosystem services”), with the abstract used to emphasise the important findings surrounding climate-regulating services?

I also wonder if ocean C should be the focus when “Oceanic carbon sequestration was especially underrepresented (n = 7), with far fewer datasets in our database than for terrestrial carbon sequestration (n = 154)” Perhaps focus on carbon in general (as terrestrial carbon also had a high effect size). This could then speak to all carbon sequestration initiatives and help challenge the paradigm of fast-growing monocultures for C credits.

Thank you for this, and we agree that the title should be more reflective of the manuscript. We have changed it to “Biodiversity safeguards ecosystem services and functions worldwide” as a result.

Literature sampling

The methods state: “To reduce the number of papers while ensuring a representative spread of data, we used a stratified survey method. After first filtering the literature search results by ecosystem type and ecoregion, we took the 100 most relevant results from each ecosystem type and ecoregion, resulting in 3875 papers.” How were the “most relevant” papers selected? What was the criteria?

In relation to this the authors state (Line 227) “Existing literature is geographically biased toward developed countries (Fig. 1A), underrepresenting regions critical for global food production, like South America and Africa44. To address this gap, we developed a novel heuristic modelling framework that, for the first time, links global BES relationships with projected climate and land-use change”. This had me wonder why this bias was not reduced by specifically sampling from different regions (as the authors did for ecosystem type)?

We agree that the term “most relevant” ambiguous and did not sufficiently explain how studies were selected. We have now clarified this in the Methods on lines 555- 558, 569-577,581-588. We have addressed this in response to R1 above, but we give the information here again for clarity.

Specifically, the “most relevant” papers were identified using the default relevance-ranking algorithm within the Web of Science (WoS) search engine. After filtering search results by ecosystem type and ecoregion, we retained the top 100 records returned under the WoS “Relevance” ranking for each category. This ranking is determined by the Web of Science search algorithm based on factors such as keyword matching, title, abstract, and citation metadata.

We have revised the Methods section to state this explicitly:

> *"After first filtering the literature search results by ecosystem type and ecoregion. Within each stratum, records were ranked using the WoS relevance algorithm (based on keyword matching rather than citation counts), and the top 100 studies were selected for screening." Lines 555-558.*
>
> *"When adding studies to our database, study relevance was not determined based on study outcomes or statistical significance but strictly based on predefined inclusion criteria. Studies were included if they (i) explicitly quantified biodiversity (e.g. species richness, diversity indices, functional or phylogenetic diversity) and (ii) reported empirical relationships with at least one ecosystem function or service metric. Studies were excluded if they were purely theoretical, did not include quantitative biodiversity measures, or did not report a direct biodiversity–ecosystem function/service relationship. Where multiple results were available within a study (e.g. across ecosystem types, regions, or services), all eligible effect sizes that met the inclusion criteria were extracted (**Fig. S3**)". Lines 569-577.*
>
> *"Our literature sorting was intended to be blind with respect to geographic biases, but we acknowledge that, given the geographic biases shown in **Fig.1**, these may have crept into our analysis. We suggest, however, that the distribution of data most likely represents the underlying biases in funding and research into BES and BEF relationships. For example, it is quite clear from **Fig. 1** that there is a bias towards Europe, and particularly Germany. We think it is unlikely that there is a reason that Germany should be highlighted in by our search terms, but a number of key experiments (e.g., Jena, BIODEPTH[59,60]) ". Lines 581-588.*

The role of biodiversity measures

I understand that you compiled studies that used different measures of biodiversity. As the studies use diverse metrics (e.g., species richness, evenness, functional and phylogenetic diversity), among other differences, so these data were rescaled. This is definitely a sensible approach, for the main analysis. However, I wonder if these different measures of biodiversity affected the findings, particularly the nature of the relationship (linear etc) between biodiversity and services – phylogenetic diversity is very different to species richness. Were these different measures relatively evenly represented across each service type? Were the different relationships (linear, saturating) over-represented by a particular biodiversity measure?

*Thanks for this very good suggestion. We now include, as an extended data figure (**Extended Data Figure 4**, page 20), a brief overview of how biodiversity measurement may affect the strength of relationships. Unfortunately, however, because we have more data on species richness as a metric compared to others in our database, we feel we do not have the statistical power to make strong claims about metric differences. We include our current findings in the supplement, including statistical significance tests, though, because we agree with the reviewer that this is an interesting path to consider.*

Where we have data on functional and phylogenetic metrics, we find they were disproportionately associated with saturating relationships, whereas taxonomic diversity was roughly evenly represented across relationship types. This suggests that the choice of biodiversity metric can partly influence the observed functional form of BES relationships, highlighting that some variation in reported linear vs. saturating patterns reflects methodological differences in addition to ecological processes. Nevertheless, because all metric classes were represented across multiple ecosystem services, the main conclusions of our study remain robust.

We draw the reader's attention to this in the main text:

> *"Variation in biodiversity-ecosystem service relationships partly reflects methodological choices as biodiversity metric classes differed among ecosystem services (Cramér's V = 0.34), and the form of relationships varied with metric type, with functional and phylogenetic diversity metrics more often producing saturating responses whereas taxonomic diversity evenly distributed across linear and saturating models (**Extended Data Fig. 4**)." Lines 118-124.*
>
> *"Variation in biodiversity metrics and relationship forms*
>
> *To assess how methodological choices vary across ecosystem service and ecosystem function categories, we analysed both the distribution of biodiversity metric classes and the form of fitted biodiversity-ecosystem relationships. Biodiversity metrics were grouped into four classes (taxonomic, functional, phylogenetic, and other). Our literature search was designed to capture broad biodiversity-ecosystem relationships rather than systematically sample biodiversity metric types; as such, these analyses should be interpreted as descriptive patterns within the compiled literature rather than unbiased estimates of metric usage across the field.*
>
> *First, we tested whether the distribution of biodiversity metric classes differed among ecosystem service and ecosystem function categories using a chi-squared test of independence, based on the contingency table shown in Extended Data Fig. 4. Because several cells had low expected frequencies, significance was assessed using Monte Carlo simulation (9,999 permutations). Effect size of the χ^2 test was quantified using Cramér's V (analogously to our use of Fisher's Z in analyses above), and standardised residuals were used to identify over- and under-represented combinations.*

We then examined whether best-fitting relationship form differed among biodiversity metric classes. For each observation, the best-fitting model (linear, logarithmic, or quadratic) was selected based on the highest Akaike weight. A contingency table of biodiversity metric class versus model type was analysed using a χ^2 test, and standardised residuals were used to identify deviations from expected frequencies." Lines 649-668.

Here is our extended data figure four, and the accompanying text on lines 118-124.

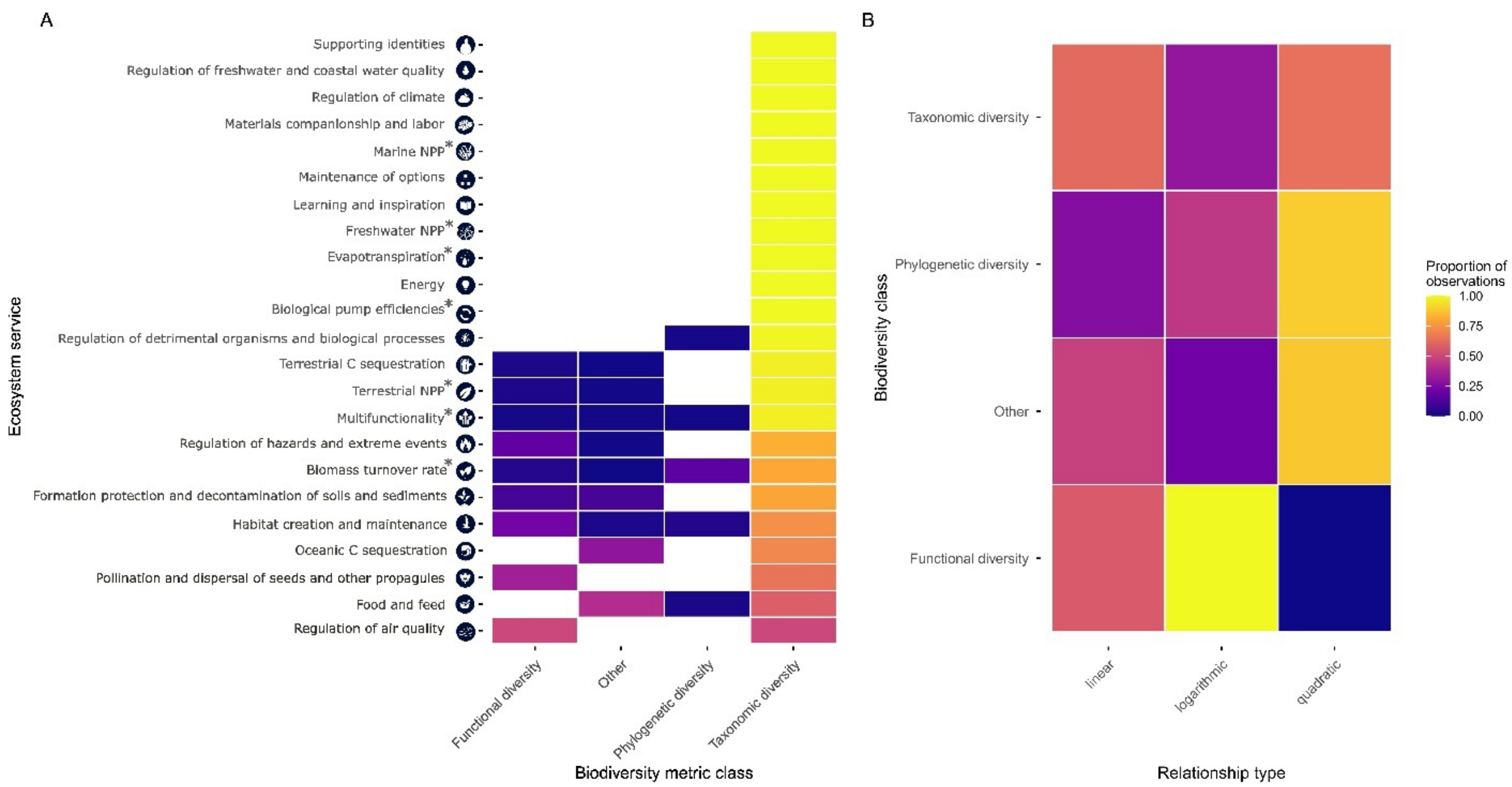

Extended Data Figure 4. Patterns of biodiversity metrics and reported relationships across ecosystem services and functions. *Heatmaps show the distribution of observations across (A) biodiversity metric classes versus ecosystem services and functions, and (B) biodiversity metric classes versus reported relationship types. Colour intensity indicates the proportion of observations within each category, with yellow and orange representing higher proportions. The distribution of biodiversity metrics differed significantly among ecosystem service categories ($\chi^{2^2} = 78.385$, Monte Carlo $p < 0.001$; Cramér's $V = 0.34$), with taxonomic diversity metrics common in our database. Biodiversity-ecosystem service and biodiversity ecosystem-function forms also varied among biodiversity metrics ($\chi^2 = 23.17$, $df = 6$, $p < 0.001$), with functional and phylogenetic diversity metrics showing an over-representation of saturating relationships, whereas taxonomic relationships were equally represented across model types. Response variables marked with an asterisk (*) are classified as ecosystem functions (EF), all other response variables are ecosystem services (ES).*

Finally, while checking the search terms to assess potential bias in biodiversity metrics, we noticed we made a typo in the definition of the search term in the manuscript. We apologise, and have now corrected this:

> We downloaded biodiversity-ecosystem service and biodiversity-ecosystem function literature from the Web Of Science using the following search string: TS = ("ecosystem service*" OR "ecosystem function*" OR ("ecosystem stability" AND "biodiversity") OR "species richness" OR "species diversity"). Given the diversity of metrics and terminology used in this field, relevant studies may not always be captured by strictly nested search strings. All records were therefore screened to ensure that only studies explicitly linking biodiversity to ecosystem services or functions were retained. *Lines 544-550.*

Ecosystem services are conflated with food production

A significant concern is that the food and feed provisioning forecast conflates ecosystem service provision with actual agricultural output. The paper models the decline in natural pest control services under biodiversity loss but presents this as predicting reduced food production, which is problematic because modern industrial agriculture has substantially decoupled food output from natural ES through chemical pesticides, crop intensification, and technological innovation. Whilst the paper correctly identifies that natural enemies provide biological pest control, this figure likely applies primarily to low-input or organic systems rather than global agriculture as a whole. Critically, countries with high human development index values and intensive agricultural development often produce more food despite lower biodiversity, because they can afford pesticide and fertiliser inputs. The forecast therefore predicts ES loss rather than food production loss, and the link to food insecurity depends on socio-economic factors (poverty, market access, farmer access to inputs) that the model does not capture. A more defensible interpretation would frame the finding as a loss of natural pest control services that creates dependency on chemical inputs, with implications for environmental sustainability and long-term agricultural resilience, rather than a direct threat to food production. The paper should clarify this distinction, acknowledge how agricultural intensification can compensate for ES loss, and contextualise the forecast to specific agricultural systems where natural pest control remains critical.

We thank the reviewer for this important point. We agree that our original wording did not clearly distinguish between biological pest regulation as an ecosystem service and realised agricultural production outcomes, which are additionally influenced by socioeconomic, technological, and management factors. Reviewer 1 raised similar concerns and, while we have addressed them above, we address them here as well for clarity.

In response, we have revised and reframed this section throughout the manuscript to clarify that our modelling framework focuses on biodiversity-mediated changes in biological pest regulation,

specifically through natural enemy–pest dynamics. This classification follows the IPBES framework, in which biological pest regulation is recognised as a regulating Nature's Contribution to People linked to food and feed provision.

We now consistently refer to projected changes in pest regulation services and their potential implications for agricultural systems and food security, rather than implying direct prediction of crop yields or food production. We have also clarified this distinction in the Abstract and Main text 49-51, 298-311, 349-353.

> *"For instance, we forecast that biodiversity loss will reduce pest regulation that underpins agricultural production, with potential implications for food production in countries experiencing population growth and intensive development"* (Lines 49-51, Abstract)

> *"We apply our modelling framework to a case study on agricultural pest regulation, a globally critical issue for food security. This focus aligns with the predominance of terrestrial data in our database and the growing attention on agricultural sustainability[52,53]. In particular, we examined the ratio of natural enemies to pests, a widely used proxy for top-down biological control across heterogeneous ecological systems[52–54]. Top-down biological control of agricultural pests by natural enemies supports crop production through the regulation of pest populations and is recognised as a key regulating Nature's Contribution to People (NCP) in global assessments by IPBES[11]. Accordingly, changes in predator-pest dynamics represent an important mechanism through which biodiversity influences agricultural production under global environmental change." (Lines 308-319, Main)*

> *"Top-down biological control of agricultural pests by natural enemies supports crop production through the regulation of pest populations and is recognised as a key regulating Nature's Contribution to People (NCP) in global assessments by IPBES[11]. Accordingly, changes in predator-pest dynamics represent an important mechanism through which biodiversity influences agricultural production under global environmental change." (Lines 315-319, Main)*

> *"These results suggest that biodiversity loss may weaken an important ecological pathway underpinning agricultural production, particularly in regions facing high demand for food systems. By contrast, countries with higher HDI values are predicted to experience comparatively greater levels of biological pest regulation potential, reflecting potential gains for agricultural systems." (Lines 349-353, Main).*

We have also re-labelled figure 4:

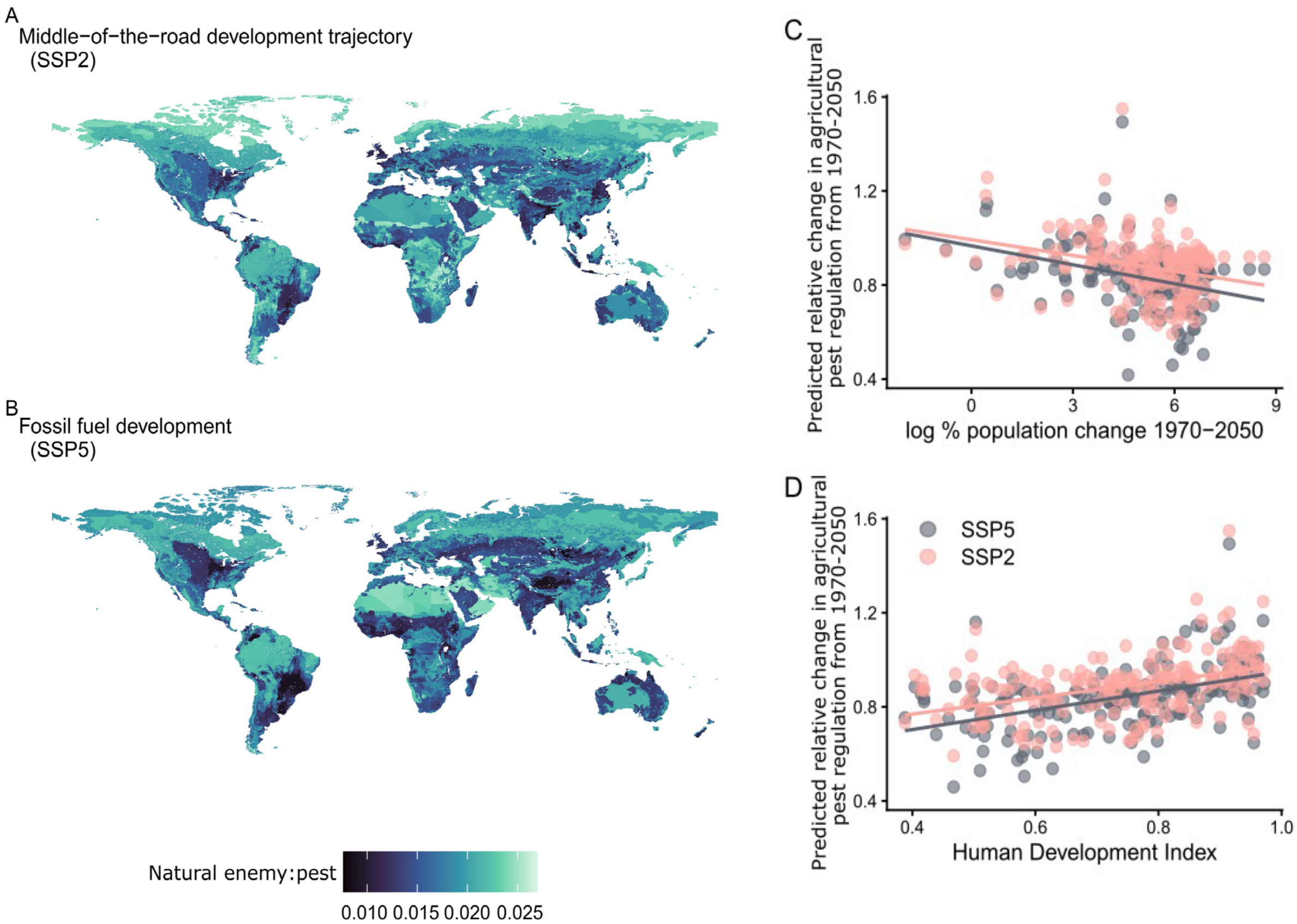

Figure 4: Forecasting ecosystem services helps in planning to meet future human demands. *Here we combine our BES data with two IPCC shared socio-political climate-change scenarios (SSPs)[45] and their resulting biodiversity intactness values[46] under 'middle of the road' (A; SSP2) and 'fossil-fuel development' (B; SSP5) scenarios (see Figure S2 for all scenarios). While the differences between these two scenarios for biodiversity are complex, our model forecasts that under each scenario, there will be subtly different degrees of protection provided from crops by natural pathogens (A and B). A decrease in natural protection from pests would be expected to make agricultural production more difficult, and these changes will disproportionately affect countries experiencing greater population growth and with low human development index values (C, D). We found that pest protection is reduced under fossil-fuel–driven development (SSP5; $\beta = -0.0488 \pm 0.0105$, $z = -4.65$, $p < 0.001$), and we observe a disproportionately greater decline in faster-growing countries ($\beta = -0.0088 \pm 0.0021$, $z = -4.28$, $p < 0.001$;* ***Table S2****). Pest protection is also reduced in countries with low human development index values ($\beta = 0.176 \pm 0.038$, $z = 4.59$, $p < 0.001$) which is further exacerbated under fossil fuel development ($\beta = -0.048 \pm 0.0105$, $z = -4.59$, $p < 0.001$;* ***Table S3****). This case study*

highlights the importance of considering not only forecasting climate or biodiversity, but also ecosystem services when considering future decision-making.

Climate scenarios

The methods describe 5 IPCC climate change scenarios and a monte carlo sampling method to propagate uncertainty but only 2 scenarios are shown in the results (main text or supplementary) and I cannot find any spatial depiction/description of this uncertainty. Additionally, what GCMs are used in the climate scenarios selected? Are they ensemble averages?

We thank the reviewer for this important comment. First, we now include results for all five Shared Socioeconomic Pathways (SSP1-SSP5) in the Supplementary Information (Fig. S2). We have retained SSP2 and SSP5 in the main manuscript for clarity and to illustrate the range between a "middle-of-the-road" and high-impact development pathway, but we now refer to all pathways and Fig. S2 in the caption of Figure 4.

Second, we now provide a more complete representation of uncertainty. We include 95% credibility intervals derived from the Monte Carlo propagation procedure for all scenarios, and we have added spatially explicit uncertainty maps in the Supplementary Information (Fig. S2). These allow readers to assess how uncertainty varies geographically and across ecosystem service projections, rather than relying solely on global summary values.

Finally, we clarify the structure of the modelling framework. In our models, future biodiversity change is represented using the Biodiversity Intactness Index (BII) from the PREDICTS framework, which is driven by land-use change and associated anthropogenic pressures under the SSP scenarios. The SSPs themselves provide socioeconomic pathways that indirectly embed climate policy and emissions trajectories, but the biodiversity projections used here are not climate-forced and are derived independently of GCM outputs.

We have revised the Methods and Discussion to make this modelling hierarchy explicit and to clarify the distinction between socioeconomic scenarios (SSPs), land-use driven biodiversity change (BII), and ecosystem service responses. Relevant changes are provided on lines 302-305, 322-329, 363-366, 758-766.

> *"By integrating socioeconomic scenario-based biodiversity projections from the Biodiversity Intactness Index (BII; derived under the Intergovernmental Panel on Climate Change (IPCC) Shared Socioeconomic Pathways)[50,51]." Lines 302-305.*
>
> *"The BII projections capture biodiversity responses to anthropogenic pressures, primarily land-use change, under different socio-economic scenarios. Accordingly, projected regional differences primarily reflect variation in land-use intensity, biodiversity intactness, and socioeconomic vulnerability embedded within SSP scenarios, rather than direct climate effects on pest or predator dynamics. Regions with rapid population*

growth are projected to experience greater pressure on agricultural systems and stronger declines in biodiversity-mediated pest regulation because increasing food demand and land-use conversion coincide with greater reliance on local ecosystem services." Lines 322-329.

"By combining this synthesis with a heuristic modelling framework, we predict how biodiversity loss may alter key ecosystem functions and services under future socioeconomic and land-use scenarios, identifying regions and services most at risk." Lines 363-366.

"To visualise spatial uncertainty in predicted ecosystem services, Monte Carlo simulations for each combination of ecosystem service type, ecoregion, year, and climate scenario were compiled into raster stacks. From these stacks, we calculated 95% credible interval (a Bayesian measure of uncertainty around estimated values) at each grid cell as the 2.5th and 97.5th percentiles of the posterior predictive distribution. These intervals were mapped alongside mean predicted ecosystem service values to produce spatially explicit projections with associated uncertainty. This approach allows comparison of both expected values and uncertainty across ecoregions and climate scenarios, providing a robust and interpretable representation of projected biodiversity-ecosystem dynamics." Lines 758-766..

We also copy Figure S2 below for review.

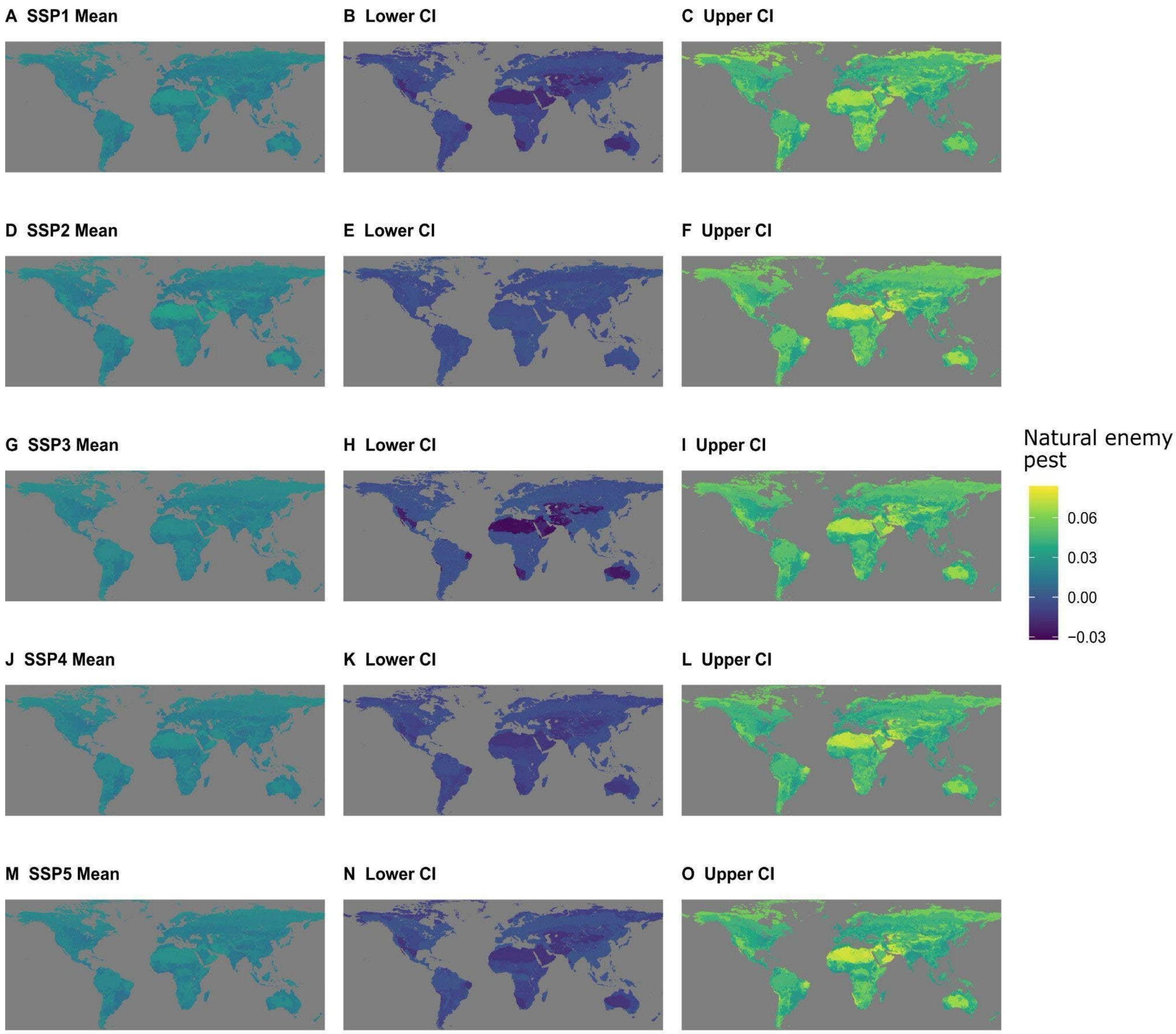

Figure S2. Global projections of natural enemy–pest ratios under future socio-economic scenarios. *Spatial patterns of the natural enemy to pest ratio are shown for five Shared Socioeconomic Pathways (SSP1-SSP5) in 2050. Rows correspond to scenarios, and columns show the mean projection and associated 95% credibility intervals (lower and upper bounds). Colours indicate the magnitude of the ratio, with a common scale applied across panels to enable comparison among scenarios and uncertainty estimates. Low natural enemy-pest ratios indicate reduced biological pest control capacity and higher potential pest pressure. Projections show spatially heterogeneous biocontrol potential, with scenario-dependent shifts in magnitude and regionally variable uncertainty, generally indicating lower ratios under higher-pressure SSPs.*

Minor comments
Line 48 – BES acronym not previously defined

Thank you for noticing this, we now define terms prior to using them in the text.

> *"While the consequence of biodiversity declines on ecosystem functioning is well-established, the functional form and scalability of biodiversity-ecosystem service (BES) and biodiversity-ecosystem function (BEF) relationships (hereafter, biodiversity-ecosystem relationships) remain a fundamental challenge." Lines 76- 80.*

Figures seem to have a spelling error "compansionship"

Thank you for noticing this spelling error: we have corrected the plots.

Lines 108 -109 "No ES can rise indefinitely with biodiversity, but our data suggest saturation limits have rarely been reached, implying functional redundancy may be overestimated". This has rarely been reached in the experiments? Surely it has been reached in reality? This should be clarified.

As suggested, we now explicitly clarify that situation does occur and add an example to support this - see lines 154-155.

> *"However, in some systems saturation is reached relatively quickly, such as decomposition in freshwater systems which may saturate at low levels of biodiversity[28]."*

234-246 a whole paragraph focused on methods within the results/discussion
Whilst we appreciate this point, we feel that some exposition of the methods is needed in the prediction section to retain the narrative flow of the manuscript, so we would prefer to retain it as it stands, if possible.

We feel that reducing this information may make the flow of ideas difficult to follow.

Extended data figure 3. Terrestrial, marine, freshwater and estuarine should be aligned across graphs (even if there's a gap in the case where a category is missing) for easier interpretation
Thank you for the suggestion, we have now aligned the categories on the plot as suggested and agree that it is easier to follow patterns with this change. We copy the plot below for review.

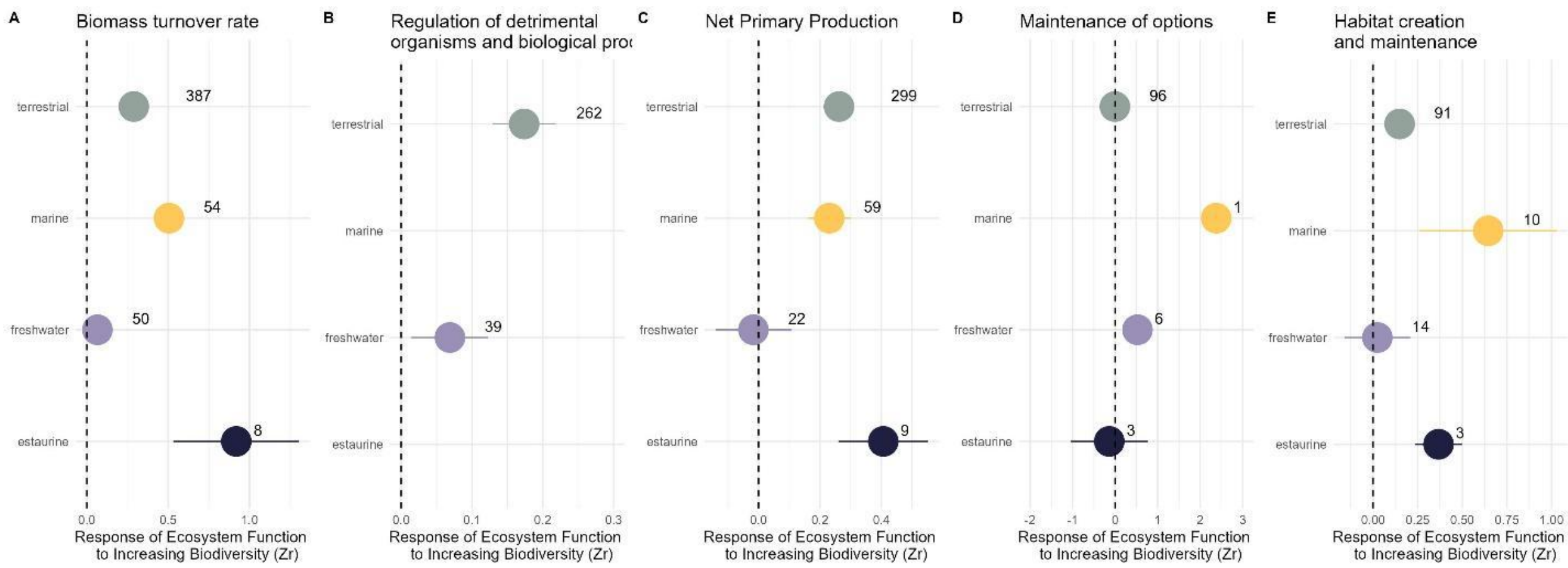

Extended Data Figure 3: Biodiversity and ecosystem service effect sizes vary across ecosystem types. *Data are shown for our database's five most common ecosystem service categories. Effect size scores (measured with Fisher's Z-score; Zr) are averaged (±1 SE) across datasets within each ecosystem service category and ecosystem type, where a high effect size value indicates a stronger effect of biodiversity on ecosystem service compared to a lower value. Dataset n values are shown beside the average effect size value. Overall, our database has high dataset n values for terrestrial studies and low n values for estuarine studies, and this was consistent across all ecosystem service categories. Datasets from marine and freshwater ecosystems were not as common as those from terrestrial studies, and for some ecosystem services (regulation of detrimental organisms and biological processes, maintenance of options), data from these ecosystem types were scarce or missing.*

Reviewer #3 (Remarks to the Author):

This manuscript is a substantial synthesis of the biodiversity literature in terrestrial, aquatic and marine ecosystems and draws conclusions about how much biodiversity is necessary to maintain certain types of ecosystem services. The authors forecast what biodiversity loss is expected with 2 different climate change scenarios and what that will mean for food security in the future.

This manuscript is fairly well-written and will make a good contribution to the biodiversity literature with some revisions for clarity. I have the following suggestions to improve the manuscript:

1) The discussion of the different analyses of effect sizes vs which services were more or less sensitive to biodiversity were slightly confusing (Results in Figure 2 vs Figure 3). The authors

point out that regulating services including hazards were typically a linear relationship, (lines 95-96), but then the authors say that hazard regulation was insensitive to biodiversity (lines 145-146). It would be helpful to discuss the nuances of these findings and how different analyses led to somewhat different conclusions about hazards and biodiversity. What do these two different findings mean and how are they distinct and useful in and of themselves but also when put together?

"We thank the reviewer for highlighting this point and agree that our original wording did not sufficiently distinguish between the two analyses. We have revised the text on lines 198-199 to clarify that the two approaches quantify different aspects of biodiversity-ecosystem service relationships.

The functional form analysis (Fig. 2) examines the shape of the relationship between biodiversity and ecosystem service delivery. For hazard regulation, relationships were frequently classified as linear, meaning that changes in biodiversity were associated with proportional changes in service provision across the observed range. However, the effect-size analysis (Fig. 3) evaluates the overall magnitude or strength of biodiversity dependence. In the case of hazard regulation, although relationships were often linear, the slopes were generally very small, meaning that biodiversity had only a weak overall effect on service delivery. In practice, this corresponds to relationships that are technically linear but essentially close to flat. We also integrated this clarification into the surrounding discussion of flooding and erosion regulation so that readers can better understand how these findings are complementary rather than contradictory.

To make this distinction clearer, we revised the text to state:

> *"Oceanic carbon sequestration, which is a significant part of the global carbon cycle, exhibits by far the strongest positive response to of any service or function category[34]. This strong sensitivity may reflect the strong biological control of marine carbon cycling, including phytoplankton productivity, microbial loop dynamics, and the biological carbon pump, all of which are closely linked to community composition and trophic structure. However, this result should be interpreted cautiously, as oceanic carbon sequestration is relatively under-represented (n = 7), with far fewer datasets in our database than for terrestrial carbon sequestration (n = 154), highlighting a critical gap in current knowledge (**Fig. 3**). This exceptional sensitivity implies that blue carbon sinks are both vulnerable to biodiversity loss and dependent on sustaining diverse communities[35,36]. Maximising the efficacy and long-term viability of blue carbon strategies will require moving beyond habitat preservation toward explicitly incorporating biodiversity as a key component[37,38]. Conversely, some services, like hazard regulation, exhibited weak overall sensitivity to biodiversity in the effect-size analysis, despite often*

showing linear relationships in the functional form analysis (Zr = 0, r^2 = 0.17; ***Fig. 2, 3, Fig. S1****).” Lines 182-196.*

2) There are some terms that need to be explained briefly/defined in the text because they need to be understood by the reader if the reader does not go read the full methods: spatial grain and Human Development Index. Even after reading the methods section, these terms were not immediately obvious and clear. Also, how/why they relate to biodiversity is not necessarily clear. For the HDI, what does a high vs a low HDI mean for people in that country? And how might this relate to biodiversity? Why did the authors select HDI to use in this analysis?

We agree that clearer definitions of key terms would improve accessibility, and we have now revised the main text to explicitly define both spatial grain and the Human Development Index at their first mention. See lines 333-342.

> *“Our projections suggest that biological pest regulation services relevant to food and feed production are reduced under fossil fuel development compared to the middle-of-the-road development trajectory, though the impacts are heterogeneous (**Fig. 4A, B, Fig. S2**) and particularly pronounced in regions experiencing rapid population growth and with low Human Development Index (HDI) values (**Fig. 4C, D; Table S2**). HDI is a composite measure of national development based on life expectancy, education, and income. Countries with low HDI typically experience higher reliance on local ecosystem services and more rapid population growth compared to high HDI countries. As such, HDI provides a useful proxy for socioeconomic vulnerability and dependence on biodiversity-mediated ecosystem services[56].” Lines 333-342.*

3) If the point of Extended Data Figure 2 is the better understand if experimental vs observational studies show the same relationship with biodiversity increases, it would be nice to color code the ones where the relationship is different depending on the study type, so it is more obvious for the reader which ones are different vs which ones show the same type of relationship no matter which kind of study was used.

Thank you for this. We have now annoted the different effect sizes on the figure, and we agree that it now makes it much clearer that there are very few (only 2) services that differ.

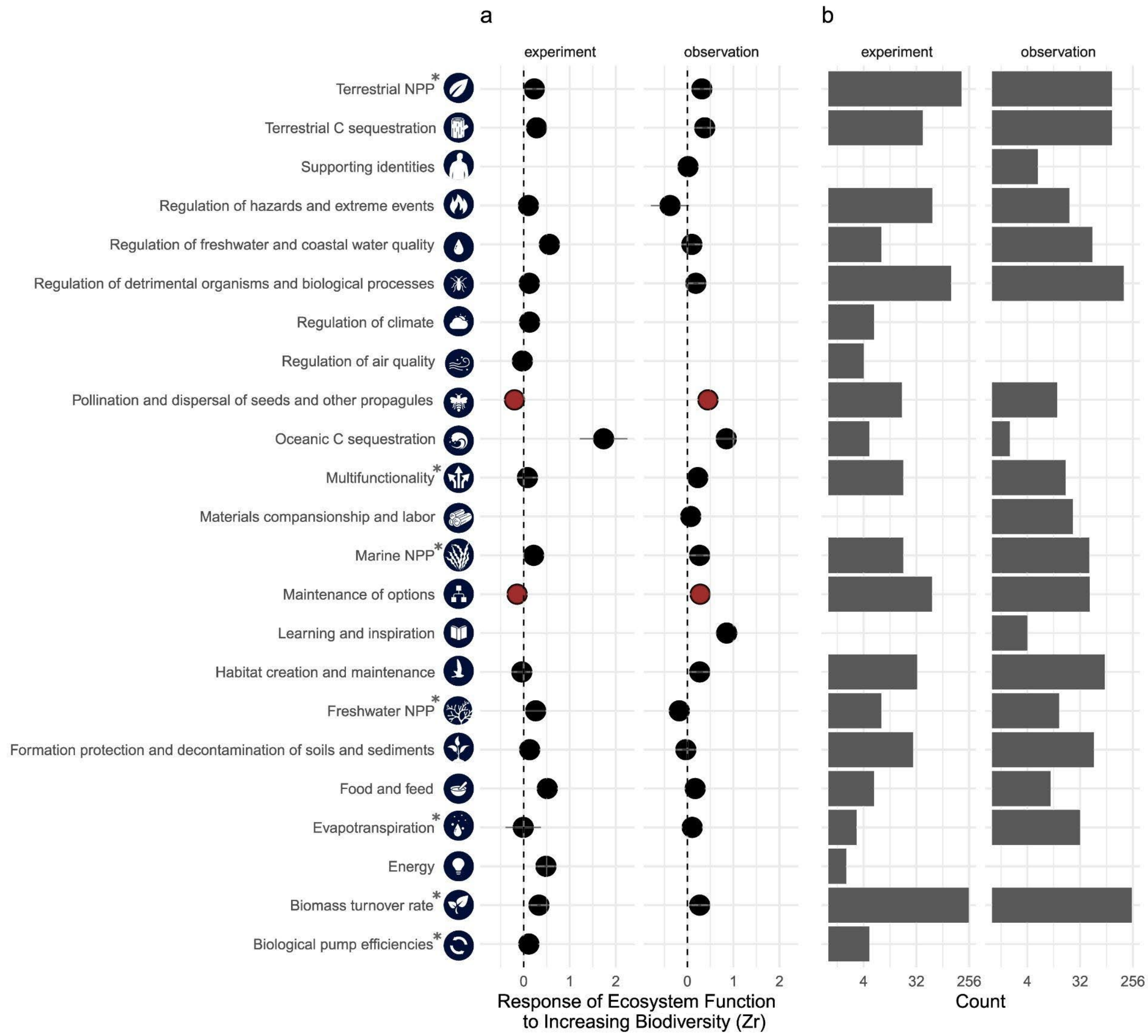

Extended Data Figure 2. *Comparison of biodiversity responses across ecosystem service categories, as effect size (Fisher's Z-score; Zr; Panel a) and the number of studies used to calculate effect size (Panel b). High effect size values indicate a stronger effect of biodiversity on ecosystem service compared to values. Data are split by study type. The effect sizes which are different (positive vs negative) between study types, are highlighted in red. Response variables marked with an asterisk (*) are classified as ecosystem functions (EF), all other response variables are ecosystem services (ES).*

4) The authors point out that sampling and design biases could be contributing to their findings (for example lines 199-200). It might be helpful to include a bit more discussion of what the authors think they can state unequivocally and also what might be due to a lack of studies in particular ecosystems or at particular spatial scales, etc. A bit more discussion of how to

interpret these findings would be helpful. I think these findings are very interesting and useful, and also would like to ensure that the results are taken within the context of what is still less studied and may change as we have more study results in the future.

Thank you for this comment. In regard to sampling and design bias we have added more detail into the methods materials to better describe the processes we used to include papers in our database. We addressed sampling biases in an earlier response to R2 but copy the relevant sections here for convenience.

> *"When adding studies to our database, study relevance was not determined based on study outcomes or statistical significance but strictly based on predefined inclusion criteria. Studies were included if they (i) explicitly quantified biodiversity (e.g. species richness, diversity indices, functional or phylogenetic diversity) and (ii) reported empirical relationships with at least one ecosystem function or service metric. Studies were excluded if they were purely theoretical, did not include quantitative biodiversity measures, or did not report a direct biodiversity–ecosystem function/service relationship. Where multiple results were available within a study (e.g. across ecosystem types, regions, or services), all eligible effect sizes that met the inclusion criteria were extracted (**Fig. S3**)". Lines 569-577*
>
> *"As our literature review method was not exhaustive, it was not designed to quantitatively measure biases in data collection across ecosystem type or ES. However, we would not expect the paper-selection processes to select certain systems over others, as data across regions and ecosystem type was explicitly considered. Our literature sorting was intended to be blind with respect to geographic biases, but we acknowledge that, given the geographic biases shown in **Fig.1**, these may have crept into our analysis. We suggest, however, that the distribution of data most likely represents the underlying biases in funding and research into BES and BEF relationships. For example, it is quite clear from **Fig. 1** that there is a bias towards Europe, and particularly Germany. We think it is unlikely that there is a reason that Germany should be highlighted in by our search terms, but a number of key experiments (e.g., Jena, BIODEPTH[59,60]) ". Lines 578-588.*

Thank you for the suggestion to investigate difference across ecosystems and spatial scales, to understand this we have added a contingency table into the supplement (Table S4) to support

the data shown in Figure 1, and we have added text to the discussion on lines 263-268 and copy the text below.

> *"These patterns should be interpreted in the context of uneven sampling across the global biodiversity-ecosystem literature, with strong representation of terrestrial systems and fine spatial grains, and limited coverage of freshwater and marine ecosystems and broader-scale studies (Table S4; Figure 1). While overall biodiversity effects are robust, some ecosystem differences and functional forms may shift as additional data become available from under-sampled systems and scales." Lines 263-268.*

5) The case study looking at future climate change and ecosystem services is an interesting idea. However, it would be useful to have a little more discussion of how the modeling effort works and also the results. Why are there the predicted differences in food security between different regions? What is driving those differences? Is it primarily climate change impact differences or human demographic differences or both? How do the human demographics interact with the climate change predictions? This is where having a better explanation of HDI above will hopefully also help with clarifying the results of the case study more as well. The case study right now feels as if it is not well enough explained to add as much value to the paper as I had hoped.

We agree that the case study would benefit from a clearer explanation of both the modelling framework and the interpretation of regional variation in projected outcomes. In the revised manuscript, we have expanded the description of the modelling approach to clarify how biodiversity-ecosystem service relationships were linked to projections of biodiversity change using the Biodiversity Intactness Index (BII) under shared socioeconomic pathways (SSPs). We also clarify that projected changes in ecosystem services emerge from the combined effects of biodiversity loss driven by land-use and climate-related pressures embedded in the SSP scenarios, and regional differences in socioeconomic context captured by HDI. Importantly, regional variation in outcomes is not only driven by differences in scenario inputs, but also reflects uncertainty in the underlying biodiversity–ecosystem service relationships estimated for each region; this uncertainty is explicitly quantified and propagated through the modelling framework to generate region-specific prediction intervals. We highlight the changed sections of the manuscript below.

> *"The BII projections capture biodiversity responses to anthropogenic pressures, primarily land-use change, under different socio-economic scenarios. Accordingly, projected regional differences primarily reflect variation in land-use intensity, biodiversity intactness, and socioeconomic vulnerability embedded within SSP scenarios, rather than direct climate effects on pests or predator dynamics. Regions with rapid population growth and lower HDI values are projected to experience greater pressure on agricultural systems and stronger declines in biodiversity-mediated pest regulation because increasing food demand and land-use conversion coincide with greater reliance on local ecosystem services." (Lines 321-329, Main)*

“To model predicted future changes to biodiversity, we used projected Biodiversity Intactness Index (BII) values from the PREDICTS project[55,65]. BII is the average abundance across a large and functionally diverse set of species relative to their reference populations, but does not consider climate change impact[51]. Specifically, we obtained rasterised (spatial resolution 0.25 degrees) predicted BII values (and country-level averaged BII values) for each of the five Shared Socioeconomic Pathways (SSPs)scenarios described by the IPCC[66,67]. These pathways include sustainable development (SSP1), middle of the road (SSP2), regional rivalry (SSP3), inequality (SSP4), and fossil fuel development (SSP5) and were taken for the following years: 1970, 2020, 2030, 2040, 2050. We chose these pathways as they represent the most realistic alternative socioeconomic development trajectories under future socio-economic development[66].” (Lines 700-710, Methods)

“Projected regional differences in ecosystem service outcomes emerge from the interaction between scenario-driven changes in biodiversity (via BII under SSPs), socioeconomic context (captured by HDI and population trajectories), and uncertainty in the underlying biodiversity–ecosystem service relationships, which is propagated through all projections”. (Lines 781-785, Methods)

Additional comments:

1) Do we have a sense for why the ocean carbon sequestration is so sensitive to biodiversity? (lines 138-145) And could this sensitivity be driven at all by the fact that there were only a few studies the authors found that dealt with ocean carbon sequestration?

We added some text into the manuscript to explain the strength of this relationship and that there were relatively few data points in the ecosystem service category on lines 188-194.

*“This strong sensitivity may reflect the strong biological control of marine carbon cycling, including phytoplankton productivity, microbial loop dynamics, and the biological carbon pump, all of which are closely linked to community composition and trophic structure. However, this result should be interpreted cautiously, as oceanic carbon sequestration is relatively under-represented (n = 7), with far fewer datasets in our database than for terrestrial carbon sequestration (n = 154), highlighting a critical gap in current knowledge (**Fig. 3**).” Lines 188-194.*

2) Lines 602: I would add in what a high HDI means… vs low HDI.

As requested, we have clarified the meaning of the Human Development Index (HDI) in the revised manuscript, see lines 338-342.

“HDI is a composite measure of national development based on life expectancy, education, and income. Countries with low HDI typically experience higher reliance on local ecosystem services and more rapid population growth compared to high HDI

countries. As such, HDI provides a useful proxy for socioeconomic vulnerability and dependence on biodiversity-mediated ecosystem services[56]*". Lines 338-342.*

Response to reviewers

We thank the editorial team and the reviewers for reviewing our manuscript. We have now integrated all suggestions, which have improved the manuscript throughout. We provide a point-by-point response to each of the reviewers' comments below, *in blue italics*.

Reviewer #1 (Remarks to the Author):

Thank you for the opportunity to review the revised manuscript "Biodiversity safeguards ecosystem services and functions worldwide." Overall, I think that the authors have done a good job addressing the reviewer comments, and in particular my comments, on the initial submission. While I do have some minor comments that I detail below, I congratulate the authors on their substantial and important work and look forward to seeing this paper in print.

1. L48-49: I'm not sure how forecasting future BD change impacts on ES highlights how ecosystem services might be used to address future problems ahead of time. However, I can see how this highlights the importance of conserving biodiversity for ecosystem services and the benefits they provide.

We thank the reviewer for this helpful comment. We agree that the original wording was ambiguous. We have revised the text to better reflect our intended meaning, emphasising instead that forecasting biodiversity-ecosystem service relationships can inform proactive responses to anticipated environmental change.

Revised text:

> *"Our new synthesis enables the forecasting of impacts, for example that biodiversity loss will reduce pest regulation in countries experiencing population growth and intensive development and highlights how ecosystem services might be used to address future problems ahead of time." (Lines 45-48).*

2. L123: I would probably not use "underpins" here. Pest regulation can be an important contribution to food and feed provision, but depending on the circumstances might be relatively unimportant to that ES.

Thank you for this suggestion. We have revised the wording to "may contribute to" to better reflect the variability in the importance of food and feed provision which may exist.

> *"Using this framework, we evaluate the functional forms of BES/BEF relationships, quantify their sensitivity to biodiversity loss, identify generalised factors shaping these patterns, and model future trajectories in pest regulation which may contribute to food and feed provision under alternative population growth and development scenarios." (Lines 94-96).*

3. L143: I would include a definition of what you consider "low-to-moderate r2 values" as this interpretation will likely differ across readers. You could include a range of values here.

Thank you for this suggestion. We agree that "low-to-moderate" requires a clearer definition. We have revised the text to define this range explicitly based on the observed distribution of R^2 *values across models.*

Revised text:

> *"Most biodiversity-ecosystem relationships showed low-to-moderate* r^2 *values (e.g.,* $r^2 < 0.4$*;* ***Fig. S1****), indicating that biodiversity is an important but context-dependent driver of ecosystem functioning and service delivery." (Lines 117-119).*

4. L155: Awkward wording with "...relationships and within relationships in well-studied categories..."
Thank you for highlighting this awkward phrasing. We have revised the sentence to remove redundancy.

Revised text:

> *"While uneven data distribution, particularly uneven representation across ecosystem types and spatial grain (****Fig. 1****), and across ecosystem service and ecosystem function categories may influence these findings, the observed variation in BES and BEF relationships, including within well-studied categories, suggests that some services follow linear relationships while others tend towards saturation." (Lines 126-129).*

5. L172: I'm a little unsure what this sentence is trying to say as a main point. Perhaps an example here would help clarify?

We thank the reviewer for this comment. We have revised the sentence to clarify and included an explicit example to improve interpretability. The updated text now makes clear that the reported values represent the approximate species richness at which biodiversity effects begin to saturate, with limited additional gains in ecosystem service or function beyond this threshold.

Revised text:

> *"For saturating relationships, the asymptote, where additional species no longer substantially increase ecosystem service or function, at a median of ~4 (± MAD = 2) species in freshwater, ~14 (±9) in terrestrial, and ~23 (±17) in marine ecosystems (****Extended Data Fig. 1****)." (Lines 145-147).*

L266: There seems to be a bit of contradiction between this sentence that states "biodiversity effects strengthened with increasing grain" and later on in the paragraph (L273-274) state that biodiversity contributions to a number of ES decline with increasing spatial grain.

Thank you for highlighting this lack of clarity. We have revised the text to clarify that the positive effect of spatial grain reflects the overall average relationship across all ecosystem services and functions, whereas individual services and functions exhibited contrasting responses, including significant declines with increasing spatial grain.

Revised text:

> *"The influence of spatial grain on BES and BEF was highly context dependent. Across all ecosystem services and functions, biodiversity effects strengthened with increasing grain ($\beta = 210.26$, $p < 0.001$; **Table S1**), indicating that biodiversity contributions to ecosystem services and functions become more apparent at broader spatial scales. This pattern may partly reflect a beta-diversity effect, whereby spatial turnover in species composition enhances functional complementarity across sites, amplifying aggregate ecosystem service delivery at coarser grains, as predicted by spatial insurance and complementarity theories. However, responses varied substantially among ecosystem services and functions." (Lines 217-224)*

7. L301-302: I would probably define here what you mean by fine spatial grain versus broader-scale studies. Readers might interpret these categories differently based on the type of research they are familiar with.

We thank the reviewer for this suggestion. We now clarify what we mean by fine spatial grain and broader-scale studies by providing examples of the spatial extents represented by these categories in the revised manuscript.

Revised text:

> *"These patterns should be interpreted in the context of uneven sampling across the global BES and BEF literature, with strong representation of terrestrial systems and fine spatial grains (e.g., quadrats, individual plots), and limited coverage of freshwater and marine ecosystems and broader-scale studies (e.g., landscapes, regions) (**Table S4; Figure 1)."** (Lines 245-249).*

8. L311: Weaker and negative?

Thank you for identifying this ambiguity. We have revised the text to clarify that the relationships for biomass turnover rate and regulation of hazards and extreme events were both weaker and negative.

Revised text:

> *"Effects were weaker and negative for biomass turnover rate ($\beta = -0.03$) and regulation of hazards and extreme events ($\beta = -0.13$), with no detectable differences for several other services, including evapotranspiration and food and feed (Table S1; Extended Data Fig. 2)." (Lines 258-260)*

9. L332: Awkward wording here with "to get closer to elucidate"

Thank you for highlighting this awkward phrasing. We have revised the sentence to improve clarity and readability by replacing "to get closer to elucidate" with "to elucidate".

Revised text:

> *"Future progress will rely on combining global syntheses with coordinated, cross-system experiments that explicitly test how biodiversity scales through space, time, and function. Priorities include creating hybrid experimental and observational networks that embed manipulations within long-term monitoring frameworks (e.g., plots scale manipulations on reef patches and survey data collected at both local and regional scales), hierarchical sampling designs to quantify α-, β-, and γ-diversity contributions to service delivery, and standardised experimentation across ecosystem types to elucidate mechanisms, generalities, and context-dependant differences." (Lines 267-274).*

10. L395: This sentence makes me wonder if your model is predicting just natural enemy numbers or the ratio between predators and pests? At L351 you state that you focus on the ratio of natural enemies to pests, but L395 specifically mentions natural enemy declines. I'm not sure these details are described in the methods section either. It would be beneficial to make clearer how changes in Biodiversity Intactness were translated into the predator:pest ratio, especially since a change in this ratio could happen due to changes in either predators or pests (or both).

We thank the reviewer for highlighting this point. Our projections are based on changes in the pest to predator ratio, not changes in natural enemy abundance alone. We agree that our original wording could be interpreted otherwise. We have therefore revised the main text to refer to changes in the predator ratio and have expanded the Methods to clarify how changes in Biodiversity Intactness are translated into changes in the predator ratio.

Revised text:

> *"In particular, we examined the ratio of natural enemies to pests, a widely used proxy for top-down biological control across heterogeneous ecological systems[53–55]. We forecast changes in this ratio as an integrated measure of biological pest regulation, rather than modelling changes in natural enemies or pest populations separately". (Lines 292-295)*
>
> *"Our model predicts reductions in the natural enemy to pest ratio under SSP5 relative to SSP2 in North America, South America, and Asia, whereas Australasia and Africa show comparatively minor differences between scenarios". (Lines 321-323)*
>
> *"Predictions for future ES values were obtained by inputting scaled BII values into BES forecast models using a posterior sampling method. Thus, projected changes represent changes in the measured ecosystem service response variable used in each BES relationship, rather than independent predictions of the underlying biological components contributing to that service". (Lines 560-563)*

11. L401: Does greater levels of pest regulation in countries with higher HDI values represent just a maintenance of current levels, or actual increases in this ES and "potential gains for agricultural systems"?

We thank the reviewer for highlighting this. Our analysis predicts relative changes in the predator: pest ratio between 1970 and 2050 rather than absolute levels of pest regulation. We have revised the text to clarify that higher HDI values are associated with greater maintenance of biological pest regulation (or smaller declines), rather than necessarily representing absolute increases in ecosystem service provision.

Revised text:

> *"By contrast, countries with higher HDI values are predicted to experience comparatively greater levels of biological pest regulation potential, reflecting greater capacity to maintain biological pest regulation". (Lines 325- 327)*

12. L522: Should this say "protection provided from pests by natural enemies"? I don't recall pathogens being mentioned in the main text of the manuscript.

Thanks for noticing this, you are correct. I have amended the Figure 4 caption as suggested.

Revised text:

> *"While the differences between these two scenarios for biodiversity are complex, our model forecasts that under each scenario, there will be subtly different degrees of protection provided from pests by natural enemies (A and B)". (Lines 700-702)*

13. L672-673: In the main text I think spatial grain is described as the spatial grain, but here "spatial extent" is also mentioned. Usually, a study will have both a spatial extent (to complete spatial extent of the measurements/plots across the whole study) and spatial grain (at what scale individual measurements are taken). It should be very clear what was actually measured, and how this was done (i.e., how was spatial grain determined for each paper).

Thank you for highlighting this distinction. We have removed the term "spatial extent" term and only refer to spatial grain.

Revised text:

> *"From each of the papers included in our database, we collected data on environmental context (ecosystem type, location, experimental treatment, land use, GPS, spatial grain or the size of the unit of measurement) and any experimental manipulation (i.e., temperature, biodiversity) and measurement type (experimental, observational)". (Lines 425-428)*

14. One last thought that I will leave to the authors to think about is around the robustness of your forecasted future changes to ecosystem services and the use of Biodiversity Intactness Index. A number of the BES and BEF relationships that were found are not linear, but are saturating or quadratic, which might reflect differential contributions of specific species to EF/ES based on functional traits. I am guessing (but might be wrong) that the predicted BII values don't include prediction about what specific species will be lost for each SSP. Thus, is it possible that the current modelling approach might under- or overestimate changes in EF/ES if species loss is not a random process across species in each scenario? If yes, then I think this could be something important to mention briefly in the paper as a limitation of the current modelling process. However, I might be misunderstanding the model construction and details, and in that case this comment can be ignored.

We thank the reviewer for this observation. You are correct that our modelling framework does not account for the identity of species projected to be lost under future scenarios but instead uses changes in Biodiversity Intactness Index as an aggregate measure of biodiversity change. Consequently, our projections implicitly assume that species' vulnerability to environmental change is independent of their contribution to ecosystem functioning and ecosystem services. We now address this in the methods and discussion of the manuscript; we paste the relevant sections below for review.

Revised text:

> *“Our projections assume that changes in BII are independent of the functional importance of the species affected. Consequently, projected changes in ecosystem services reflect aggregate changes in biodiversity rather than species-specific compositional turnover or differential contributions of individual species to ecosystem functioning”. (Lines 564-567)*
>
> *“An important limitation is that projected BII changes do not account for which species are lost under future scenarios. If species vulnerability is correlated with their functional contributions, future ecosystem service changes may differ from our projections. Future integration of species-level vulnerability and functional traits will be important for assessing how non-random biodiversity change influences biodiversity-ecosystem service relationships”. (Lines 328-332)*

Reviewer #2 (Remarks to the Author):

I would like to commend the authors on their thorough revisions to this paper. I would also like to reiterate the importance of this paper in providing a comprehensive synthesis of biodiversity-ecosystem service relationships, with direct implications for conservation policy. I believe the authors have now addressed all of my main concerns, as detailed below:

Introduction/framing: The new title is much improved and more representative of the broad scope of the paper.

Literature sampling: Additional clarity on the literature review search methods which has helped clarify how relevant papers were selected.

The role of biodiversity measures:
In response to my point about if the different measures of biodiversity were influencing the results, the authors conducted a new (thorough) analysis to answer this question. While they found that functional and phylogenetic diversity were disproportionately associated with saturating relationships (relative to taxonomic diversity), their dataset was dominated by taxonomic diversity measures, and as such they believe (and I agree) that strong claims cannot be made about metric differences. As such, they have included this analysis in the supplementary materials (with a mention in the main text) which is appropriate. This new analysis and findings do not change their main conclusions but add depth to the paper.

Ecosystem services are conflated with food production:
I was also concerned that their predictions of future change in pest control services were conflating the change in the ES with a change in agricultural output (another reviewer made a similar comment). They have now changed the how this analysis is described (including in the abstract), which I believe now makes it clear they are predicting a change in the ecosystem service. Predicting a change in actual agricultural output is well beyond the scope of this manuscript, so I believe their clarification of the future predictions is sufficient, and the change in the (biodiversity mediated) pest control ecosystem service is important and novel.

Climate scenarios:
The authors have now also now included the results for the 5 SSP scenarios and propagation of uncertainty which were described in the methods (along with further clarification of the methods). Including all 5 scenarios and the uncertainty surrounding them is an important addition to this modelling effort.

We thank Reviewer 2 for their thoughtful and constructive evaluation of our revised manuscript. We appreciate your recognition of the importance of this synthesis for advancing our understanding of biodiversity-ecosystem service relationships and its relevance to conservation

policy.

Reviewer #3 (Remarks to the Author):

I am satisfied with the reviewers' responses to my comments.

We thank Reviewer 3 for your constructive feedback. We are pleased that our revisions have addressed your concerns and greatly appreciate your positive assessment of the revised manuscript.
